# Analysis of the Spectrum of Doubly Ionized Molybdenum (Mo III)

**DOI:** 10.6028/jres.095.052

**Published:** 1990

**Authors:** L. Iglesias, M. I. Cabeza, V. Kaufman

**Affiliations:** Instituto de Optica, Serrano 121, 28006—Madrid, Spain; National Institute of Standards and Technology, Gaithersburg, MD 20899

**Keywords:** energy levels, molybdenum, parameters, spectra, wavelengths

## Abstract

The spectrum of doubly ionized molybdenum (Mo III) was produced in a sliding spark discharge and recorded photographically on the NIST 10.7-m normal incidence spectrograph in the 800-3250 Å spectral region. The analysis has led to the establishment of 76 levels of the interacting 4*d*^4^, 4*d*^3^ 5*s* and 4*d*^2^ 5*s*^2^ even configurations, 73 levels of the interacting 4*d*^3^ 5*d* and 4*d*^3^ 6*s* even configurations, and 181 levels of the interacting 4*d*^3^ 5*p* and 4*d*^2^ 5*s*5*p* odd configurations. Approximately 3100 lines have been classified as transitions between these experimentally determined levels. Comparison between the observed levels and those calculated from matrix diagonalizations with least-squares fitted parameters shows standard deviations of 44, 33, and 183 cm^−1^, respectively, for the levels of the three sets of configurations.

## 1. Introduction and Observations

In 1988 we published an analysis of the spectrum of doubly ionized molybdenum (Mo III) [1] in which a total of 679 spectral lines were classified. These were transitions between 54 levels of the 4*d*^4^ and 4*d*^3^ 5*s* even configurations and 65 levels of the 4*d*^3^ 5*p* odd configuration in that work.

We have now made additional observations in the range of 800–2100 Å to supplement our earlier data which covered the region 1100–3250 Å. These new observations were made under conditions similar to the previous ones but extended into the short wavelength region. The spectra were photographed on the NIST 10.7-m normal-incidence vacuum spectrograph equipped with a 1200-1/mm grating blazed at 1200 Å. A sliding spark operated at various excitation conditions was used to produce the spectra. The intensity distribution along each line and the behavior of the line intensity at 50, 80, and 150 A peak currents were used to find optimum conditions for the third spectrum. Reference wavelengths of Cu, Ge, and Si [2] were obtained with a water-cooled hollow cathode discharge. Details about the experimental methods are the same as given in reference [1]. Approximately 5000 of the observed lines had Mo III character. The wavelength uncertainty of the observed lines is estimated to be ±0.005 Å.

## 2. Analysis

The spectrum is complex due to the open 4*d*-shell structure of the doubly ionized atom; the ground configuration is 4*d*^4^. The large number of levels in the seven lowest configurations leads to many possible transitions. With the Cowan series of atomic structure programs [3], which include Hartree-Fock calculations with relativistic corrections (HFR) and matrix diagonalizations, we were able to predict the complete electric dipole spectrum. This included both the (4*d*^4^+4*d*^3^ 5*s*+4*d*^2^ 5*s*^2^)−(4*d*^3^ 5*p*+4*d*^2^ 5*s*5*p*) and the 4*d*^3^ 5*p*−(4*d*^3^ 5*d*+4*d*^3^ 6*s*) transition arrays. The observed line list and the line intensities were then compared to the predictions in order to extend the earlier analysis [1]. Calculations were made for each of the following interacting configuration groups: (1) 4*d*^4^+4*d*^3^ 5*s*+4*d*^2^ 5*s*^2^, (2) 4*d*^3^ 5*d*+4*d*^3^ 6*s*, and (3) 4*d*^3^ 5*p*+4*d*^2^ 5*s*5*p*. The resulting values for the radial integrals were adjusted by a least squares fit to the known levels, and improved as new levels were found.

This led to the identification of all 34 energy levels of 4*d*^4^ and all 38 energy levels of 4*d*^3^ 5*s*. The values of four of the previously reported levels were incorrect and have been replaced. They are the ^3^P_0_2, ^1^I_6_, ^1^D_2_2 and ^1^S_0_2 levels of 4*d*^4^. We use the index numbers assigned by Nielson and Koster [4] to distinguish recurring terms in the *d^n^* configurations. These index numbers were used by Martin et al. [5] in their compilation of atomic energy levels of the rare earth elements. All other previously reported level values were adjusted with the new data. Of the nine predicted levels of 4*d*^2^ 5*s*^2^, only those of the ^3^F, and the ^1^G_4_ have been located.

The 4*d*^3^(^2^H)5*d* ^3^K_8_ level has not been located. One strong transition is expected, but there are no appropriate lines (intensity, range, …) to establish it with certainty. For the 4*d*^3^(^2^H)5*d* ^1^K_7_, we have found a tentative energy value based on transitions with 4*d*^3^(^2^H)5*p* ^1^I_6_ and ^3^I_6_ at 1934.709 and 1808.672 Å, respectively. Because the second transition would be coincident with a second order Mo IV line, we consider the evidence for the level questionable.

**Figure 1:**
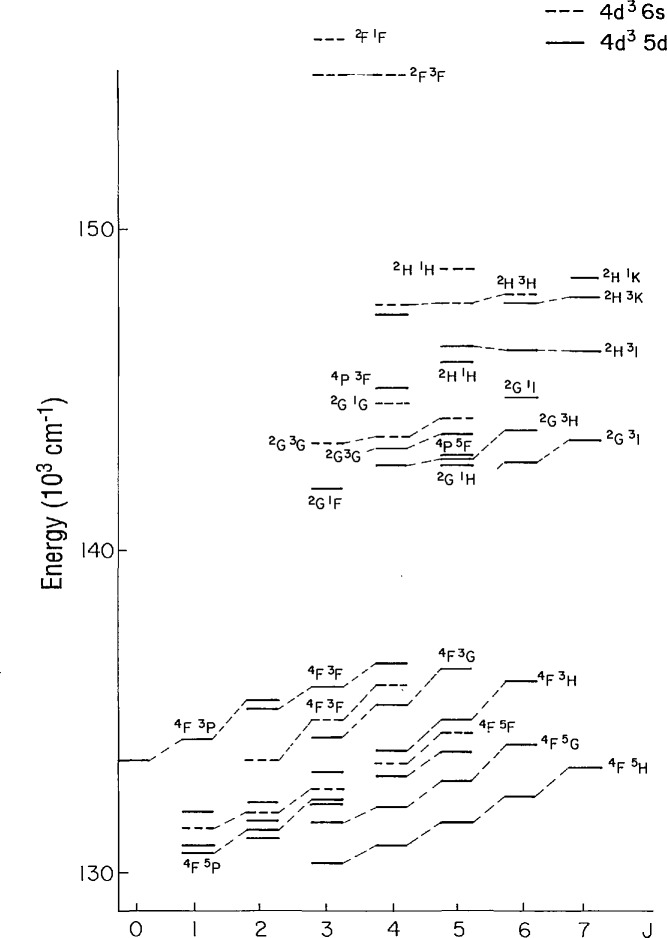
Observed energy levels of the 4*d*^3^ 5*d* and 4*d*^3^ 6*s* configurations. The levels are connected to show the *LS* terms.

We have found 54 levels of the 4*d*^3^ 5*d* configuration and 19 of 4*d*^3^ 6*s*. With the exception of 4*d*^3^(^4^F)5*d* ^5^D, all of the levels based on the 4*d*^3^(^4^F) parent have been found. These two configurations overlap extensively and similar terms of each configuration are very close. This accounts for the strong configuration interaction (CI). This may be seen in figure 1 where the levels are connected to show the LS terms.

Table 1 contains the 149 known levels of the five lowest even configurations, including for each level the configuration, term, *J* value, level value, difference between the observed level value and that obtained from the least-squares fits (O−C), and the leading eigenvector percentages in the *LS*-coupling scheme. The uncertainty in each level value depends on the number of combinations and on the wavelength region where the combinations appear. The uncertainties of the optimized energy-level values are generally less than ±0.10 cm^−1^ and no greater than ±0.20 cm^−1^. The average *LS* purities of the (4*d*^4^+4*d*^3^ 5*s*+4*d*^2^ 5*s*^2^) and (4*d*^3^ 5*d*+4*d*^3^ 6*s*) groups of configurations are 83% and 59%, respectively. Although 15 levels of 4*d*^3^ 5*d* and two of 4*d*^3^ 6*s* have their largest eigenvector components less than 50%, only five levels of 4*d*^3^ 5*d* have been given *LS* names that are not those of the largest eigenvector component.

**Figure 2:**
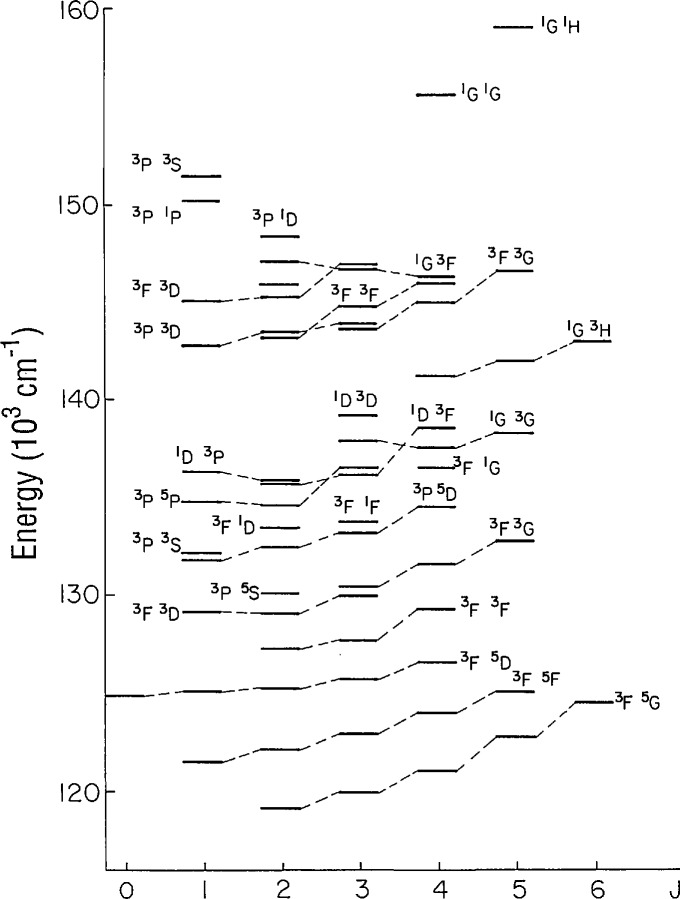
Observed energy levels of the 4*d*^2^ 5*s*5*p* configuration. The levels are connected to show the *LS* terms.

Table 2 contains the odd parity energy levels. Sixty-five levels of 4*d*^3^ 5*p* were included in the previous publication [1], but we have now found all 110 levels of this configuration. Seventy-one of the 90 predicted levels of 4*d*^2^ 5*s*5*p* were found through transitions with 4*d*^3^ 5*s* and 4*d*^2^ 5*s*^2^ levels in the vicinity of 1800 Å. The lowest levels of 4*d*^2^ 5*s*5*p* overlap with the highest levels of 4*d*^3^ 5*p*. The structure of the 4*d*^2^ 5*s*5*p* configuration is represented in figure 2. The combined average *LS* purity of the levels of these two odd configurations is 63%. Only four of the levels have been given *LS* names that are not associated with the largest eigenvector component.

A total of about 3100 spectral lines have been classified as transitions among the 330 levels. Table 3 includes all of the spectral lines classified as Mo III, giving for each the wavelength (in air above 2000 Å), intensity, wavenumber, difference between the observed wavelength and the wavelength obtained from the final level values (O−C), and its classification. The levels are denoted by their integer energy and *J* values.

The Cowan least-squares program [3] was used to fit the radial coefficients for each of the three sets of configurations to the observed energy levels. Tables 4, 5, and 6 include the least-squares fitted (LSF) and HFR values for the parameters of the (4*d*^4^+4*d*^3^ 5*s*+4*d*^2^ 5*s*^2^), the (4*d*^3^ 5*d*+4*d*^3^ 6*s*), and the (4*d*^3^ 5*p*+4*d*^2^ 5*s*5*p*) configuration groups. The ratios of the LSF to HFR values are also given. The standard deviations of the fits are 44, 33, and 183 cm^−1^, respectively.

## Figures and Tables

**Table 1:** Observed levels of the 4*d*^4^, 4*d*^3^ 5*s*, 4*d*^2^ 5*s*2, 4*d*^3^ 5*d*, and 4*d*^3^ 6*s* even configurations of doubly ionized molybdenum (Mo III)

Configuration	Term *J*	Level (cm^−1^)	O−C (cm^−1^)	Leading percentages^a^		
4*d*^4^	^5^D 0	0.0	−1	98		
	1	242.04	0	99		
	2	668.44	4	99		
	3	1223.96	12	100		
	4	1872.49	25	99		
4*d*^4^	^3^P2 0	11271.80	−3	58	35 ^3^P1	
	1	12510.23	2	62	34 ^3^P1	
	2	14357.56	7	63	32 ^3^P1	
4*d*^4^	^3^H 4	12679.70	22	85	7 ^3^G	
	5	13275.51	−4	94		
	6	13811.19	18	98		
4*d*^4^	^3^F2 2	13928.70	−22	75	21 ^3^F1	
	3	13948.27	−14	61	21 ^3^G	17 ^3^F1
	4	14296.10	14	63	14 ^3^F1	10 ^3^H
4*d*^4^	^3^G 3	15871.20	−41	78	18 ^3^F2	
	4	16282.70	−33	81	10 ^3^F2	
	5	16714.38	−18	93		
4*d*^4^	^3^D 3	19487.89	−3	97		
	2	19576.66	−5	90	5 ^1^D	
	1	19896.0	−19	98		
4*d*^4^	^1^I 6	19973.54	51	98		
4*d*^4^	^1^G2 4	20611.87	−8	63	28 ^1^G1	
4*d*^4^	^1^S2 0	22890.12	−31	77	20 ^1^S1	
4*d*^4^	^1^D2 2	23183.70	69	70	17 ^1^D1	6 ^3^D
4*d*^4^	^1^F 3	27006.61	40	94		
4*d*^4^	^3^P1 2	31323.10	46	63	32 ^3^P2	
	1	32519.35	4	61	34 ^3^P2	
	0	33155.4	−17	61	34 ^3^P2	
4*d*^4^	^3^F1 2	32387.45	−60	75	20 ^3^F2	
	4	32398.89	55	80	16 ^3^F2	
	3	32587.37	−15	76	18 ^3^F2	
4*d*^3^(^4^F)5*s*	^5^F 1	32418.68	−3	98		
	2	32843.28	4	98		
	3	33452.23	13	99		
	4	34225.38	24	99		
	5	35129.46	35	99		
4*d*^4^	^1^G1 4	36164.03	−23	64	28 ^1^G2	
4*d*^3^(^4^P)5*s*	^5^P 1	42404.71	−26	97		
	2	42665.77	−31	57	40 (^4^F)5*s* ^3^F	
	3	43461.62	0	89	10 (^4^F)5*s* ^3^F	
4*d*^3^(^4^F)5*s*	^3^F 2	42521.83	−57	56	38 (^4^P)5*s* ^5^P	
	3	43561.76	−35	84	11 (^4^P)5*s* ^5^P	
	4	44655.28	−20	90		
4*d*^3^(^2^G)5*s*	^3^G 3	46299.58	−37	96		
	4	46601.58	−43	87	6 (^4^F)5*s* ^3^F	
	5	46962.10	−58	88	9 (^2^H)5*s* ^3^H	
4*d*^4^	^1^D1 2	47978.47	−30	73	16 ^1^D2	3 (^2^D2)5*s* ^1^D
4*d*^3^(^2^P)5*s*	^3^P 1	48734.33	13	69	19 (^2^D2)5*s* ^3^D	6 (^2^D1)5*s* ^3^D
	0	48854.57	195	94		
	2	49088.73	26	63	20 (^2^D2)5*s* ^3^D	5 (^2^D1)5*s* ^3^D
4*d*^3^(^2^H)5*s*	^3^H 4	49541.67	55	85	10 (^2^G)5*s* ^1^G	
	5	50318.82	50	90		
	6	50481.62	36	100		
4*d*^3^(^2^D2)5*s*	^3^D 1	50362.58	−43	44	25 (^2^P)5*s* ^3^P	14 (^2^D1)5*s* ^3^D
	3	51425.90	−113	80	18 (^2^D1)5*s* ^3^D	
	2	51482.87	−30	55	24 (^2^P)5*s* ^3^P	16 (^2^D1)5*s* ^3^D
4*d*^3^(^2^G)5*s*	^1^G 4	52697.96	48	82	10 (^2^H)5*s* ^3^H	
4*d*^3^(^4^P)5*s*	^3^P 1	52811.06	0	63	22 (^2^P)5*s* ^1^P	9 (^2^D2)5*s* ^3^D
	0	53407.40	35	92		
	2	54191.24	61	90		
4*d*^3^(^2^H)5*s*	^1^H 5	54853.34	−12	99		
4*d*^3^(^2^P)5*s*	^1^P 1	55366.47	−8	64	30 (^4^P)5*s* ^3^P	
4*d*^3^(^2^D2)5*s*	^1^D 2	56741.89	11	69	18 (^2^D1)5*s* ^1^D	6 ^1^D
4*d*^3^(^2^F)5*s*	^3^F 4	58730.47	−53	98		
	3	58893.82	−27	98		
	2	59059.6	−12	98		
4*d*^4^	^1^S1 0	62879.75	−16	78	20 ^1^S2	
4*d*^3^(^2^F)5*s*	^1^F 3	64331.17	−21	97		
4*d*^3^(^2^D1)5*s*	^3^D 3	72187.93	27	81	18 (^2^D2)5*s* ^3^D	
	2	72356.47	10	78	21 (^2^D2)5*s* ^3^D	
	1	72481.84	−3	77	23 (^2^D2)5*s* ^3^D	
4*d*^3^(^2^D1)5*s*	^1^D 2	77557.42	0	77	21 (^2^D2)5*s* ^1^D	
4*d*^2^(^3^F)5*s*2	^3^F 2	88067.0	6	97		
	3	89482.54	0	99		
	4	91098.5	−6	98		
4*d*^2^(^1^G)5*s*2	^1^G 4	103485.73	−2	99		
4*d*^3^(^4^F)5*d*	^5^H 3	130365.61	36	95		
	4	130918.16	36	93		
	5	131607.85	35	93		
	6	132424.4	34	95		
	7	133337.7	36	99		
4*d*^3^(^4^F)5*d*	^3^D 1	130629.3^b^	−2	34	41 (^4^F)5*d* ^5^P	15 (^4^F)5*d* ^5^F
	2	131913.3^b^	−10	25	33 (^4^F)6*s* ^5^F	21 (^4^F)5*d* ^5^P
	3	133151.83^b^	−6	42	44 (^4^F)5*d* ^5^P	5 (^4^F)5*d* ^3^F
4*d*^3^(^4^F)5*d*	^5^P 1	130886.95	11	52	24 (^4^F)5*d* ^5^F	16 (^4^F)5*d* ^3^D
	2	131379.2	−3	54	18 (^4^F)5*d* ^5^G	11 (^4^F)5*d* ^3^D
	3	132337.96	−25	46	36 (^4^F)5*d* ^3^D	6 (^4^F)5*d* ^5^G
4*d*^3^(^4^F)5*d*	^5^G 2	131072.5	−26	30	28 (^4^F)5*d* ^5^F	24 (^4^F)5*d* ^3^D
	3	131592.6	−22	52	39 (^4^F)5*d* ^5^F	
	4	132173.9	−20	51	39 (^4^F)5*d* ^5^F	4 (^4^F)5*d* ^5^H
	5	132951.3	−17	55	34 (^4^F)5*d* ^5^F	5 (^4^F)5*d* ^5^H
	6	134010.4	−19	90		
4*d*^3^(^4^F)6*s*	^5^F 1	131396.0	14	75	17 (^4^F)5*d* ^3^D	
	2	131647.45	2	40	38 (^4^F)5*d* ^5^G	15 (^4^F)5*d* ^5^F
	3	132279.6	3	42	24 (^4^F)5*d* ^5^G	17 (^4^F)5*d* ^5^F
	4	133446.5	−9	63	25 (^4^F)5*d* ^5^F	8 (^4^F)5*d* ^5^G
	5	134371.48	1	83	12 (^4^F)5*d* ^5^F	
4*d*^3^(^4^F)5*d*	^5^F 1	131900.8	−10	56	22 (^4^F)5*d* ^3^D	17 (^4^F)6*s* ^5^F
	2	132228.4	−12	38	26 (^4^F)5*d* ^3^D	15 (^4^F)6*s* ^5^F
	3	132666.7^b^	−16	36	44 (^4^F)6*s* ^5^F	14 (^4^F)5*d* ^5^G
	4	133034.7^b^	0	30	34 (^4^F)5*d* ^5^G	31 (^4^F)6*s* ^5^F
	5	133782.3	−5	47	34 (^4^F)5*d* ^5^G	14 (^4^F)6*s* ^5^F
4*d*^3^(^4^F)5*d*	^3^P 0	133508.9	−36	91		
	1	134185.0	−26	83	4 (^2^P)5*d* ^3^P	4 (^4^F)5*d* ^3^D
	2	135441.05	−39	74	7 (^4^F)5*d* ^3^F	5 (^4^F)5*d* ^3^D
4*d*^3^(^4^F)6*s*	^3^F 2	133563.4	−2	91		
	3	134665.06	−5	88	4 (^4^F)6*s* ^5^F	
	4	135857.46	13	80	7 (^4^F)5*d* ^3^F	6 (^4^F)5*d* ^3^G
4*d*^3^(^4^F)5*d*	^3^H 4	133739.17	66	87	4 (^2^H)5*d* ^3^H	
	5	134799.5	55	87	4 (^2^H)5*d* ^3^H	
	6	135979.5	47	87	5 (^4^F)5*d* ^5^G	
4*d*^3^(^4^F)5*d*	^3^G 3	134295.5	−17	79	4 (^2^G)5*d* ^3^G	4 (^4^F)5*d* ^3^F
	4	135261.56	−19	74	10 (^4^F)5*d* ^3^F	3 (^4^F)5*d* ^5^F
	5	136391.56	−30	85	3 (^4^F)5*d* ^S^F	3 (^2^G)5*d* ^3^G
4*d*^3^(^4^F)5*d*	^3^F 2	13511Z06	6	72	9 (^4^F)5*d* ^3^P	4 (^2^G)5*d* ^3^F
	3	135882.9	5	71	5 (^4^F)5*d* ^3^D	5 (^2^G)5*d* ^3^F
	4	136574.54	−11	66	13 (^4^F)6*s* ^3^F	6 (^4^F)5*d* ^3^G
4*d*^3^(^2^G)5*d*	^1^F 3	141993.9	−36	27	17 (^4^P)5*d* ^3^D	16 (^2^G)5*d* ^3^G
4*d*^3^(^2^G)5*d*	^3^H 4	142696.6	19	62	14 (^2^H)5*d* ^3^H	13 (^2^G)5*d* ^3^G
	5	142946.75	38	32	24 (^2^G)5*d* ^3^G	10 (^4^P)5*d* ^5^F
	6	143829.4	6	64	11 (^2^H)5*d* ^3^I	9 (^2^H)5*d* ^3^H
4*d*^3^(^2^G)5*d*	^1^H 5	142712.95	−25	69	10 (^2^G)5*d* ^3^H	6 (^2^G)5*d* ^3^I
4*d*^3^(^2^G)5*d*	^3^I 6	142822.32	−43	87	8 (^2^H)5*d* ^3^I	
	7	143528.65	−19	87	5 (^2^H)5*d* ^3^K	
4*d*^3^(^4^P)5*d*	^5^F 5	142950.65	−0	85	4 (^2^G)5*d* ^3^G	2 (^2^G)5*d* ^3^H
*4d*^3^(^2^G)5*d*	^3^G 4	143198.04	−11	52	19 (^2^G)5*d* ^3^H	11 (^2^G)6*s* ^3^G
	5	143653.5	−9	34	30 (^2^G)5*d* ^3^H	11 (^2^H)5*d* ^3^I
*4d*^3^(^2^G)6*s*	^3^G 3	143396.8	118	79	9 (^2^G)5*d* ^1^F	6 (^2^G)5*d* ^3^G
	4	143568.8	−11	70	11 (^2^G)5*d* ^3^G	10 (^2^G)6*s* ^1^G
	5	144121.65	−13	79	8 (^2^H)6*s* ^3^H	5 (^2^G)5*d* ^3^G
*4d*^3^(^2^G)6*s*	^1^G 4	144656.26	−6	58	13 (^2^H)6*s* ^3^H	6 (^4^P)5*d* ^5^D
*4d*^3^(^2^G)5*d*	^1^I 6	144783.96	5	61	13 (^2^H)5*d* ^3^K	11 (^2^H)5*d* ^1^I
*4d*^3^(^4^P)5*d*	^3^F 4	145096.74	−15	55	11 (^2^P)5*d* ^3^F	8 (^2^H)5*d* ^3^F
*4d*^3^(^2^H)5*d*	^1^H 5	145904.28	−7	67	19 (^2^H)5*d* ^3^I	7 (^2^G)5*d* ^3^G
*4d*^3^(^2^H)5*d*	^3^I 7	146257.14	−19	94		
	6	146277.52	−22	73	13 (^2^G)5*d* ^3^H	5 (^2^G)5*d* ^3^I
	5	146342.74	30	51	15 (^2^G)5*d* ^1^H	14 (^2^H)5*d* ^1^H
*4d*^3^(^2^D2)5*d*	^3^G 4	147431.23	48	38	22 (^2^H)5*d* ^3^G	11 (^2^G)5*d* ^3^F
*4d*^3^(^2^H)6*s*	^3^H 4	147703.6	−11	75	12 (^2^G)6*s* ^1^G	7 (^2^G)6*s* ^3^G
	5	147752.1	−11	82	10 (^2^G)6*s* ^3^G	
	6	147984.1	2	96		
*4d*^3^(^2^H)5*d*	^3^K 6	147758.2	−2	83	8 (^2^G)5*d* ^1^I	
	7	147963.3	6	90	7 (^2^G)5*d* ^3^I	
*4d*^3^(^2^H)5*d*	^1^K 7	148595.3	35	93		
*4d*^3^(^2^H)6*s*	^1^H 5	148816.1	21	90	4 (^2^H)6*s* ^3^H	
*4d*^3^(^2^H)5*d*	^3^H 5	151580.2	−46	48	17 (^2^H)5*d* ^3^G	9 (^2^G)5*d* ^3^G
*4d*^3^(^2^F)6*s*	^3^F 4	156378.82	−40	92		
	3	156587.8	51	87	8 (^2^F)5*d* ^3^F	
4*d*^3^(^2^F)6*s*	^1^F 3	157546.6	−25	84	6 (^2^F)5*d* ^3^D	

a The second and/or the third eigenvector component has been omitted when the first one or two components amount to 90% or greater.

b This level is not given the *LS* name corresponding to the largest eigenvector component.

**Table 2:** Observed levels of the 4*d*^3^ 5*p* and 4*d*^2^ 5*s*5*p* odd configurations of doubly ionized molybdenum (Mo III)

Configuration	Term *J*	Level (cm^−1^)	O−C (cm^−1^)	Leading percentages^a^		
4*d*^3^(^4^F)5*p*	^5^G 2	73853.18	−65	96		
	3	74724.72	−73	97		
	4	75816.51	−75	97		
	5	77113.28	−62	96		
	6	78689.51	−09	99		
4*d*^3^(^4^F)5*p*	^3^D 1	75972.36	−6	56	31 (^4^F)5*p* ^5^F	5 (^4^P)5*p* ^3^D
	2	76836.82	−43	47	32 (^4^F)5*p* ^5^F	11 (^4^F)5*p* ^5^D
	3	80354.49	−120	41	36 (^4^F)5*p* ^5^D	7 (^4^P)5*p* ^3^D
4*d*^3^(^4^F)5*p*	^5^F 3	78158.42	−57	42	27 (^4^F)5*p* ^3^D	23 (^4^F)5*p* ^5^D
	1	78677.94	102	56	26 (^4^F)5*p* ^5^D	13 (^4^F)5*p* ^3^D
	2	79013.98	99	61	22 (^4^F)5*p* ^5^D	11 (^4^F)5*p* ^3^D
	4	79497.10	−32	70	19 (^4^F)5*p* ^5^D	6 (^4^F)5*p* ^3^G
	5	80343.19	15	73	17 (^4^F)5*p* ^3^G	7 (^2^G)5*p* ^3^G
4*d*^3^(^4^F)5*p*	^5^D 0	78568.37	9	91		
	1	78947.76	−29	62	15 (^4^F)5*p* ^3^D	12 (^4^F)5*p* ^5^F
	2	79467.93	−87	59	19 (^4^F)5*p* ^3^D	7 (^4^P)5*p* ^5^D
	3	79508.33^b^	61	33	48 (^4^F)5*p* ^5^F	9 (^4^F)5*p* ^3^D
	4	80095.61	−48	70	15 (^4^F)5*p* ^5^F	4 (^4^F)5*p* ^3^F
4*d*^3^(^4^F)5*p*	^3^G 3	81040.69	−43	74	16 (^2^G)5*p* ^3^G	5 (^4^F)5*p* ^5^F
	4	82009.90	−79	66	14 (^4^F)5*p* ^5^F	13 (^2^G)5*p* ^3^G
	5	83147.76	−116	55	25 (^4^F)5*p* ^5^F	10 (^2^G)5*p* ^3^G
4*d*^3^(^4^F)5*p*	^3^F 2	82540.14	42	76	8 (^2^D2)5*p* ^3^F	4 (^4^F)5*p* ^3^D
	3	83584.53	8	79	7 (^2^D2)5*p* ^3^F	3 (^4^F)5*p* ^3^D
	4	84544.52	0	80	6 (^2^D2)5*p* ^3^F	3 (^4^F)5*p* ^5^D
4*d*^3^(^2^P)5*p*	^1^S 0	84216.41^b^	−275	29	33 (^4^P)5*p* ^3^P	25 (^4^P)5*p* ^5^D
4*d*^3^(^4^P)5*p*	^5^P 1	85308.94	66	69	13 (^4^P)5*p* ^5^D	7 (^2^P)5*p* ^3^P
	2	86426.81	256	86	8 (^4^P)5*p* ^3^D	
	3	87391.79	298	92		
4*d*^3^(^4^P)5*p*	3p 2	85329.99	−114	44	20 (^4^P)5*p* ^5^D	9 (^2^P)5*p* ^3^P
	1	87831.20	−50	55	27 (^4^P)5*p* ^5^D	8 (^2^D2)5*p* ^3^P
	0	89775.81	−153	32	28 (^2^P)5*p* ^3^P	26 (^2^P)5*p* ^1^S
4*d*^3^(^4^P)5*p*	^5^D 1	85683.11	200	41	28 (^4^P)5*p* ^3^P	18 (^4^P)5*p* ^5^P
	0	86322.66	−170	58	27 (^2^P)5*p* ^1^S	6 (^4^F)5*p* ^5^D
	2	87473.30	5	58	15 (^4^P)5*p* ^3^P	8 (^4^P)5*p* ^5^P
	3	87810.66	90	80	6 (^4^F)5*p* ^5^D	5 (^2^P)5*p* ^3^D
	4	89100.17	10	92		
4*d*^3^(^2^G)5*p*	^3^H 4	85896.20	−132	69	24 (^2^H)5*p* ^3^H	
	5	86892.62	−119	56	26 (^2^H)5*p* ^3^H	8 (^4^F)5*p* ^3^G
	6	88441.64	−62	59	33 (^2^H)5*p* ^3^H	
4*d*^3^(^2^G)5*p*	^1^F 3	88499.21	−146	30	29 (^2^G)5*p* ^3^G	18 (^2^G)5*p* ^3^F
4*d*^3^(^2^P)5*p*	^1^D 2	88592.07	−123	26	13 (^4^P)5*p* ^3^P	11 (^2^D2)5*p* ^1^D
4*d*^3^(^2^P)5*p*	^3^P 0	88669.74	147	36	19 (^2^D2)5*p* ^3^P	19 (^4^P)5*p* ^3^P
	1	89139.81	163	33	14 (^2^D2)5*p* ^3^P	12 (^2^P)5*p* ^3^D
	2	90982.60	57	32	17 (^2^D2)5*p* ^3^P	13 (^4^P)5*p* ^5^S
4*d*^3^(^2^G)5*p*	^1^H 5	89689.91	−31	40	29 (^2^H)5*p* ^1^H	13 (^2^H)5*p* ^3^I
4*d*^3^(^2^P)5*p*	^3^D 1	90301.83	262	62	13 (^2^P)5*p* ^3^P	7 (^4^F)5*p* ^3^D
	2	91674.55	141	57	11 (^2^G)5*p* ^3^F	5 (^4^P)5*p* ^5^S
	3	92758.61	116	48	19 (^2^G)5*p* ^3^F	12 (^2^D2)5*p* ^3^F
4*d*^3^(^2^G)5*p*	^3^G 4	90255.05	−101	63	16 (^2^G)5*p* ^1^G	13 (^4^F)5*p* ^3^G
	3	90588.46	−119	48	19 (^2^G)5*p* ^1^F	9 (^2^G)5*p* ^3^F
	5	91006.90	−92	57	12 (^2^H)5*p* ^3^I	10 (^2^H)5*p* ^3^H
4*d*^3^(^2^G)5*p*	^3^F 4	89503.85	162	32	27 (^2^G)5*p* ^1^G	14 (^2^H)5*p* ^3^H
	2	90586.00	−53	45	11 (^2^D2)5*p* ^3^F	10 (^2^P)5*p* ^1^D
	3	91050.30	−7	35	20 (^2^G)5*p* ^1^F	13 (^2^D2)5*p* ^3^F
4*d*^3^(^2^H)5*p*	^3^H 4	91387.50^b^	117	35	39 (^2^G)5*p* ^3^F	12 (^2^G)5*p* ^3^H
	5	92254.52	82	50	26 (^2^G)5*p* ^3^H	13 (^2^H)5*p* ^3^I
	6	92728.95	170	56	34 (^2^G)5*p* ^3^H	
4*d*^3^(^4^P)5*p*	^5^S 2	92099.55	−90	75	7 (^2^P)5*p* ^3^P	5 (^4^P)5*p* ^3^P
4*d*^3^(^2^H)5*p*	^3^I 5	92884.18	−28	57	16 (^2^G)5*p* ^1^H	9 (^2^G)5*p* ^3^G
	6	93306.10	20	91		
	7	94424.07	27	100		
4*d*^3^(^2^G)5*p*	^1^G 4	93102.01	20	51	15 (^2^H)5*p* ^3^H	7 (^2^G)5*p* ^3^H
4*d*^3^(^2^P)5*p*	^3^S 1	93222.37	256	75	9 (^2^P)5*p* ^3^P	4 (^2^P)5*p* ^1^P
4*d*^3^(^2^D2)5*p*	^3^F 2	93642.52	−31	40	28 (^2^G)5*p* ^3^F	9 (^2^P)5*p* ^3^D
	3	94117.58	−14	26	21 (^2^D2)5*p* ^3^D	14 (^2^P)5*p* ^3^D
	4	94955.85	−85	63	12 (^2^D1)5*p* ^3^F	8 (^2^G)5*p* ^3^F
4*d*^3^(^2^D2)5*p*	^1^P 1	93709.46^b^	96	20	35 (^2^P)5*p* ^1^P	17 (^2^D2)5*p* ^3^D
4*d*^3^(^2^H)5*p*	^1^G 4	94098.26	−94	56	22 (^2^F)5*p* ^1^G	7 (^2^H)5*p* ^3^H
4*d*^3^(^2^D2)5*p*	^3^D 1	94292.66	121	48	16 (^2^D2)5*p* ^1^P	7 (^2^D1)5*p* ^3^D
	2	95551.80	128	47	24 (^4^P)5*p* ^3^D	12 (^2^D1)5*p* ^3^D
	3	95856.45	119	34	24 (^4^P)5*p* ^3^D	7 (^2^D1)5*p* ^5^D
4*d*^3^(^4^P)5*p*	^3^D 2	94387.70	−33	43	20 (^2^D2)5*p* ^3^D	13 (^2^D2)5*p* ^3^P
	3	94676.73	74	47	18 (^2^D2)5*p* ^3^D	12 (^2^P)5*p* ^3^D
	1	95016.32	152	67	10 (^2^P)5*p* ^3^D	7 (^2^D2)5*p* ^1^P
4*d*^3^(^2^H)5*p*	^3^G 5	96285.38	171	49	24 (^2^H)5*p* ^1^H	14 (^2^G)5*p* ^1^H
	3	96838.34	158	60	10 (^2^F)5*p* ^3^G	7 (^2^D2)5*p* ^1^F
	4	97184.77	137	72	11 (^2^F)5*p* ^3^G	4 (^2^G)5*p* ^3^F
4*d*^3^(^2^D2)5*p*	^3^p 2	96589.89^b^	−191	31	32 (^2^P)5*p* ^3^P	15 (^4^P)5*p* ^3^P
	1	96736.45	−101	34	15 (^2^P)5*p* ^3^P	12 (^2^D2)5*p* ^1^P
	0	97135.60	−334	47	26 (^2^P)5*p* ^3^P	13 (^4^P)5*p* ^3^P
4*d*^3^(^2^H)5*p*	^1^I 6	96907.92	93	90		
4*d*^3^(^2^H)5*p*	^1^H 5	97709.08	45	37	33 (^2^H)5*p* ^3^G	24 (^2^G)5*p* ^1^H
4*d*^3^(^2^D2)5*p*	^1^F 3	98562.38	203	42	16 (^2^G)5*p* ^1^F	12 (^2^H)5*p* ^3^G
4*d*^3^(^2^P)5*p*	^1^P 1	99313.02	−51	43	8 (^2^D2)5*p* ^3^P	8 (^4^P)5*p* ^3^S
4*d*^3^(^2^F)5*p*	^3^F 2	99952.26	−83	64	11 (^2^P)5*p* ^1^D	8 (^2^D2)5*p* ^1^D
	3	100397.67	75	77	9 (^2^F)5*p* ^3^G	3 (^2^D2)5*p* ^3^F
	4	100858.67	88	77	10 (^2^F)5*p* ^3^G	3 (^2^G)5*p* ^3^F
4*d*^3^(^4^P)5*p*	^3^S 1	100184.65	134	74	12 (^2^P)5*p* ^1^P	4 (^2^D2)5*p* ^1^P
4*d*^3^(^2^D2)5*p*	^1^D 2	100219.97	−153	36	30 (^2^P)5*p* ^1^D	17 (^2^F)5*p* ^3^F
4*d*^3^(^2^F)5*p*	^3^G 3	102557.67	−18	70	12 (^2^H)5*p* ^3^G	8 (^2^F)5*p* ^3^F
	4	103276.74	46	72	13 (^2^F)5*p* ^3^F	10 (^2^H)5*p* ^3^G
	5	103621.4	43	90	10 (^2^H)5*p* ^3^G	
4*d*^3^(^2^F)5*p*	^1^D 2	103303.98	−109	59	28 (^2^D2)5*p* ^1^D	9 (^2^F)5*p* ^3^F
4*d*^3^(^2^F)5*p*	^3^D 3	103667.40	−169	73	9 (^2^D2)5*p* ^3^D	5 (^2^F)5*p* ^3^F
	2	104511.12	−216	84	7 (^2^D2)5*p* ^3^D	
	1	105041.26	−229	87	7 (^2^D2)5*p* ^3^D	
4*d*^3^(^2^F)5*p*	^1^G 4	106511.94	118	70	24 (^2^H)5*p* ^1^G	
4*d*^3^(^2^F)5*p*	^1^F 3	106803.63	−299	84	4 (^2^D2)5*p* ^1^F	4 (^2^F)5*p* ^3^D
4*d*^3^(^2^D1)5*p*	^3^D 1	114014.74	−80	73	17 (^2^D2)5*p* ^3^D	4 (^2^F)5*p* ^3^D
	2	114083.06	−51	69	13 (^2^D2)5*p* ^3^D	6 (^2^D1)5*p* ^3^P
	3	114591.26	−17	69	10 (^2^D2)5*p* ^3^D	7 (^2^D1)5*p* ^3^F
4*d*^3^(^2^D1)5*p*	^3^F 2	115794.02	−102	55	19 (^2^D2)5*p* ^3^F	8 (^2^D1)5*p* ^1^D
	3	116497.95	69	59	18 (^2^D2)5*p* ^3^F	10 (^2^D1)5*p* ^3^D
	4	117287.80	101	76	20 (^2^D2)5*p* ^3^F	
4*d*^3^(^2^D1)5*p*	^1^D 2	117336.75	−176	46	17 (^2^D2)5*p* ^1^D	17 (^2^F)5*p* ^1^D
4*d*^3^(^2^D1)5*p*	^3^P 2	118451.23	177	70	18 (^2^D2)5*p* ^3^P	5 (^2^D1)5*p* ^3^D
	1	119206.22	148	72	22 (^2^D2)5*p* ^3^P	
	0	119559.55	140	74	24 (^2^D2)5*p* ^3^P	
4*d*^2^(^2^F)5*s*5*p*(^3^P°)	^5^G 2	119170.3	−234	95		
	3	120064.7	−225	95		
	4	121118.4	−356	96		
	5	122817.2	−110	96		
	6	124605.7	−52	100		
4*d*^3^(^2^D1)5*p*	^1^F 3	119479.53	37	71	15 (^2^D2)5*p* ^1^F	7 (^2^D1)5*p* ^3^F
4*d*^2^(^3^F)5*s*5*p*(^3^P°)	^5^F 1	121723.8	102	96		
	2	122229.55	92	94		
	3	123007.56	83	94		
	4	124005.8	79	94		
	5	125143.67	84	94		
4*d*^3^(^2^D1)5*p*	^1^P 1	124221.46	−231	72	25 (^2^D2)5*p* ^1^P	
4*d*^2^(^3^F)5*s*5*p*(^3^P°)	^5^D 0	124982.8	22	83	16 (^3^P)5*s*5*p* ^5^D	
	1	125107.68	31	82	14 (^3^P)5*s*5*p* ^5^D	
	2	125359.42	43	79	11 (^3^P)5*s*5*p* ^5^D	3 (^3^F)5*s*5*p* ^5^F
	3	125786.8	52	78	8 (^3^P)5*s*5*p* ^5^D	3 (^3^F)5*s*5*p* ^3^D
	4	126533.5	79	84	8 (^3^P)5*s*5*p* ^5^D	
4*d*^2^(^3^F)5*s*5*p*(^3^P°)	^3^F 2	127336.03	−241	35	35 (^3^F)5*s*5*p* ^3^F	17 (^1^D)5*s*5*p* ^3^F
	3	127795.88	−152	24	23 (^3^F)5*s*5*p* ^3^F	14 (^1^D)5*s*5*p* ^3^F
	4	129383.82	−122	33	33 (^3^F)5*s*5*p* ^3^F	14 (^1^D)5*s*5*p* ^3^F
4*d*^2^(^3^F)5*s*5*p*(^3^P°)	^3^D 2	129055.2	−39	30	26 (^3^F)5*s*5*p* ^3^D	10 (^3^P)5*s*5*p* ^3^D
	1	129065.63	−17	38	26 (^3^F)5*s*5*p* ^3^D	12 (^3^P)5*s*5*p* ^3^D
	3	129964.64	−36	26	25 (^3^F)5*s*5*p* ^3^D	9 (^3^P)5*s*5*p* ^3^D
4*d*^2^(^3^P)5*s*5*p*(^3^P°)	^5^S 2	130073.7	−602	92		
4*d*^2^(^3^F)5*s*5*p*(^3^P°)	^3^G 3	130453.9	104	51	21 (^3^F)5*s*5*p* ^1^G	8 (^3^F)5*s*5*p* ^3^F
	4	131570.80	63	50	28 (^3^F)5*s*5*p* ^3^G	10 (^1^G)5*s*5*p* ^3^G
	5	132792.84	18	48	32 (^3^F)5*s*5*p* ^3^G	15 (^1^G)5*s*5*p* ^3^G
4*d*^2^(^3^P)5*s*5*p*(^3^P°)	^5^D 1	131782.5	151	79	16 (^3^F)5*s*5*p* ^5^D	
	2	132439.5	147	74	15 (^3^F)5*s*5*p* ^S^D	3 (^3^F)5*s*5*p* ^1^D
	3	133255.4	181	49	34 (^3^F)5*s*5*p* ^1^F	7 (^3^F)5*s*5*p* ^5^D
	4	134502.10	236	76	10 (^3^F)5*s*5*p* ^5^D	6 (^1^D)5*s*5*p* ^3^F
4*d*^2^(^3^P)5*s*5*p*(^3^P°)	^3^S 1	132164.6	209	49	30 (^3^P)5*s*5*p* ^3^S	16 (^3^P)5*s*5*p* ^5^P
4*d*^2^(^3^F)5*s*5*p*(^3^P°)	^1^D 2	133422.2	−60	55	17 (^3^P)5*s*5*p* ^1^D	4 (^3^F)5*s*5*p* ^3^F
4*d*^2^(^3^F)5*s*5*p*(^3^P°)	^1^F 3	133818.4	166	44	28 (^3^P)5*s*5*p* ^5^D	6 (^3^F)5*s*5*p* ^5^D
4*d*^2^(^3^P)5*s*5*p*(^3^P°)	^5^P 2	134695.4	−162	45	33 (^1^D)5*s*5*p* ^3^P	7 (^1^D)5*s*5*p* ^3^D
	1	134844.9	48	66	20 (^1^D)5*s*5*p* ^3^P	4 (^1^D)5*s*5*p* ^3^D
	3	136281.5	226	85	8 (^1^D)5*s*5*p* ^3^D	
4*d*^2^(^1^D)5*s*5*p*(^3^P°)	^3^F 2	135721.81	207	73	17 (^3^F)5*s*5*p* ^3^F	
	3	136402.5	228	66	13 (^3^F)5*s*5*p* ^3^F	9 (^1^G)5*s*5*p* ^3^G
	4	138688.1	157	35	23 (^1^D)5*s*5*p* ^3^G	17 (^3^F)5*s*5*p* ^1^G
4*d*^2^(^1^D)5*s*5*p*(^3^P°)	^3^P 2	135963.7^b^	106	43	49 (^3^P)5*s*5*p* ^5^P	
	1	136300.2	−113	42	24 (^1^D)5*s*5*p* ^3^D	17 (^3^P)5*s*5*p* ^5^P
4*d*^2^(^3^F)5*s*5*p*(^3^P°)	^1^G 4	136575.7	277	51	27 (^1^D)5*s*5*p* ^3^F	9 (^3^P)5*s*5*p* ^5^D
4*d*^2^(^1^G)5*s*5*p*(^3^P°)	^3^G 4	137605.1	161	51	21 (^3^F)5*s*5*p* ^1^G	13 (^3^F)5*s*5*p* ^3^G
	3	137796.5	52	66	18 (^3^F)5*s*5*p* ^3^G	7 (^1^D)5*s*5*p* ^3^F
	5	138344.9	49	76	20 (^3^F)5*s*5*p* ^3^G	
4*d*^2^(^1^D)5*s*5*p*(^3^P°)	^3^D 3	139243.0	3	76	12 (^3^P)5*s*5*p* ^5^P	5 (^3^F)5*s*5*p* ^3^D
4*d*^2^(^1^G)5*s*5*p*(^3^P°)	^3^H 4	141176.2	−26	92		
	5	141967.4	−54	96		
	6	142940.8	−76	100		
4*d*^2^(^3^P)5*s*5*p*(^3^P°)	^3^D 1	142845.9	94	58	15 (^3^P)5*s*5*p* ^3^D	10 (^1^D)5*s*5*p* ^3^D
	2	143585.8	26	33	17 (^3^F)5*s*5*p* ^3^F	16 (^3^P)5*s*5*p* ^3^D
	3	143809.26	111	34	28 (^3^P)5*s*5*p* ^3^D	15 (^3^F)5*s*5*p* ^3^D
4*d*^2^(^3^F)5*s*5*p*(^1^P°)	^3^F 2	143204.05	169	34	29 (^3^F)5*s*5*p* ^3^F	16 (^3^P)5*s*5*p* ^3^D
	3	144812.5	73	40	31 (^3^F)5*s*5*p* ^3^F	7 (^3^F)5*s*5*p* ^3^G
	4	145951.98^b^	2	26	38 (^1^G)5*s*5*p* ^3^F	26 (^3^F)5*s*5*p* ^3^F
4*d*^2^(^3^F)5*s*5*p*(^1^P°)	^3^G 3	143701.9	222	58	16 (^3^F)5*s*5*p* ^3^G	8 (^1^G)5*s*5*p* ^3^G
	4	145075.7	374	62	23 (^3^F)5*s*5*p* ^3^G	5 (^1^G)5*s*5*p* ^3^G
	5	146655.7	494	63	30 (^3^F)5*s*5*p* ^3^G	
4*d*^2^(^3^F)5*s*5*p*(^1^P°)	^3^D 1	145036.8	−282	39	28 (^3^F)5*s*5*p* ^3^D	11 (^3^P)5*s*5*p* ^3^P
	2	145978.7	−608	31	29 (^3^F)5*s*5*p* ^3^D	14 (^3^P)5*s*5*p* ^3^P
	3	146972.3	−924	41	27 (^3^F)5*s*5*p* ^3^D	22 (^3^P)5*s*5*p* ^3^D
4*d*^2^(^3^P)5*s*5*p*(^1^P°)	^3^P 2	145347.6	−161	43	24 (^3^P)5*s*5*p* ^3^P	8 (^3^F)5*s*5*p* ^3^D
4*d*^2^(^1^G)5*s*5*p*(^1^P°)	^3^F 4	146336.45	−211	52	23 (^3^F)5*s*5*p* ^3^F	14 (^3^F)5*s*5*p* ^3^F
	3	146868.7	−302	90		
	2	147182.9	−254	77	13 (^3^P)5*s*5*p* ^1^D	3 (^3^F)5*s*5*p* ^1^D
4*d*^2^(^3^P)5*s*5*p*(^1^P°)	^1^D 2	148421.6	−158	50	18 (^1^G)5*s*5*p* ^3^F	18 (^3^F)5*s*5*p* ^1^D
4*d*^2^(^3^P)5*s*5*p*(^1^P°)	^1^P 1	150204.2	177	80	7 (^3^P)5*s*5*p* ^3^S	7 (^3^P)5*s*5*p* ^3^S
4*d*^2^(^3^P)5*s*5*p*(^1^P°)	^3^S 1	151380.2	234	42	23 (^3^P)5*s*5*p* ^3^S	18 (^1^D)5*s*5*p* ^1^P
4*d*^2^(^1^D)5*s*5*p*(^1^P°)	^1^F 3	153104.6	−5	82	3 (^3^F)5*s*5*p* ^3^D	3 (^3^F)5*s*5*p* ^1^F
4*d*^2^(^1^G)5*s*5*p*(^1^P°)	^1^G 4	155674.86	250	94		
4*d*^2^(^1^G)5*s*5*p*(^1^P°)	^1^H 5	159013.92	64	98		
4*d*^2^(^1^G)5*s*5*p*(^1^P°)	^1^F 3	164339.5	−99	93		

a The second and/or the third component has been omitted when the first one or two components amount to 90% or greater.

b This level is not given the *LS* name corresponds to the largest eigenvector component.

**Table 3:** Classified lines of Mo III

Wavelength (Å)	Int.^a^	Wavenumber (cm^−1^)	O−C (Å)	Classification			Wavelength (Å)	Int.^a^	Wavenumber (cm^−1^)	O−C (Å)	Classification		
				Level *J*	–	Level *J*					Level *J*	–	Level *J*
836.749	1	119510.15	.005	36164 4	–	155674° 4	997.236	5	100277.16	−.004	34225 4	–	134502° 4
848.510	1	117853.64	.001	13928 2	–	131782° 1	997.332	1	100267.51	−.004	46601 4	–	146868° 3
855.135	2	116940.60	.000	36164 4	–	153104° 3	997.525	2	100248.11	−.006	43561 3	–	143809° 3
861.602	5bl	116062.86	.007	1223 3	–	117287° 4	997.598	20	100240.77	−.005	43461 3	–	143701° 3
872.802	5	114573.52	−.001	32398 4	–	146972° 3	997.661	10	100234.44	−.007	14357 2	–	114591° 3
875.221	10	114256.85	.000	32398 4	–	146655° 5	997.845	10	100215.96	−.007	16282 4	–	116497° 3
877.678	2	113937.00	.004	32398 4	–	146336° 4	998.423	10bl	100157.94	−.007	44655 4	–	144812° 3
880.639	1	113553.90	−.006	32398 4	–	145951° 4	998.596	5	100140.59	−.005	43561 3	–	143701° 3
881.902	1	113391.28	.000	32587 3	–	145978° 2	998.646	30	100135.58	−.008	13948 3	–	114083° 2
885.640	1	112912.69	.007	51425 3	–	164339° 3	999.132	30	100086.87	−.008	13928 2	–	114014° 1
886.377	1	112818.81	.004	19973 6	–	132792° 5	999.760	1	100024.00	.000	43561 3	–	143585° 2
886.839	2	112760.03	.002	32587 3	–	145347° 2	1000.570	1	99943.03	−.004	242 1	–	100184° 1
888.982	10	112488.21	.001	32587 3	–	145075° 4	1000.765	5	99923.55	−.007	15871 3	–	115794° 2
898.355	5	111314.56	−.001	32387 2	–	143701° 3	1001.021	1	99898.00	−.007	51482 2	–	151380° 1
907.659	1h	110173.53	−.009	36164 4	–	146336° 4	1002.575	1	99743.16	−.007	43461 3	–	143204° 2
917.386	1	109005.37	.005	13811 6	–	122817° 5	1002.652	20	99735.50	−.006	46601 4	–	146336° 4
957.280	2	104462.64	.001	34225 4	–	138688° 4	1002.725	1bl	99728.24	.010	668 2	–	100397° 3
960.056	5	104160.59	.000	54853 5	–	159013° 5	1002.749	5bl	99725.85	−.004	14357 2	–	114083° 2
967.020	1	103410.47	.001	43561 3	–	146972° 3	1003.073	2	99693.64	.000	46962 5	–	146655° 5
969.021	2	103196.93	−.001	16282 4	–	119479° 3	1003.375	3	99663.63	−.001	19896 1	–	119559° 0
971.091	5	102976.96	−.001	52697 4	–	155674° 4	1003.592	5	99642.08	.002	43561 3	–	143204° 2
972.047	2h	102875.68	−.008	43461 3	–	146336° 4	1003.716	10	99629.77	−.002	19576 2	–	119206° 1
973.001	2	102774.81	−.001	43561 3	–	146336° 4	1004.054	5	99596.23	.000	32843 2	–	132439° 2
973.299	5	102743.35	−.004	11271 0	–	114014° 1	1004.086	10	99593.06	.006	19576 2	–	119170° 2
975.131	1	102550.32	−.006	13948 3	–	116497° 3				.000	34225 4	–	133818° 3
976.402	5	102416.83	.001	43561 3	–	145978° 2	1006.311	40	99372.85	−.002	35129 5	–	134502° 4
977.354	30	102317.07	−.001	44655 4	–	146972° 3	1006.403	5	99363.77	.000	32418 1	–	131782° 1
977.605	5	102290.80	.006	49088 2	–	151380° 1	1006.534	1	99350.84	−.004	46601 4	–	145951° 4
			−.001	42521 2	–	144812° 3	1006.942	2	99310.58	−.004	19896 1	–	119206° 1
979.041	1	102140.76	−.004	14357 2	–	116497° 3	1010.231	10	98987.26	.000	33452 3	–	132439° 2
980.387	10	102000.53	−.001	44655 4	–	146655° 5	1010.474	20	98963.45	−.001	19487 3	–	118451° 2
981.131	20	101923.18	.008	23183 2	–	125107° 1	1010.716	20	98939.76	−.006	32843 2	–	131782° 1
981.240	1	101911.86	−.003	12679 4	–	114591° 3	1011.384	10	98874.41	.002	19576 2	–	118451° 2
981.489	5	101886.01	.000	43461 3	–	145347° 2	1011.453	20	98867.66	.000	20611 4	–	119479° 3
981.685	1	101865.67	−.003	13928 2	–	115794° 2	1014.515	2	98569.26	−.001	52811 1	–	151380° 1
982.455	20	101785.83	.000	43561 3	–	145347° 2	1015.093	5	98513.14	−.002	46299 3	–	144812° 3
983.466	30	101681.19	.000	44655 4	–	146336° 4	1017.205	2	98308.60	.000	16282 4	–	114591° 3
984.511	10	101573.26	−.004	12510 1	–	114083° 2	1018.210	5	98211.56	.003	15871 3	–	114083° 2
985.087	2	101513.87	.001	43561 3	–	145075° 4				−.007	46601 4	–	144812° 3
985.175	3	101504.80	−.003	12510 1	–	114014° 1	1019.226	10	98113.66	−.001	46962 5	–	145075° 4
985.549	1	101466.28	−.007	15871 3	–	117336° 2	1020.693	1	97972.65	.002	53407 0	–	151380° 1
985.741	1	101446.52	−.003	35129 5	–	136575° 4	1025.535	1	97510.08	−.004	46299 3	–	143809° 3
986.669	5	101351.11	−.002	43461 3	–	144812° 3	1027.894	2	97286.29	−.001	46299 3	–	143585° 2
987.200	20	101296.59	.001	44655 4	–	145951° 4	1028.923	5	97189.00	.000	54191 2	–	151380° 1
987.646	30	101250.85	−.001	43561 3	–	144812° 3	1029.861	5	97100.48	−.002	46601 4	–	143701° 3
987.718	1	101243.47	−.003	33452 3	–	134695° 2	1031.760	1	96921.76	−.005	19576 2	–	116497° 3
989.471	10	101064.10	−.001	42521 2	–	143585° 2	1031.945	2	96904.38	.001	46299 3	–	143204° 2
989.720	2	101038.67	−.009	23183 2	–	124221° 1	1032.068	1	96892.84	.008	242 1	–	97135° 0
990.042	1	101005.81	−.007	16282 4	–	117287° 4	1034.688	2	96647.49	−.006	32418 1	–	129065° 1
990.880	5	100920.39	−.004	42665 2	–	143585° 2	1035.439	1	96577.39	−.002	42665 2	–	139243° 3
991.239	1	100883.84	−.005	46299 3	–	147182° 2	1036.053	1	96520.15	.008	44655 4	–	141176° 4
991.852	20	100821.49	.000	54853 5	–	155674° 4	1036.136	2	96512.42	.000	33452 3	–	129964° 3
993.224	20	100682.22	.000	42521 2	–	143204° 2	1036.336	40bl	96493.80	.007	242 1	–	96736° 1
993.766	2	100627.31	−.006	15871 3	–	116497° 3	1036.893	1	96441.96	−.007	35129 5	–	131570° 4
994.294	20	100573.87	−.004	16714 5	–	117287° 4	1038.020	2	96337.25	−.004	50318 5	–	146655° 5
994.645	5	100538.38	−.001	42665 2	–	143204° 2	1038.861	1	96259.26	−.004	49088 2	–	145347° 2
995.810	10	100420.76	−.003	44655 4	–	145075° 4	1039.253	1	96222.96	−.007	32843 2	–	129065° 1
996.300	5	100371.37	−.006	46601 4	–	146972° 3	1039.370	5	96212.12	−.002	32843 2	–	129055° 2
996.348	1	100366.53	−.004	33452 3	–	133818° 3	1039.782	30	96174.00	.001	50481 6	–	146655° 5
996.767	1	100324.34	−.003	42521 2	–	142845° 1	1041.475	10	96017.66	.000	50318 5	–	146336° 4
1041.892	1	95979.23	−.006	46962 5	–	142940° 6	1080.333	20	92564.05	.001	32418 1	–	124982° 0
1042.230	1	95948.11	.000	49088 2	–	145036° 1				−.004	13948 3	–	106511° 4
1042.903	5	95886.19	−.001	20611 4	–	116497° 3	1080.449	1	92554.11	−.002	668 2	–	93222° 1
1044.503	5	95739.31	−.001	34225 4	–	129964° 3	1080.893	20	92516.09	.001	32843 2	–	125359° 2
1044.668	1	95724.19	−.005	49088 2	–	144812° 3	1081.061	5	92501.71	.004	43461 3	–	135963° 2
			−.008	47978 2	–	143701° 3	1081.401	20	92472.63	.003	27006 3	–	119479° 3
1045.134	2	95681.51	−.003	43561 3	–	139243° 3	1081.499	1	92464.25	−.009	32519 1	–	124982° 0
1045.992	5	95603.02	−.001	33452 3	–	129055° 2	1081.563	40	92458.78	.005	50481 6	–	142940° 6
1046.607	2	95546.84	−.005	51425 3	–	146972° 3	1081.777	1h	92440.49	−.004	42404 1	–	134844° 1
1046.742	1	95534.52	−.005	49541 4	–	145075° 4	1082.230	2	92401.80	.002	43561 3	–	135963° 2
1048.247	1	95397.36	−.004	32398 4	–	127795° 3	1083.020	10	92334.39	.002	33452 3	–	125786° 3
1049.208	2	95309.98	−.002	242 1	–	95551° 2	1083.332	30	92307.80	.004	34225 4	–	126533° 4
1049.596	1	95274.75	−.001	42521 2	–	137796° 3	1083.524	1	92291.44	−.009	42404 1	–	134695° 2
1049.640	5	95270.75	.001	49541 4	–	144812° 3	1083.843	40	92264.28	.001	32843 2	–	125107° 1
1051.487	5	95103.41	.000	19487 3	–	114591° 3	1084.297	1	92225.65	.001	1872 4	–	94098° 4
1052.576	1	95005.01	.003	46962 5	–	141967° 5				.001	52811 1	–	145036° 1
1053.547	1	94917.45	−.001	32418 1	–	127336° 2	1084.848	10	92178.81	.004	42665 2	–	134844° 1
1055.333	20	94756.82	.001	50318 5	–	145075° 4	1086.945	1	92000.97	−.001	12510 1	–	104511° 2
1056.252	1	94674.37	−.002	50362 1	–	145036° 1	1087.898	10	91920.38	.000	44655 4	–	136575° 4
1056.654	1	94638.35	−.001	56741 2	–	151380° 1	1088.057	30	91906.95	.003	33452 3	–	125359° 2
1057.614	2	94552.45	.004	51425 3	–	145978° 2	1089.473	1	91787.49	.000	54191 2	–	145978° 2
1057.909	2	94526.08	.000	51425 3	–	145951° 4	1090.207	20	91725.70	.004	46962 5	–	138688° 4
1058.127	5	94506.61	−.002	19576 2	–	114083° 2	1091.124	30	91648.61	.000	50318 5	–	141967° 5
1058.249	2	94495.71	.001	51482 2	–	145978° 2	1091.295	20	91634.25	.003	49541 4	–	141176° 4
1059.955	5	94343.62	.000	33452 3	–	127795° 3	1091.350	2	91629.63	−.003	53407 0	–	145036° 1
1060.958	10	94254.43	−.001	35129 5	–	129383° 4	1091.392	2	91626.10	.001	44655 4	–	136281° 3
1061.713	1	94187.41	−.004	19896 1	–	114083° 2	1092.164	40	91561.34	.001	34225 4	–	125786° 3
1062.022	20	94160.00	.003	49541 4	–	143701° 3	1092.934	10	91496.83	.001	46299 3	–	137796° 3
1062.100	2	94153.09	.000	23183 2	–	117336° 2	1093.549	1	91445.37	−.009	27006 3	–	118451° 2
1062.484	2	94119.06	−.004	19896 1	–	114014° 1	1093.719	2	91431.16	−.001	668 2	–	92099° 2
1064.714	1	93921.93	−.003	51425 3	–	145347° 2	1094.043	40	91404.08	−.001	35129 5	–	126533° 4
1065.146	2	93883.84	.000	33452 3	–	127336° 2	1094.297	2	91382.87	−.001	46962 5	–	138344° 5
1065.178	2	93881.02	−.004	42521 2	–	136402° 3	1095.218	1	91306.02	−.006	46299 3	–	137605° 4
1065.732	5	93832.22	.000	12679 4	–	106511° 4	1095.328	1	91296.85	−.003	42521 2	–	133818° 3
1066.816	5	93736.87	−.002	42665 2	–	136402° 3	1096.082	5	91234.05	−.003	43461 3	–	134695° 2
1067.984	1	93634.36	.001	42665 2	–	136300° 1	1096.551	5	91195.02	−.001	46601 4	–	137796° 3
1068.715	20	93570.31	.002	34225 4	–	127795° 3	1097.016	2	91156.37	.000	54191 2	–	145347° 2
1068.847	10	93558.76	.003	42404 1	–	135963° 2	1097.060	2	91152.71	−.001	42665 2	–	133818° 3
1068.901	1	93554.03	−.001	51482 2	–	145036° 1	1097.290	5	91133.61	.000	43561 3	–	134695° 2
1069.949	5	93462.39	−.001	56741 2	–	150204° 1	1097.539	1	91112.93	−.005	13928 2	–	105041° 1
1070.060	2	93452.70	.001	1223 3	–	94676° 3	1098.414	10	91040.35	.002	43461 3	–	134502° 4
1072.544	5	93236.26	.002	13275 5	–	106511° 4	1098.860	20	91003.40	.006	52697 4	–	143701° 3
1072.703	1	93222.44	−.001	0 0	–	93222° 1				.001	46601 4	–	137605° 4
1072.967	10	93199.51	.005	42521 2	–	135721° 2	1099.051	2	90987.58	.001	12679 4	–	103667° 3
			−.001	32587 3	–	125786° 3	1099.623	5	90940.25	.001	43561 3	–	134502° 4
1073.333	2	93167.73	−.001	52811 1	–	145978° 2	1099.889	20	90918.26	.000	34225 4	–	125143° 5
1074.620	1	93056.15	−.001	42665 2	–	135721° 2	1100.100	1	90900.82	−.006	42521 2	–	133422° 2
1075.498	1	92980.18	.002	242 1	–	93222° 1	1100.279	10	90886.03	.001	1872 4	–	92758° 3
1075.955	30	92940.69	.002	43461 3	–	136402° 3	1100.409	10	90875.30	.003	1223 3	–	92099° 2
			.001	32418 1	–	125359° 2	1100.628	10	90857.22	.002	50318 5	–	141176° 4
1077.112	5	92840.85	−.001	43561 3	–	136402° 3	1102.125	1	90733.81	−.003	42521 2	–	133255° 3
			−.009	32519 1	–	125359° 2	1103.230	5	90642.93	.001	46962 5	–	137605° 4
			.007	50362 1	–	143204° 2	1103.497	1	90620.99	.003	54191 2	–	144812° 3
1077.361	1	92819.39	.006	43461 3	–	136281° 3	1103.787	5	90597.18	−.002	12679 4	–	103276° 4
1077.537	2	92804.23	.000	1872 4	–	94676° 3	1103.877	10	90589.80	−.002	42665 2	–	133255° 3
1077.807	2	92780.99	.001	54191 2	–	146972° 3	1103.961	1	90582.91	−.006	13928 2	–	104511° 2
1078.520	5	92719.65	.007	32387 2	–	125107° 1	1104.201	2	90563.22	−.005	13948 3	–	104511° 2
			.001	43561 3	–	136281° 3	1104.320	30	90553.46	.001	33452 3	–	124005° 4
1078.875	10	92689.14	−.002	32418 1	–	125107° 1	1104.710	1	90521.49	−.007	16282 4	–	106803° 3
1079.659	1	92621.83	.002	50318 5	–	142940° 6	1105.579	1	90450.34	.003	1223 3	–	91674° 2
1106.720	1	90357.09	−.004	43461 3	–	133818° 3	1131.614	40	88369.35	−.001	50318 5	–	138688° 4
1106.857	20	90345.90	.000	13275 5	–	103621° 5	1131.861	5	88350.07	−.001	242 1	–	88592° 2
1107.045	1	90330.56	−.005	27006 3	–	117336° 2	1132.760	1	88279.95	−.001	1223 3	–	89503° 4
1107.243	1	90314.41	−.003	668 2	–	90982° 2	1133.083	30	88254.78	.001	49541 4	–	137796° 3
1108.287	2	90229.33	−.001	16282 4	–	106511° 4	1134.054	20	88179.22	−.003	12679 4	–	100858° 4
1109.087	30	90164.25	.000	32843 2	–	123007° 3	1134.583	20	88138.10	.002	58730 4	–	146868° 3
			−.009	1223 3	–	91387° 4				−.007	44655 4	–	132792° 5
1109.208	10	90154.41	−.002	49088 2	–	139243° 3	1134.773	20	88123.35	−.001	59059 2	–	147182° 2
1110.375	5	90059.66	.002	242 1	–	90301° 1	1136.241	2	88009.49	−.006	43561 3	–	131570° 4
1110.682	10	90034.77	.000	42404 1	–	132439° 2	1136.688	30	87974.88	.000	58893 3	–	146868° 3
			.001	52811 1	–	142845° 1	1137.236	1	87932.49	−.006	42521 2	–	130453° 3
1110.934	300	90014.34	−.002	35129 5	–	125143° 5	1137.350	10	87923.68	−.001	668 2	–	88592° 2
1111.097	100	90001.14	.001	13275 5	–	103276° 4	1137.872	30	87883.34	−.003	31323 2	–	119206° 1
1111.335	10	89981.86	.001	46299 3	–	136281° 3	1137.966	20	87876.08	.002	1223 3	–	89100° 4
			−.009	55366 1	–	145347° 2	1138.133	80	87863.19	.001	50481 6	–	138344° 5
1111.429	10	89974.25	−.002	46601 4	–	136575° 4	1138.728	20	87817.28	−.002	51425 3	–	139243° 3
1111.595	1	89960.82	−.003	43461 3	–	133422° 2				.002	1872 4	–	89689° 5
1112.101	1	89919.89	.002	668 2	–	90588° 3	1138.833	1	87809.18	−.001	59059 2	–	146868° 3
1112.126	2	89917.86	−.004	668 2	–	90586° 2	1138.997	2	87796.54	−.004	15871 3	–	103667° 3
			−.002	42521 2	–	132439° 2	1140.013	40	87718.29	−.004	12679 4	–	100397° 3
1112.621	40	89877.86	.001	12679 4	–	102557° 3						–	
					–		1140.408	5	87687.91	−.002	35129 5	–	122817° 5
1113.006	5h	89846.77	.001	44655 4	–	134502° 4	1140.652	40bl	87669.15	−.002	42404 1	–	130073° 2
1113.060	1h	89842.41	−.004	32387 2	–	122229° 2	1140.691	60	87666.16	.000	33452 3	–	121118° 4
1113.460	200	89810.14	.001	13811 6	–	103621° 5				−.005	32398 4	–	120064° 3
			.009	32418 1	–	122229° 2	1141.139	1	87631.74	−.005	1872 4	–	89503° 4
1113.654	1h	89794.49	−.009	43461 3	–	133255° 3	1141.694	10	87589.14	.000	242 1	–	87831° 1
1113.828	200	89780.46	−.001	34225 4	–	124005° 4	1141.765	50h	87583.69	−.007	13275 5	–	100858° 4
1113.908	20	89774.02	−.004	42665 2	–	132439° 2	1142.178	10	87552.02	−.002	42521 2	–	130073° 2
1114.085	1h	89759.75	.002	42404 1	–	132164° 1	1142.333	20	87540.14	−.002	46962 5	–	134502° 4
1114.588	5	89719.25	−.001	13948 3	–	103667° 3	1142.607	5	87519.15	−.004	46299 3	–	133818° 3
1115.078	40	89679.82	.001	46601 4	–	136281° 3						–	
					–		1143.567	5	87445.68	−.001	48854 0	–	136300° 1
1115.536	1h	89643.00	−.003	42521 2	–	132164° 1	1143.733	50	87432.99	−.003	15871 3	–	103303° 2
1115.657	2	89633.28	.001	668 2	–	90301° 1	1144.058	50	87408.15	−.003	42665 2	–	130073° 2
1115.847	5	89618.02	.000	54191 2	–	143809° 3	1144.362	20	87384.93	−.003	16282 4	–	103667° 3
1115.903	30	89613.52	.001	46962 5	–	136575° 4	1144.584	20	87367.98	.002	1223 3	–	88592° 2
1116.630	50	89555.17	.002	33452 3	–	123007° 3	1144.963	5bl	87339.06	−.005	16282 4	–	103621° 5
1117.331	5	89498.99	−.002	42665 2	–	132164° 1	1145.489	2	87298.96	−.001	42665 2	–	129964° 3
1117.426	10	89491.38	−.001	27006 3	–	116497° 3	1145.652	60	87286.54	−.003	50318 5	–	137605° 4
1117.615	40	89476.25	.000	35129 5	–	124605° 6	1145.968	2	8726247	−.004	51425 3	–	138688° 4
1118.291	40	89422.16	.001	46299 3	–	135721° 2	1146.379	10	87231.18	.001	242 1	–	87473° 2
1118.739	50	89386.35	−.001	32843 2	–	122229° 2						–	
					–		1146.424	50	87227.76	−.001	1872 4	–	89100° 4
1118.848	10	89377.64	.002	42404 1	–	131782° 1	1146.507	40	87221.44	.001	58730 4	–	145951° 4
1119.044	5	89361.99	.001	1223 3	–	90586° 2				.000	32843 2	–	120064° 3
			.000	59059 2	–	148421° 2	1146.637	10	87211.55	−.001	49088 2	–	136300° 1
1119.118	1	89356.08	−.005	13948 3	–	103303° 2	1147.278	40	87162.83	−.001	668 2	–	87831° 1
1119.460	2	89328.78	−.004	13948 3	–	103276° 4	1147.549	2	87142.24	.000	668 2	–	87810° 3
1119.755	50	89305.25	−.002	32418 1	–	121723° 1	1147.732	40	87128.35	−.003	31323 2	–	118451° 2
1120.308	1	89261.16	−.006	42521 2	–	131782° 1	1147.805	5	87122.81	−.003	46299 3	–	133422° 2
1121.359	5	89177.50	.004	1872 4	–	91050° 3	1148.358	5	87080.85	−.003	32398 4	–	119479° 3
1121.751	10	89146.34	.001	49541 4	–	138688° 4	1148.656	2	87058.26	−.001	58893 3	–	145951° 4
1121.838	10	89139.43	.005	0 0	–	89139° 1						–	
					–		1148.892	20	87040.38	−.002	32519 1	–	119559° 0
1124.689	1	88913.46	−.008	11271 0	–	100184° 1	1148.973	10	87034.24	−.003	49541 4	–	136575° 4
1124.889	10	88897.66	.001	242 1	–	89139° 1	1150.005	5	86956.14	−.004	46299 3	–	133255° 3
1125.107	20	88880.43	.001	32843 2	–	121723° 1	1150.607	30	86910.64	−.003	13948 3	–	100858° 4
1125.156	10	88876.56	−.003	35129 5	–	124005° 4	1150.651	60	86907.32	−.004	16714 5	–	103621° 5
1126.350	20	88782.35	−.002	34225 4	–	123007° 3	1150.851	30	86892.22	−.001	32587 3	–	119479° 3
1126.412	20	88777.46	−.002	33452 3	–	122229° 2				−.001	43561 3	–	130453° 3
1128.292	2	88629.53	−.007	13928 2	–	102557° 3	1151.080	10	86874.93	.000	49088 2	–	135963° 2
1128.774	50	88591.69	.002	34225 4	–	- 122817° 5	1151.822	5	86818.97	−.003	32387 2	–	119206° 1
1130.217	10	88478.58	−.004	52697 4	–	141176° 4	1152.011	40	86804.72	.002	668 2	–	87473° 2
1130.308	5	88471.46	−.001	668 2	–	89139° 1						–	
1152.716	40	86751.63	.000	32418 1	–	119170° 2	1169.319	500	85519.86	−.008	1872 4	–	87391° 3
1152.871	1	86739.97	−.002	49541 4	–	136281° 3	1169.678	10	85493.61	−.002	43561 3	–	129055° 2
1153.093	40	86723.27	.001	668 2	–	87391° 3	1170.074	5	85464.68	.000	11271 0	–	96736° 1
1153.170	1	86717.48	−.007	47978 2	–	134695° 2				−.001	19576 2	–	105041° 1
1153.577	40	86686.88	.000	32519 1	–	119206° 1	1170.400	200	85440.87	.003	242 1	–	85683° 1
			−.006	15871 3	–	102557° 3	1171.175	10	85384.33	.000	13928 2	–	99313° 1
1154.016	5	86653.91	−.001	46601 4	–	133255° 3	1173.669	500	85202.89	−.001	1223 3	–	86426° 2
1154.376	2	86626.88	−.002	1872 4	–	88499° 3	1174.399	5	85149.93	−.002	51425 3	–	136575° 4
1154.573	40	86612.10	.000	43461 3	–	130073° 2	1174.463	5	85145.29	.000	19896 1	–	105041° 1
			.005	33452 3	–	120064° 3	1174.675	2	85129.93	.002	42665 2	–	127795° 3
1154.915	40	86586.45	.003	1223 3	–	87810° 3	1175.109	20	85098.48	.001	52697 4	–	137796° 3
1155.233	200	86562.62	−.004	16714 5	–	103276° 4	1175.382	30	85078.72	.001	58730 4	–	143809° 3
1155.483	10bl	86543.89	−.001	42521 2	–	129065° 1	1175.551	30	85066.49	.006	242 1	–	85308° 1
1155.624	20	86533.33	.000	42521 2	–	129055° 2	1176.148	5	85023.31	−.001	19487 3	–	104511° 2
1155.912	20	86511.77	.002	43561 3	–	130073° 2	1176.194	1	85019.98	.002	1872 4	–	86892° 5
1156.028	2	86503.09	−.001	43461 3	–	129964° 3	1176.273	200	85014.27	.005	668 2	–	85683° 1
1156.483	20	86469.06	−.001	13928 2	–	100397° 3	1176.644	1	84987.47	.000	15871 3	–	100858° 4
1156.742	80	86449.70	−.004	13948 3	–	100397° 3	1176.791	10	84976.85	−.004	51425 3	–	136402° 3
1157.370	20	86402.79	.001	43561 3	–	129964° 3	1176.899	80	84969.05	.002	46601 4	–	131570° 4
1157.404	1	86400.25	−.005	42665 2	–	129065° 1	1177.173	20	84949.28	.000	32387 2	–	117336° 2
1158.390	2	86326.71	.004	32843 2	–	119170° 2	1177.378	20	84934.49	.000	19576 2	–	104511° 2
1158.439	10bl	86323.06	−.003	54853 5	–	141176° 4	1177.759	30	84907.01	.002	52697 4	–	137605° 4
1158.863	30	86291.47	−.003	13928 2	–	100219° 2	1178.012	200	84888.77	.002	32398 4	–	117287° 4
1159.082	50	86275.17	−.003	16282 4	–	102557° 3	1179.006	10	84817.21	.003	32519 1	–	117336° 2
1159.126	20	86271.89	−.003	13948 3	–	100219° 2				.002	51482 2	–	136300° 1
1159.336	30	86256.27	−.004	13928 2	–	100184° 1	1179.045	10	84814.40	−.003	42521 2	–	127336° 2
			.008	50318 5	–	136575° 4	1179.262	5	84798.79	−.002	51482 2	–	136281° 3
1159.431	200	86249.20	.002	1223 3	–	87473° 2	1179.948	10	84749.49	.002	59059 2	–	143809° 3
1160.206	100	86191.59	.002	20611 4	–	106803° 3				−.002	32587 3	–	117336° 2
			−.004	46601 4	–	132792° 5	1180.237	50	84728.74	−.003	44655 4	–	129383° 4
1160.302	100	86184.45	.004	242 1	–	86426° 2	1180.631	20	84700.46	−.001	32587 3	–	117287° 4
1160.528	500	86167.67	.002	1223 3	–	87391° 3	1180.749	20	84692.00	.000	58893 3	–	143585° 2
1160.906	1	86139.61	.004	46299 3	–	132439° 2	1180.805	10	84687.98	−.002	48734 1	–	133422° 2
1161.302	20	86110.24	.004	48734 1	–	134844° 1	1181.050	5	84670.42	−.002	42665 2	–	127336° 2
1161.689	1	86081.55	.006	58730 4	–	144812° 3	1181.175	10	84661.46	.001	668 2	–	85329° 2
1161.703	5	86080.52	.001	242 1	–	86322° 0	1181.468	10	84640.46	.000	668 2	–	85308° 1
1161.925	2	86064.07	−.004	32387 2	–	118451° 2	1181.558	2	84634.01	−.005	13928 2	–	98562° 3
1162.105	30	86050.74	.001	33155 0	–	119206° 1	1181.831	10	84614.46	−.005	13948 3	–	98562° 3
1162.479	5	86023.05	.007	13928 2	–	99952° 2				.009	19896 1	–	104511° 2
1162.736	10	86004.04	−.001	13948 3	–	99952° 2	1181.911	40	84608.74	−.001	46962 5	–	131570° 4
1162.925	10	85990.06	.004	48854 0	–	134844° 1	1182.364	20	84576.32	−.005	16282 4	–	100858° 4
			.001	52697 4	–	138688° 4	1182.902	10	84537.85	−.001	51425 3	–	135963° 2
1163.318	20	85961.01	.001	48734 1	–	134695° 2	1183.059	20	84526.63	−.002	15871 3	–	100397° 3
1163.630	500	85937.97	.003	1872 4	–	87810° 3				−.006	59059 2	–	143585° 2
			−.005	50362 1	–	136300° 1	1183.357	20	84505.35	−.004	12679 4	–	97184° 4
1163.711	50	85931.98	−.001	32519 1	–	118451° 2	1183.676	1	84482.57	−.004	50362 1	–	134844° 1
1163.893	10	85918.55	.002	58893 3	–	144812° 3	1183.836	1	84471.16	−.003	31323 2	–	115794° 2
1164.144	20	85900.02	.001	20611 4	–	106511° 4	1183.976	1	84461.17	−.002	47978 2	–	132439° 2
1164.376	40	85882.91	−.003	12679 4	–	98562° 3	1184.359	20	84433.85	−.004	13275 5	–	97709° 5
1164.634	20	85863.88	.000	32587 3	–	118451° 2	1185.552	200	84348.89	−.002	15871 3	–	100219° 2
1165.083	100	85830.79	−.001	46962 5	–	132792° 5	1185.757	5h	84334.31	−.001	43461 3	–	127795° 3
1165.131	5	85827.25	−.002	14357 2	–	100184° 1	1186.093	20	84310.42	−.003	58893 3	–	143204° 2
1165.200	10	85822.17	−.002	43561 3	–	129383° 4	1186.565	10	84276.88	−.002	49541 4	–	133818° 3
1165.520	5	85798.61	.000	44655 4	–	130453° 3	1187.163	5	84234.43	−.004	43561 3	–	127795° 3
1166.067	300	85758.36	.000	668 2	–	86426° 2	1187.575	5	84205.20	−.005	14357 2	–	98562° 3
1167.093	100	85682.97	.002	0 0	–	85683° 1	1187.841	1	84186.35	−.003	47978 2	–	132164° 1
1168.126	30	85607.20	−.007	49088 2	–	134695° 2	1187.934	20	84179.76	−.004	19487 3	–	103667° 3
1168.200	1	85601.78	−.009	50362 1	–	135963° 2	1188.113	1	84167.07	−.006	49088 2	–	133255° 3
1168.305	5	85594.08	.008	14357 2	–	99952° 2	1188.230	200	84158.79	−.002	12679 4	–	96838° 3
			−.007	43461 3	–	129055° 2	1188.289	50	84154.61	−.004	46299 3	–	130453° 3
1188.430	200	84144.62	−.003	59059 2	–	143204° 2	1208.313	20	82760.01	−.001	31323 2	–	114083° 2
			−.005	16714 5	–	100858° 4	1208.379	1	82755.49	.002	46299 3	–	129055° 2
1188.846	200	84115.18	−.003	16282 4	–	100397° 3	1208.435	50	82751.65	.001	54853 5	–	137605° 4
1188.909	20	84110.72	−.003	32387 2	–	116497° 3	1209.281	30	82693.76	.000	49088 2	–	131782° 1
1188.974	40	84106.12	−.001	1223 3	–	85329° 2				−.002	42665 2	–	125359° 2
1189.070	50	84099.33	−.004	32398 4	–	116497° 3	1209.317	30	82691.30	−.002	15871 3	–	98562° 3
1189.192	20	84090.71	.000	19576 2	–	103667° 3				.005	31323 2	–	114014° 1
			−.004	64331 3	–	148421° 2	1209.602	200	82671.82	.003	1872 4	–	84544° 4
1189.325	30	84081.30	−.003	15871 3	–	99952° 2	1209.704	10	82664.85	.000	20611 4	–	103276° 4
1190.839	5	83974.40	−.001	242 1	–	84216° 0	1209.756	20	82661.29	−.002	13928 2	–	96589° 2
1191.760	300	83909.51	−.004	13275 5	–	97184° 4	1210.042	50	82641.76	−.002	13948 3	–	96589° 2
1191.922	100	83898.10	−.003	13811 6	–	97709° 5	1210.974	20	82578.15	−.001	42404 1	–	124982° 0
1192.208	20	83877.98	−.003	52697 4	–	136575° 4	1212.032	40	82506.07	.000	12510 1	–	95016° 1
1192.571	30	83852.45	−.002	46601 4	–	130453° 3	1212.405	20	82480.68	.001	14357 2	–	96838° 3
1192.819	30	83835.01	−.004	54853 5	–	138688° 4	1212.506	300	82473.81	.003	50318 5	–	132792° 5
			.008	33452 3	–	117287° 4				.005	13811 6	–	96285° 5
1193.082	10	83816.53	−.006	19487 3	–	103303° 2	1212.976	10	82441.86	.001	42665 2	–	125107° 1
1193.471	20	83789.21	−.005	19487 3	–	103276° 4	1213.037	20	82437.71	−.001	11271 0	–	93709° 1
1193.512	20	83786.33	−.001	59059 2	–	142845° 1	1213.273	20	82421.68	.001	46962 5	–	129383° 4
1194.347	10	83727.76	−.006	19576 2	–	103303° 2	1213.705	10	82392.34	.002	51425 3	–	133818° 3
1195.241	5	83665.13	−.001	46299 3	–	129964° 3	1214.177	20	82360.31	.004	1223 3	–	83584° 3
1195.483	20	83648.19	−.005	19973 6	–	103621° 5	1214.545	5	82335.36	.003	51482 2	–	133818° 3
1195.705	20	83632.66	−.004	13275 5	–	96907° 6	1214.695	20	82325.19	.000	43461 3	–	125786° 3
1195.883	10	83620.22	−.004	23183 2	–	106803° 3	1214.902	50	82311.16	.001	50481 6	–	132792° 5
1197.760	2	83489.18	−.001	52811 1	–	136300° 1	1215.369	10	82279.53	.002	16282 4	–	98562° 3
1198.603	20	83430.46	−.003	48734 1	–	132164° 1	1215.422	10	82275.95	.003	12679 4	–	94955° 4
1198.943	50	83406.80	−.003	32387 2	–	115794° 2	1216.067	5	82232.31	.000	14357 2	–	96589° 2
1199.587	10	83362.02	.000	51482 2	–	134844° 1	1216.176	20	82224.94	.001	43561 3	–	125786° 3
1199.747	10	83350.90	−.002	49088 2	–	132439° 2	1216.493	10	82203.51	.004	32387 2	–	114591° 3
1200.186	30	83320.41	.002	1223 3	–	84544° 4	1216.665	200	82191.89	.007	32398 4	–	114591° 3
1200.258	100	83315.42	.001	36164 4	–	119479° 3	1217.901	10	82108.48	.007	54191 2	–	136300° 1
1200.841	2	83274.97	−.004	32519 1	–	115794° 2	1218.371	1	82076.80	.002	50362 1	–	132439° 2
1200.919	20	83269.56	−.001	51425 3	–	134695° 2	1219.083	10	82028.86	.004	49541 4	–	131570° 4
1200.982	20	83265.19	−.003	42521 2	–	125786° 3	1219.459	100	82003.57	.005	32587 3	–	114591° 3
1201.182	1	83251.33	−.002	49541 4	–	132792° 5	1219.569	10h	81996.18	.002	51425 3	–	133422° 2
1201.389	2	83236.98	−.001	58730 4	–	141967° 5	1220.248	20	81950.55	.000	11271 0	–	93222° 1
			−.007	13948 3	–	97184° 4	1220.321	2	81945.65	.002	20611 4	–	102557° 3
1201.826	40	83206.72	−.001	32587 3	–	115794° 2	1220.590	1	81927.59	.002	13928 2	–	95856° 3
1202.259	100	83176.75	.000	12679 4	–	95856° 3	1220.879	100	81908.19	.000	13948 3	–	95856° 3
1202.781	5	83140.65	−.001	44655 4	–	127795° 3	1221.037	1	81897.60	.003	43461 3	–	125359° 2
1203.061	80	83121.30	−.004	42665 2	–	125786° 3	1221.239	1	81884.05	.004	52811 1	–	134695° 2
1203.416	100	83096.78	−.001	13811 6	–	96907° 6	1221.333	5	81877.75	−.004	12510 1	–	94387° 2
1203.718	40	83075.93	−.001	49088 2	–	132164° 1				.007	44655 4	–	126533° 4
			.004	51425 3	–	134502° 4	1221.427	5	81871.45	.004	668 2	–	82540° 2
1203.776	100	83071.93	−.001	43461 3	–	126533° 4	1222.467	5	81801.79	.003	50362 1	–	132164° 1
1204.019	5	83055.16	.005	20611 4	–	103667° 3	1222.526	5	81797.85	.003	43561 3	–	125359° 2
1204.124	5	83047.92	.004	48734 1	–	131782° 1	1222.758	60	81782.33	.001	12510 1	–	94292° 1
1204.215	30	83041.65	−.001	12510 1	–	95551° 2	1223.659	50	81722.11	.004	54853 5	–	136575° 4
1204.513	5	83021.10	−.004	11271 0	–	94292° 1	1223.814	30	81711.76	.004	1872 4	–	83584° 3
1204.676	30	83009.87	.000	13275 5	–	96285° 5	1224.058	60	81695.47	.002	32387 2	–	114083° 2
			−.005	20611 4	–	103621° 5						–	
					–		1224.284	60	81680.39	−.001	13275 5	–	94955° 4
1205.094	10	82981.07	−.001	19576 2	–	102557° 3	1224.783	1	81647.11	.006	64331 3	–	145978° 2
1205.228	20	82971.85	−.002	43561 3	–	126533° 4	1225.083	200	81627.12	.003	32387 2	–	114014° 1
1205.477	20	82954.71	.000	42404 1	–	125359° 2	1225.143	100	81623.12	.000	13928 2	–	95551° 2
1205.536	1	82950.65	.001	32843 2	–	115794° 2	1225.436	300	81603.60	−.001	13948 3	–	95551° 2
1206.040	5	82915.99	.001	668 2	–	83584° 3	1227.011	100	81498.86	.000	14357 2	–	95856° 3
1206.131	20	82909.73	−.001	13928 2	–	96838° 3	1227.061	100	81495.54	.002	32587 3	–	114083° 2
1206.979	10	82851.48	.004	64331 3	–	147182° 2				−.002	32519 1	–	114014° 1
1207.181	20	82837.61	.000	42521 2	–	125359° 2	1227.933	5	81437.66	.003	12679 4	–	94117° 3
1207.611	1	82808.12	−.005	13928 2	–	96736° 1				−.003	53407 0	–	134844° 1
1207.989	10	82782.21	.000	46601 4	–	129383° 4						–	
1228.101	40	81426.52	−.002	16282 4	–	97709° 5	1246.157	5	80246.71	.001	20611 4	–	100858° 4
1228.226	200	81418.24	.005	12679 4	–	94098° 4	1246.814	40	80204.42	.001	12679 4	–	92884° 5
1228.943	100	81370.73	.001	19487 3	–	100858° 4	1247.059	1	80188.66	.003	13928 2	–	94117° 3
1229.028	1	81365.11	.001	49088 2	–	130453° 3	1247.662	30	80149.91	.001	13948 3	–	94098° 4
1229.807	30	81313.57	.000	15871 3	–	97184° 4	1247.743	10	80144.70	.003	51425 3	–	131570° 4
1230.390	300	81275.04	.003	1872 4	–	83147° 5	1247.861	200	80137.13	.004	1872 4	–	82009° 4
1230.743	30	81251.73	.004	50318 5	–	131570° 4	1248.127	300	80120.05	.004	23183 2	–	103303° 2
1230.929	10	81239.45	.005	32843 2	–	114083° 2	1248.771	200	80078.73	.003	12679 4	–	92758° 3
1231.540	10	81199.14	.001	12510 1	–	93709° 1	1249.124	10	80056.10	.002	19896 1	–	99952° 2
1231.614	80	81194.27	.000	46601 4	–	127795° 3	1249.526	200	80030.34	.004	13275 5	–	93306° 6
			.000	14357 2	–	95551° 2				−.003	14357 2	–	94387° 2
1231.962	5	81171.33	.002	32843 2	–	114014° 1	1249.958	50	80002.68	.000	16282 4	–	96285° 5
1232.689	20	81123.46	.005	36164 4	–	117287° 4	1250.232	60	79985.15	.001	15871 3	–	95856° 3
1232.740	30	81120.10	.005	52697 4	–	133818° 3	1250.360	10h	79976.96	−.001	49088 2	–	129065° 1
1233.234	300	81087.61	.000	13928 2	–	95016° 1	1250.525	50	79966.41	.001	49088 2	–	129055° 2
			−.007	47978 2	–	129065° 1				−.004	62879 0	–	142845° 1
1233.403	5	81076.50	.003	47978 2	–	129055° 2	1250.665	10	79957.46	.003	58730 4	–	138688° 4
1234.015	40	81036.29	.002	46299 3	–	127336° 2	1251.014	40	79935.15	−.001	14357 2	–	94292° 1
1234.321	5	81016.20	.003	64331 3	–	145347° 2	1252.475	1	79841.91	.004	49541 4	–	129383° 4
1234.648	100	80994.74	−.001	16714 5	–	97709° 5	1252.716	200	79826.55	−.001	13275 5	–	93102° 4
1235.071	200	80967.00	.002	15871 3	–	96838° 3	1252.873	80	79816.55	.003	1223 3	–	81040° 3
1235.910	40	80912.04	.003	49541 4	–	130453° 3	1253.181	60	79796.93	.001	27006 3	–	106803° 3
1235.941	30	80910.01	−.004	19487 3	–	100397° 3	1253.221	10bl	79794.38	−.002	58893 3	–	138688° 4
1236.065	300	80901.89	.003	16282 4	–	97184° 4	1253.355	40	79785.85	−.001	20611 4	–	100397° 3
1236.465	30	80875.72	.003	49088 2	–	129964° 3	1253.432	80	79780.95	−.003	13928 2	–	93709° 1
1237.278	30	80822.58	.003	13275 5	–	94098° 4	1253.759	60	79760.14	−.002	14357 2	–	94117° 3
1237.301	100	80821.07	−.001	19576 2	–	100397° 3	1254.133	40	79736.36	.000	19576 2	–	99313° 1
1237.846	100	80785.49	.007	1223 3	–	82009° 4	1254.485	30	79713.98	−.003	13928 2	–	93642° 2
1238.424	1	80747.78	.004	13928 2	–	94676° 3	1254.586	1h	79707.56	.002	42521 2	–	122229° 2
			.005	72356 2	–	153104° 3	1254.794	40	79694.35	−.002	13948 3	–	93642° 2
1238.669	1	80731.81	.004	19487 3	–	100219° 2	1254.925	300	79686.03	.000	668 2	–	80354° 3
1238.720	20	80728.49	−.001	13948 3	–	94676° 3	1255.010	80	79680.64	−.001	15871 3	–	95551° 2
1238.868	30	80718.85	−.002	15871 3	–	96589° 2	1255.517	2	79648.46	.005	54853 5	–	134502° 4
1238.973	60	80712.00	.002	12510 1	–	93222° 1	1255.836	5	79628.23	.003	52811 1	–	132439° 2
1239.442	1	80681.46	.004	51482 2	–	132164° 1	1256.144	300	79608.70	−.001	13275 5	–	92884° 5
1239.787	10	80659.01	−.004	14357 2	–	95016° 1	1256.451	100	79589.25	.001	12510 1	–	92099° 2
1240.029	20	80643.27	.001	19576 2	–	100219° 2	1256.681	100	79574.68	.002	12679 4	–	92254° 5
1240.226	1	80630.46	.006	33452 3	–	114083° 2	1256.742	500	79570.82	.003	16714 5	–	96285° 5
1240.501	200	80612.59	.004	13811 6	–	94424° 7	1256.856	2	79563.61	.003	42665 2	–	122229° 2
1240.574	1	80607.84	.002	19576 2	–	100184° 1	1257.141	1h	79545.57	.006	43461 3	–	123007° 3
1241.355	20bl	80557.13	.005	52697 4	–	133255° 3	1257.784	2	79504.90	.007	27006 3	–	106511° 4
1241.377	100	80555.70	−.001	16282 4	–	96838° 3	1257.946	200	79494.66	.004	13811 6	–	93306° 6
1241.557	5	80544.02	.002	43461 3	–	124005° 4	1258.600	100	79453.36	.001	13275 5	–	92728° 6
1242.046	30	80512.31	.003	58730 4	–	139243° 3	1259.176	40	79417.01	.000	19896 1	–	99313° 1
1242.490	10	80483.54	.002	23183 2	–	103667° 3	1259.862	1	79373.77	.003	23183 2	–	102557° 3
1242.695	200	80470.26	.002	16714 5	–	97184° 4	1260.185	2	79353.42	.002	52811 1	–	132164° 1
1242.787	10	80464.31	.001	19487 3	–	99952° 2	1260.209	2	79351.91	.000	14357 2	–	93709° 1
1242.868	20	80459.06	−.001	13928 2	–	94387° 2	1260.575	5	79328.87	.001	55366 1	–	134695° 2
1243.173	60	80439.32	.002	13948 3	–	94387° 2	1261.133	20	79293.77	−.002	13928 2	–	93222° 1
1243.436	300	80422.31	.000	12679 4	–	93102° 4	1262.215	500	79225.80	.001	242 1	–	79467° 2
1244.168	20	80374.99	.009	19576 2	–	99952° 2	1262.861	2	79185.27	−.001	46601 4	–	125786° 3
1244.213	60	80372.09	.002	668 2	–	81040° 3	1263.135	20	79168.10	.002	1872 4	–	81040° 3
1244.339	60	80363.95	.000	13928 2	–	94292° 1	1263.202	1	79163.90	.007	12510 1	–	91674° 2
1244.569	5	80349.10	.001	58893 3	–	139243° 3	1263.737	300	79130.38	.002	1223 3	–	80354° 3
1244.684	5	80341.67	.002	42665 2	–	123007° 3	1264.465	20	79084.83	−.003	15871 3	–	94955° 4
1244.809	20	80333.60	.005	36164 4	–	116497° 3	1264.653	200	79073.07	−.001	13811 6	–	92884° 5
1244.845	40	80331.28	.000	48734 1	–	129065° 1	1265.339	50	79030.20	−.003	11271 0	–	90301° 1
1244.960	60	80323.86	.002	19896 1	–	100219° 2	1266.047	40	78986.00	−.005	19576 2	–	98562° 3
1245.012	20	80320.51	.006	48734 1	–	129055° 2	1266.159	500	78979.02	.000	13275 5	–	92254° 5
1245.033	40	80319.15	.000	14357 2	–	94676° 3	1266.286	20	78971.10	−.001	51482 2	–	130453° 3
1245.508	5	80288.52	.002	19896 1	–	100184° 1				.005	52811 1	–	131782° 1
1266.664	300	78947.53	.004	0 0	–	78947° 1	1286.568	300	77726.16	.002	13948 3	–	91674° 2
1267.139	800	78917.94	−.003	13811 6	–	92728° 6	1286.897	1	77706.29	−.001	46299 3	–	124005° 4
1267.383	1	78902.74	−.001	58893 3	–	137796° 3	1287.053	20	77696.87	.000	19487 3	–	97184° 4
1267.839	1	78874.36	.004	58730 4	–	137605° 4	1287.305	2	77681.66	.004	58893 3	–	136575° 4
1267.887	20	78871.38	.004	1223 3	–	80095° 4	1287.474	2h	77671.47	.009	58730 4	–	136402° 3
1267.995	200	78864.66	.002	14357 2	–	93222° 1	1287.718	2h	77656.75	.000	43461 3	–	121118° 4
1268.393	20	78839.91	.000	668 2	–	79508° 3	1287.938	1h	77643.48	.002	46962 5	–	124605° 6
1268.551	20	78830.09	−.003	13928 2	–	92758° 3	1288.069	300	77635.59	.004	1872 4	–	79508° 3
1268.867	200	78810.46	−.002	13948 3	–	92758° 3	1288.179	20bl	77628.96	.006	51425 3	–	129055° 2
1268.943	80	78805.74	−.003	15871 3	–	94676° 3	1288.257	300	77624.26	.006	1872 4	–	79497° 4
1269.047	200	78799.28	.003	668 2	–	79467° 2	1288.950	2	77582.52	.004	51482 2	–	129065° 1
1269.490	10	78771.79	.002	242 1	–	79013° 2	1289.074	200	77575.06	.005	12679 4	–	90255° 4
1270.051	20	78736.99	−.002	59059 2	–	137796° 3	1289.118	20bl	77572.41	−.001	51482 2	–	129055° 2
1270.521	500	78707.86	−.001	12679 4	–	91387° 4	1290.253	10	77504.17	.006	27006 3	–	104511° 2
1270.762	60	78692.94	−.005	50362 1	–	129055° 2	1290.495	500	77489.64	.006	668 2	–	78158° 3
1271.007	50	78677.77	.003	0 0	–	78677° 1	1291.337	10	77439.11	.002	13948 3	–	91387° 4
1271.079	100	78673.31	−.003	16282 4	–	94955° 4	1292.198	20	77387.52	.003	58893 3	–	136281° 3
1271.492	1	78647.76	.001	51425 3	–	130073° 2	1292.307	10	77380.99	−.001	47978 2	–	125359° 2
1272.237	40	78601.70	.000	48734 1	–	127336° 2	1292.818	20	77350.40	.001	19487 3	–	96838° 3
1273.260	40	78538.55	.003	51425 3	–	129964° 3	1293.379	80	77316.85	.002	14357 2	–	91674° 2
1274.181	300	78481.78	.000	51482 2	–	129964° 3	1293.755	100	77294.38	.002	22890 0	–	100184° 1
			.003	1872 4	–	80354° 3	1294.241	100	77265.36	.004	12510 1	–	89775° 0
1274.363	800	78470.57	.002	1872 4	–	80343° 5	1294.677	100	77239.34	.004	19896 1	–	97135° 0
1274.805	100	78443.37	−.001	13811 6	–	92254° 5	1294.821	20	77230.75	.001	15871 3	–	93102° 4
1274.932	500	78435.55	.006	242 1	–	78677° 1	1295.105	30	77213.81	.003	23183 2	–	100397° 3
1275.070	60	78427.06	.003	36164 4	–	114591° 3	1295.410	800	77195.63	.001	13811 6	–	91006° 5
1275.491	30	78401.18	−.002	14357 2	–	92758° 3	1296.016	200	77159.54	.004	19576 2	–	96736° 1
1275.608	200	78393.98	.001	16282 4	–	94676° 3	1296.654	100	77121.57	.000	13928 2	–	91050° 3
1275.987	20	78370.70	−.002	12679 4	–	91050° 3	1296.981	500	77102.13	−.002	13948 3	–	91050° 3
1276.397	300	78345.53	.000	668 2	–	79013° 2				−.002	19487 3	–	96589° 2
1276.712	300	78326.20	.002	242 1	–	78568° 0	1297.416	30	77076.28	.004	56741 2	–	133818° 3
1277.395	500	78284.32	.001	1223 3	–	79508° 3	1297.476	2	77072.71	.005	55366 1	–	132439° 2
1277.479	100	78279.17	.002	668 2	–	78947° 1	1297.793	1	77053.89	.000	13928 2	–	90982° 2
1277.582	50bl	78272.86	.005	1223 3	–	79497° 4	1297.968	1	77043.50	.003	46962 5	–	124005° 4
1277.885	1h	78254.30	−.002	49541 4	–	127795° 3	1298.093	30	77036.08	.003	23183 2	–	100219° 2
1278.016	100bl	78246.28	.002	15871 3	–	94117° 3	1298.120	20	77034.48	−.003	13948 3	–	90982° 2
1278.398	800	78222.90	.004	1872 4	–	80095° 4	1298.482	100	77013.00	.004	19576 2	–	96589° 2
1279.075	1	78181.49	.001	46962 5	–	125143° 5	1298.531	60	77010.09	.002	12679 4	–	89689° 5
1279.246	80	78171.04	−.003	13928 2	–	92099° 2	1298.688	100	77000.78	.003	23183 2	–	100184° 1
1279.569	80	78151.31	−.001	13948 3	–	92099° 2	1299.050	300	76979.33	.004	13275 5	–	90255° 4
1280.217	200	78111.75	.004	13275 5	–	91387° 4	1299.153	30	76973.22	.004	50362 1	–	127336° 2
1280.806	50	78075.83	−.001	12510 1	–	90586° 2	1299.812	800	76934.20	.004	1223 3	–	78158° 3
1281.139	50	78055.54	.003	55366 1	–	133422° 2				.003	19973 6	–	96907° 6
1281.899	300	78009.26	.004	668 2	–	78677° 1	1300.604	30	76887.35	.001	15871 3	–	92758° 3
1282.491	20	77973.25	.002	54191 2	–	132164° 1	1301.405	200	76840.03	.007	19896 1	–	96736° 1
1282.743	100	77957.93	.000	51425 3	–	129383° 4	1301.677	500	76823.97	.003	12679 4	–	89503° 4
1282.861	500	77950.76	−.004	20611 4	–	98562° 3	1301.754	20	76819.43	−.002	16282 4	–	93102° 4
1283.049	2h	77939.34	.003	54853 5	–	132792° 5	1302.619	50	76768.41	.002	23183 2	–	99952° 2
1283.553	300	77908.74	.000	12679 4	–	90588° 3	1303.886	50	76693.82	.001	19896 1	–	96589° 2
1284.226	200	77867.91	.002	11271 0	–	89139° 1	1304.020	1h	76685.94	−.001	52697 4	–	129383° 4
1284.605	10	77844.94	.005	58730 4	–	136575° 4	1304.118	50	76680.17	.002	56741 2	–	133422° 2
1284.773	500	77834.76	.002	16282 4	–	94117° 3	1304.424	20bl	76662.19	.000	59059 2	–	135721° 2
1285.092	50	77815.44	.002	16282 4	–	94098° 4	1304.502	500	76657.60	−.005	13928 2	–	90586° 2
1285.209	1	77808.35	.000	47978 2	–	125786° 3	1304.798	200	76640.21	.000	13948 3	–	90588° 3
1285.509	500	77790.19	−.003	1223 3	–	79013° 2	1304.980	200	76629.52	.001	12510 1	–	89139° 1
1285.821	500	77771.32	.000	15871 3	–	93642° 2	1305.056	300	76625.06	.000	48734 1	–	125359° 2
1286.245	100	77745.68	.003	13928 2	–	91674° 2				.000	14357 2	–	90982° 2
1286.307	100	77741.93	.001	14357 2	–	92099° 2	1305.456	100	76601.58	−.002	16282 4	–	92884° 5
1286.410	500	77735.71	−.003	19973 6	–	- 97709° 5	1305.576	500	76594.54	.004	242 1	–	76836° 2
1286.482	100	77731.36	.000	13275 5	–	91006° 5	1305.622	200	76591.84	−.002	16714 5	–	93306° 6
1305.946	80	76572.84	.001	20611 4	–	97184° 4	1325.564	100	75439.58	.001	19576 2	–	95016° 1
1306.174	40	76559.47	−.001	11271 0	–	87831° 1	1326.635	20	75378.68	.000	23183 2	–	98562° 3
1306.497	10	76540.55	−.002	42665 2	–	119206° 1	1327.653	30	75320.88	.001	12510 1	–	87831° 1
1306.957	30	76513.61	−.002	56741 2	–	133255° 3	1327.958	30	75303.58	.006	668 2	–	75972° 1
1307.601	200	76475.92	.000	16282 4	–	92758° 3	1329.001	80	75244.48	.002	20611 4	–	95856° 3
1308.509	50	76422.86	.001	22890 0	–	99313° 1	1329.073	100	75240.41	.007	1872 4	–	77113° 5
1308.554	20	76420.23	.004	12679 4	–	89100° 4	1329.587	200	75211.32	−.004	13928 2	–	89139° 1
1308.654	30	76414.39	.000	13275 5	–	89689° 5	1329.984	300	75188.87	−.001	19487 3	–	94676° 3
1309.112	2	76387.65	.000	16714 5	–	93102° 4	1330.160	100	75178.92	.003	15871 3	–	91050° 3
1309.362	300	76373.07	.001	13928 2	–	90301° 1	1330.387	300	75166.09	.001	13275 5	–	88441° 6
			.005	48734 1	–	125107° 1						–	
					–		1330.639	5	75151.86	.001	13948 3	–	89100° 4
1309.439	100	76368.58	.000	19487 3	–	95856° 3	1330.974	20	75132.94	−.004	49088 2	–	124221° 1
1310.408	500	76312.11	−.005	19973 6	–	96285° 5	1331.012	60	75130.80	.003	12679 4	–	87810° 3
1310.500	100	76306.75	.000	13948 3	–	90255° 4	1331.198	20	75120.30	.000	19896 1	–	95016° 1
1310.666	300	76297.08	.005	27006 3	–	103303° 2	1331.471	40	75104.90	−.002	16282 4	–	91387° 4
1310.864	200	76285.56	.006	1872 4	–	78158° 3	1331.555	40	75100.16	−.002	19576 2	–	94676° 3
1311.122	2	76270.55	.002	49088 2	–	125359° 2	1333.990	20	74963.08	.000	12510 1	–	87473° 2
			−.007	27006 3	–	103276° 4	1334.679	10	74924.38	.004	58893 3	–	133818° 3
1311.260	2	76262.52	.002	54191 2	–	130453° 3	1335.119	200	74899.69	.002	19487 3	–	94387° 2
1311.397	10	76254.55	.000	52811 1	–	129065° 1	1335.304	1	74889.31	.003	43561 3	–	118451° 2
1311.506	1	76248.22	.004	48734 1	–	124982° 0						–	
					–		1335.884	1h	74856.79	.003	89482 3	–	164339° 3
1311.595	80	76243.04	−.001	47978 2	–	124221° 1	1336.431	10	74826.16	.005	72356 2	–	147182° 2
1311.758	10	76233.57	.002	72187 3	–	148421° 2	1336.636	5	74814.68	.004	42521 2	–	117336° 2
1311.846	200	76228.46	.000	14357 2	–	90586° 2	1336.703	200	74810.93	.002	19576 2	–	94387° 2
			−.002	15871 3	–	92099° 2	1337.219	200	74782.06	.003	14357 2	–	89139° 1
			−.002	13275 5	–	89503° 4	1337.479	30	74767.52	.001	16282 4	–	91050° 3
1311.879	100	76226.54	−.001	20611 4	–	96838° 3	1338.263	1	74723.72	.008	16282 4	–	91006° 5
1312.854	100	76169.93	−.002	16714 5	–	92884° 5	1338.392	500	74716.52	−.009	19576 2	–	94292° 1
1313.031	200	76159.66	−.003	12510 1	–	88669° 0	1338.471	10	74712.11	.000	12679 4	–	87391° 3
1313.556	100	76129.22	.002	23183 2	–	99313° 1	1338.671	20	74700.95	.002	72481 1	–	147182° 2
1314.373	60	76081.90	−.001	12510 1	–	88592° 2						–	
					–		1339.166	30	74673.34	−.004	16714 5	–	91387° 4
1314.684	5	76063.90	.000	19487 3	–	95551° 2	1339.344	300	74663.41	−.001	13928 2	–	88592° 2
1315.538	1	76014.52	.001	16714 5	–	92728° 6	1339.696	300	74643.80	.000	13948 3	–	88592° 2
1316.220	100	75975.14	.000	19576 2	–	95551° 2	1339.944	500	74629.98	.008	13811 6	–	88441° 6
1316.283	200	75971.50	.005	16282 4	–	92254° 5				−.005	19487 3	–	94117° 3
1316.756	100	75944.21	.001	14357 2	–	90301° 1	1340.199	2	74615.78	.001	72356 2	–	146972° 3
1316.834	5	75939.71	.001	72481 1	–	148421° 2	1340.299	10	74610.21	.003	19487 3	–	94098° 4
1317.347	5	75910.14	.000	51425 3	–	127336° 2	1340.619	100	74592.40	.003	1223 3	–	75816° 4
1317.894	500	75878.63	.001	13811 6	–	89689° 5	1341.012	200	74570.54	−.001	13928 2	–	88499° 3
1318.340	10	75852.96	.003	51482 2	–	127336° 2	1341.362	300	74551.09	−.003	13948 3	–	88499° 3
1318.835	80	75824.49	.003	13275 5	–	89100° 4						–	
					–		1341.549	200	74540.69	.004	19576 2	–	94117° 3
1318.919	500	75819.66	−.003	12679 4	–	88499° 3	1341.776	2	74528.08	.005	58893 3	–	133422° 2
1319.203	10	75803.34	.000	15871 3	–	91674° 2	1341.833	1	74524.92	.001	52811 1	–	127336° 2
1319.228	2bl	75801.90	−.006	58893 3	–	134695° 2				.000	58730 4	–	133255° 3
1319.513	5	75785.53	−.001	42665 2	–	118451° 2	1342.066	20	74511.98	.004	72356 2	–	146868° 3
			−.004	59059 2	–	134844° 1	1342.432	80	74491.66	.001	19896 1	–	94387° 2
1319.758	20	75771.46	.003	58730 4	–	134502° 4	1343.176	10	74450.40	.002	19973 6	–	94424° 7
1320.482	5	75729.92	.007	242 1	–	75972° 1	1343.797	5	74416.00	.003	32387 2	–	106803° 3
1321.045	10Hbl	75697.64	−.001	56741 2	–	132439° 2	1343.879	30	74411.46	−.003	11271 0	–	85683° 1
1321.467	20	75673.47	.001	14296 4	–	75816° 4	1344.005	10	74404.48	.005	32398 4	–	106803° 3
			.001	20611 4	–	96285° 5						–	
					–		1344.146	200	74396.68	.000	19896 1	–	94292° 1
1321.734	10	75658.18	.001	53407 0	–	129065° 1	1344.377	40	74383.89	−.001	15871 3	–	90255° 4
1321.779	60	75655.61	.003	19896 1	–	95551° 2	1344.769	1bl	74362.21	.007	59059 2	–	133422° 2
1322.533	100	75612.48	.007	1223 3	–	76836° 2	1344.793	10	74360.88	.000	51425 3	–	125786° 3
1323.529	200	75555.57	.000	13948 3	–	89503° 4	1345.100	2	74343.91	.001	20611 4	–	94955° 4
1323.609	40	75551.01	.001	27006 3	–	102557° 3	1345.790	10	74305.79	−.001	16282 4	–	90588° 3
1323.800	200	75540.11	.000	16714 5	–	92254° 5	1346.030	200	74292.54	−.001	16714 5	–	91006° 5
1324.217	40	75516.32	.000	15871 3	–	91387° 4	1347.084	50	74234.42	.002	14357 2	–	88592° 2
1324.730	2	75487.08	.001	48734 1	–	124221° 1	1347.418	10	74216.01	.004	32587 3	–	106803° 3
1324.848	20	75480.35	.003	31323 2	–	106803° 3	1347.480	200	74212.60	.006	12679 4	–	86892° 5
1325.064	100	75468.05	−.002	19487 3	–	94955° 4						–	
1347.791	1	74195.48	.006	59059 2	–	133255° 3	1369.222	20	7303417	.001	14357 2	–	87391° 3
1348.648	30	74148.33	.003	72187 3	–	146336° 4	1370.034	5	72990.88	.005	72356 2	–	145347° 2
1348.769	20	74141.68	−.001	14357 2	–	88499° 3	1370.323	100	72975.49	.001	16714 5	–	89689° 5
1348.930	300	74132.83	−.001	19576 2	–	93709° 1	1370.885	300	72945.57	.001	27006 3	–	99952° 2
1349.088	1	74124.14	−.001	50481 6	–	124605° 6	1371.541	20	72910.69	−.001	19973 6	–	92884° 5
1349.294	60	74112.83	.004	32398 4	–	106511° 4	1371.975	1	72887.62	.003	72187 3	–	145075° 4
1350.152	50	74065.73	.002	19576 2	–	93642° 2	1372.158	1	72877.90	.001	46601 4	–	119479° 3
1350.213	30	74062.38	.000	58730 4	–	132792° 5	1372.297	1	72870.52	.004	46299 3	–	119170° 2
1350.329	30	74056.02	.005	668 2	–	74724° 3	1372.325	2	72869.03	.004	48854 0	–	121723° 1
1350.673	40	74037.16	.000	11271 0	–	85308° 1	1372.382	1	72866.01	−.005	72481 1	–	145347° 2
1351.860	300	73972.15	.004	16282 4	–	90255° 4	1372.644	2	72852.10	.002	1872 4	–	74724° 3
1352.378	40	73943.82	.004	1872 4	–	75816° 4	1372.866	1	72840.32	.000	58730 4	–	131570° 4
1352.877	40	73916.55	.001	12510 1	–	86426° 2	1373.253	5	72819.79	−.001	12510 1	–	85329° 2
1353.510	5	73881.98	.000	13928 2	–	87810° 3	1373.298	2	72817.40	.001	16282 4	–	89100° 4
1353.608	1	73876.63	−.002	51482 2	–	125359° 2	1373.644	1h	72799.06	−.007	12510 1	–	85308° 1
1354.058	30	73852.08	.000	27006 3	–	100858° 4	1373.825	200	72789.47	.000	16714 5	–	89503° 4
1354.164	80	73846.29	.001	22890 0	–	96736° 1	1374.470	100	72755.31	.002	19973 6	–	92728° 6
1354.536	2	73826.01	.003	43461 3	–	117287° 4	1375.125	40	72720.66	.004	15871 3	–	88592° 2
1354.600	1	73822.53	.005	77557 2	–	151380° 1	1375.895	20	72679.96	.007	72356 2	–	145036° 1
1354.784	20	73812.50	−.001	12510 1	–	86322° 0	1375.955	20	72676.79	.003	58893 3	–	131570° 4
1355.189	20	73790.44	.006	72187 3	–	145978° 2	1376.523	40	72646.80	−.001	77557 2	–	150204° 1
1355.681	20bl	73763.66	.007	72187 3	–	145951° 4	1376.786	1	72632.92	−.008	44655 4	–	117287° 4
1355.994	200	73746.63	−.002	19896 1	–	93642° 2	1376.882	80	72627.86	.003	15871 3	–	88499° 3
1356.521	20	73717.98	.003	31323 2	–	105041° 1	1377.017	200	72620.74	−.001	13275 5	–	85896° 4
1356.632	1h	73711.95	.001	56741 2	–	130453° 3	1378.268	5	72554.83	.002	72481 1	–	145036° 1
1357.688	40	73654.62	.000	23183 2	–	96838° 3	1378.883	200D	72522.46	.008	19576 2	–	92099° 2
1357.854	10	73645.62	.002	19576 2	–	93222° 1	1379.496	500	72490.24	−.002	20611 4	–	93102° 4
1358.091	10	73632.76	−.002	15871 3	–	89503° 4	1379.714	1h	72478.78	−.005	13948 3	–	86426° 2
1358.379	200	73617.15	−.001	13275 5	–	86892° 5	1380.152	2	72455.78	.005	72356 2	–	144812° 3
1358.433	60	73614.23	−.002	19487 3	–	93102° 4	1381.488	50	72385.71	.001	16714 5	–	89100° 4
1358.490	2	73611.14	.000	242 1	–	73853° 2	1381.825	50	72368.06	.001	23183 2	–	95551° 2
1358.606	1	73604.85	−.004	54191 2	–	127795° 3	1382.279	500	72344.29	.000	31323 2	–	103667° 3
1359.571	50	73552.61	.003	23183 2	–	96736° 1	1382.875	5	72313.11	.004	56741 2	–	129055° 2
1359.718	40	73544.66	−.001	13928 2	–	87473° 2	1383.491	20	72280.91	.001	19973 6	–	92254° 5
1359.788	1	73540.87	−.004	16714 5	–	90255° 4	1383.660	100	72272.09	.004	20611 4	–	92884° 5
1360.440	30	73505.63	.001	20611 4	–	94117° 3	1384.178	1	72245.04	−.010	64331 3	–	136575° 4
1360.536	20	73500.44	.006	1223 3	–	74724° 3	1384.427	1	72232.05	.004	43561 3	–	115794° 2
1360.797	500	73486.34	.001	20611 4	–	94098° 4	1384.725	30	72216.50	.000	16282 4	–	88499° 3
1361.186	1	73465.34	.000	64331 3	–	137796° 3	1385.082	10	72197.89	.002	32843 2	–	105041° 1
1361.414	20	73453.04	.001	14357 2	–	87810° 3	1385.300	80	72186.53	.002	19487 3	–	91674° 2
1362.263	200	73407.26	−.001	16282 4	–	89689° 5	1386.067	10	72146.58	.003	20611 4	–	92758° 3
1362.281	200	73406.29	−.002	23183 2	–	96589° 2	1386.463	80	72125.97	.004	22890 0	–	95016° 1
1362.565	20	73390.99	.001	27006 3	–	100397° 3	1386.510	30	72123.53	.003	32387 2	–	104511° 2
1362.768	1	73380.06	−.003	59059 2	–	132439° 2	1387.004	30	72097.84	.001	19576 2	–	91674° 2
1363.651	40	73332.54	.000	19973 6	–	93306° 6	1387.113	10	72092.18	.005	32418 1	–	104511° 2
1363.769	10	73326.20	.003	19896 1	–	93222° 1	1387.553	50	72069.31	.002	42521 2	–	114591° 3
1364.746	5bl	73273.70	.004	64331 3	–	137605° 4				−.001	14357 2	–	86426° 2
1364.800	200	73270.80	−.002	19487 3	–	92758° 3	1389.049	100	71991.70	.001	32519 1	–	104511° 2
1365.577	1	73229.11	−.003	15871 3	–	89100° 4	1389.892	80	71948.03	−.002	13948 3	–	85896° 4
1365.725	40	73221.18	−.001	16282 4	–	89503° 4	1390.365	80	71923.56	.004	32587 3	–	104511° 2
1365.813	60	73216.46	.001	12679 4	–	85896° 4	1390.827	100	71899.66	−.001	19487 3	–	91387° 4
1365.873	60	73213.24	.002	27006 3	–	100219° 2	1391.095	100	71885.81	.001	33155 0	–	105041° 1
1366.347	100	73187.85	.003	31323 2	–	104511° 2	1391.501	30	71864.84	.000	12679 4	–	84544° 4
1366.410	10	73184.47	.005	668 2	–	73853° 2	1391.933	5	71842.53	.003	44655 4	–	116497° 3
1366.457	50	73181.95	.000	19576 2	–	92758° 3	1392.125	5	71832.63	.000	23183 2	–	95016° 1
1366.627	50	73172.85	.000	12510 1	–	85683° 1	1393.177	20	71778.38	.003	19896 1	–	91674° 2
1366.876	10	73159.52	.003	72187 3	–	145347° 2	1393.644	10	71754.33	.001	13928 2	–	85683° 1
1367.694	2	73115.77	−.001	14357 2	–	87473° 2	1394.165	300	71727.52	−.005	16714 5	–	88441° 6
1368.337	500	73081.41	.000	13811 6	–	86892° 5	1394.580	30	71706.17	.000	12510 1	–	84216° 0
1369.175	1	73036.68	−.007	43461 3	–	116497° 3	1395.327	1	71667.78	.001	32843 2	–	104511° 2
1395.818	20	71642.57	.001	20611 4	–	92254° 5	1415.360	50	70653.40	−.001	58730 4	–	129383° 4
1396.234	20	71621.23	.002	72187 3	–	143809° 3	1415.639	500	70639.47	.002	36164 4	–	106803° 3
1396.607	10	71602.10	.000	15871 3	–	87473° 2	1415.998	5	70621.56	−.003	43461 3	–	114083° 2
1397.381	20	71562.44	−.001	19487 3	–	91050° 3	1416.225	500	70610.24	−.007	16282 4	–	86892° 5
1397.427	5	71560.08	.000	58893 3	–	130453° 3	1416.504	60	70596.34	−.002	13948 3	–	84544° 4
1397.511	80	71555.78	.000	27006 3	–	98562° 3	1416.548	10	70594.14	.000	56741 2	–	127336° 2
1398.581	5	71501.04	.000	47978 2	–	119479° 3	1417.319	1	70555.74	−.003	15871 3	–	86426° 2
1398.703	80	71494.80	−.002	19487 3	–	90982° 2	1417.919	300	70525.89	−.003	23183 2	–	93709° 1
1398.736	40	71493.12	−.002	23183 2	–	94676° 3				.007	75816° 4	–	146342 5
			−.004	42521 2	–	114014° 1	1418.012	10	70521.26	.001	43561 3	–	114083° 2
1399.118	50	71473.60	.001	19576 2	–	91050° 3	1418.657	2	70489.20	.005	72356 2	–	142845° 1
1399.524	5	71452.86	−.001	72356 2	–	143809° 3	1418.990	2	70472.66	.002	47978 2	–	118451° 2
1400.442	20	71406.02	−.002	19576 2	–	90982° 2	1418.998	1	70472.26	−.008	48734 1	–	119206° 1
1400.513	50	71402.40	.003	22890 0	–	94292° 1	1419.081	100	70468.14	−.002	12679 4	–	83147° 5
1400.672	10	71394.30	.000	59059 2	–	130453° 3	1419.681	20	70438.35	.001	20611 4	–	91050° 3
1400.925	300	71381.40	.006	13948 3	–	85329° 2	1419.729	2	70435.97	.000	48734 1	–	119170° 2
1401.560	5	71349.06	−.002	42665 2	–	114014° 1	1420.339	30	70405.72	.002	19896 1	–	90301° 1
1402.023	20	71325.50	.001	14357 2	–	85683° 1	1420.554	30	70395.07	−.001	20611 4	–	91006° 5
1402.921	20	71279.85	.002	32387 2	–	103667° 3	1421.956	5	70325.66	.001	46962 5	–	117287° 4
1403.146	500	71268.42	.002	32398 4	–	103667° 3	1424.940	40	70178.39	−.003	16714 5	–	86892° 5
					–					−.005	27006 3	–	97184° 4
1403.817	80	71234.35	.004	31323 2	–	102557° 3						–	
			−.004	58730 4	–	129964° 3	1425.109	60	70170.07	.003	32387 2	–	102557° 3
1403.919	5	71229.18	.003	72356 2	–	143585° 2	1425.285	10	70161.40	−.001	58893 3	–	129055° 2
1403.946	2	71227.81	−.001	47978 2	–	119206° 1	1425.338	10	70158.79	.000	32398 4	–	102557° 3
1404.050	200	71222.53	.000	32398 4	–	103621° 5	1426.179	1	70117.42	.001	49088 2	–	119206° 1
1404.414	30	71204.07	−.001	23183 2	–	94387° 2	1427.784	30	70038.60	.001	23183 2	–	93222° 1
1405.890	1	71129.32	.006	43461 3	–	114591° 3	1428.059	300	70025.11	−.002	15871 3	–	85896° 4
1406.293	100	71108.93	.003	16282 4	–	87391° 3	1428.241	200	70016.19	−.005	19487 3	–	89503° 4
			.000	23183 2	–	94292° 1	1428.446	5	70006.14	−.002	59059 2	–	129065° 1
1406.453	5	71100.84	−.005	19487 3	–	90588° 3	1429.049	50	69976.60	.000	20611 4	–	90588° 3
					–		1429.180	100	69970.19	.002	32587 3	–	102557° 3
1406.501	30	71098.42	−.006	19487 3	–	90586° 2						–	
1406.731	10	71086.79	−.004	19896 1	–	90982° 2	1429.880	20	69935.93	.001	44655 4	–	114591° 3
1406.865	2	71080.02	.000	32587 3	–	103667° 3	1430.690	1	69896.34	.001	46601 4	–	116497° 3
1407.283	2	71058.91	.000	33452 3	–	104511° 2	1431.027	80	69879.88	−.002	19896 1	–	89775° 0
1407.380	1	71054.01	−.001	56741 2	–	127795° 3	1431.602	20	69851.81	−.001	33452 3	–	103303° 2
1407.716	1	71037.05	.002	46299 3	–	117336° 2	1432.014	100	69831.72	.000	27006 3	–	96838° 3
1407.784	1	71033.62	−.005	19973 6	–	91006° 5	1432.165	1	69824.35	.003	33452 3	–	103276° 4
1407.868	1	71029.38	.002	43561 3	–	114591° 3	1434.382	30	69716.43	−.001	19973 6	–	89689° 5
1408.213	300	71011.98	−.004	19576 2	–	90588° 3	1434.422	1	69714.49	−.002	32843 2	–	102557° 3
1408.266	300	71009.31	.001	19576 2	–	90586° 2	1435.629	80	69655.87	−.001	13928 2	–	83584° 3
					–		1435.893	200	69643.07	.002	20611 4	–	90255° 4
1408.998	80	70972.42	.000	14357 2	–	85329° 2						–	
1409.420	40	70951.17	.004	14357 2	–	85308° 1	1436.032	100	69636.33	−.001	13948 3	–	83584° 3
1409.762	40	70933.95	−.002	23183 2	–	94117° 3	1436.252	5	69625.66	−.004	77557 2	–	147182° 2
1410.109	10	70916.50	.001	32387 2	–	103303° 2	1436.499	20	69613.69	−.004	16282 4	–	85896° 4
			−.001	54191 2	–	125107° 1	1436.523	10	69612.53	−.005	19487 3	–	89100° 4
1410.343	80	70904.73	.002	12679 4	–	83584° 3	1437.301	40	69574.84	.001	23183 2	–	92758° 3
			.006	59059 2	–	129964° 3	1438.963	5	69494.49	−.001	46299 3	–	115794° 2
1410.727	1	70885.43	−.003	32418 1	–	103303° 2	1439.113	30	69487.24	.000	64331 3	–	133818° 3
1410.880	80	70877.75	.002	32398 4	–	103276° 4	1439.700	5	69458.91	−.003	15871 3	–	85329° 2
1411.153	30	70864.03	.003	77557 2	–	148421° 2	1441.700	30	69362.55	−.001	49088 2	–	118451° 2
					–		1441.791	200	69358.18	.002	47978 2	–	117336° 2
1411.486	5	70847.32	.005	72356 2	–	143204° 2						–	
1411.929	1	70825.09	.003	48734 1	–	119559° 0	1442.243	500	69336.44	.003	13811 6	–	83147° 5
1411.946	2	70824.23	−.002	32843 2	–	103667° 3	1442.370	30	69330.33	−.003	12679 4	–	82009° 4
1412.042	100	70819.42	−.002	22890 0	–	93709° 1	1444.167	2	69244.06	−.005	19896 1	–	89139° 1
1412.915	50	70775.66	−.001	20611 4	–	91387° 4	1444.522	100	69227.05	−.002	14357 2	–	83584° 3
1413.081	2	70767.35	−.004	19487 3	–	90255° 4	1445.463	30	69181.98	−.003	16714 5	–	85896° 4
1413.921	30	70725.30	−.003	19576 2	–	90301° 1	1447.090	80	69104.20	.000	19487 3	–	88592° 2
1414.094	30	70716.65	−.001	32587 3	–	103303° 2	1447.366	30	69091.02	.000	64331 3	–	133422° 2
1414.632	100	70689.76	.005	19896 1	–	90586° 2	1447.638	100	69078.04	.000	20611 4	–	89689° 5
			−.008	32587 3	–	103276° 4	1447.710	50	69074.60	−.001	31323 2	–	100397° 3
					–		1448.950	200	69015.49	−.002	19576 2	–	88592° 2
449.035	30	69011.44	−.003	19487 3	–	88499° 3	1477.760	60	67669.98	.003	27006 3	–	94676° 3
1450.866	60bl	68924.35	−.003	64331 3	–	133255° 3	1477.865	100	67665.17	.003	32519 1	–	100184° 1
1450.901	200	68922.69	−.003	19576 2	–	88499° 3	1477.901	60	67663.53	−.001	12679 4	–	80343° 5
1451.043	10	68915.94	−.002	23183 2	–	92099° 2	1478.579	40	67632.50	.002	32587 3	–	100219° 2
1451.334	40	68902.12	−.001	58893 3	–	127795° 3	1480.063	10	67564.69	.003	32387 2	–	99952° 2
1451.547	300	68892.01	−.001	20611 4	–	89503° 4	1481.414	10	67503.07	.006	36164 4	–	103667° 3
1452.189	200	68861.56	.000	31323 2	–	100184° 1	1481.927	1	67479.70	−.003	56741 2	–	124221° 1
1452.562	2	68843.87	.004	74724° 3	–	143568 4				−.007	77557 2	–	145036° 1
			−.005	50362 1	–	119206° 1	1482.420	20	67457.26	.002	36164 4	–	103621° 5
1453.324	5	68807.78	−.001	50362 1	–	119170° 2	1482.956	10	67432.88	.001	32519 1	–	99952° 2
1454.045	100	68773.66	.002	19896 1	–	88669° 0	1483.328	1	67415.97	−.001	12679 4	–	80095° 4
1454.874	800	68734.47	−.002	13275 5	–	82009° 4	1483.425	10	67411.56	.003	22890 0	–	90301° 1
1455.688	80	68696.03	.001	19896 1	–	88592° 2	1483.482	2	67408.97	−.001	80343° 5	–	147752 5
1456.166	30	68673.48	−.004	15871 3	–	84544° 4				.005	49088 2	–	116497° 3
1457.481	500	68611.52	−.002	13928 2	–	82540° 2	1483.544	80	67406.15	.000	11271 0	–	78677° 1
1457.898	200	68591.90	−.001	13948 3	–	82540° 2				.006	33452 3	–	100858° 4
1458.890	30	68545.26	−.002	27006 3	–	95551° 2	1483.576	200	67404.70	.001	23183 2	–	90588° 3
1460.049	50	68490.85	.000	23183 2	–	91674° 2	1483.632	100	67402.15	.003	23183 2	–	90586° 2
1460.105	5	68488.22	.002	20611 4	–	89100° 4	1484.099	200	67380.95	.003	27006 3	–	94387° 2
1460.533	30	68468.15	−.001	19973 6	–	88441° 6	1484.201	1bl	67376.31	.008	32843 2	–	100219° 2
1460.713	80	68459.71	.001	32398 4	–	100858° 4	1484.458	100	67364.65	.005	32587 3	–	99952° 2
1461.089	5	68442.10	.002	58893 3	–	127336° 2	1485.841	300	67301.95	−.003	16282 4	–	83584° 3
1462.821	500	68361.06	−.002	12679 4	–	81040° 3	1487.772	1	67214.60	.004	78689° 6	–	145904 5
1463.430	5	68332.61	−.007	34225 4	–	102557° 3	1490.039	500	67112.33	.008	36164 4	–	103276° 4
1463.639	40	68322.85	−.002	19487 3	–	87810° 3				−.008	13928 2	–	81040° 3
1464.635	40	68276.39	.001	59059 2	–	127336° 2	1490.490	500	67092.03	−.008	27006 3	–	94098° 4
1464.746	80	68271.22	.002	32587 3	–	100858° 4				.009	13948 3	–	81040° 3
1464.950	200	68261.71	.002	16282 4	–	84544° 4	1491.209	1	67059.68	.000	48734 1	–	115794° 2
1465.105	200	68254.49	.001	19576 2	–	87831° 1	1491.887	60	67029.20	.001	33155 0	–	100184° 1
1465.246	40	68247.92	.002	49088 2	–	117336° 2	1492.357	1	67008.09	.006	77113° 5	–	144121 5
1465.543	5	68234.09	−.002	19576 2	–	87810° 3	1493.237	20	66968.60	−.005	51482 2	–	118451° 2
1466.649	20	68182.63	−.001	14357 2	–	82540° 2				.008	50318 5	–	117287° 4
1467.406	20	68147.46	−.004	35129 5	–	103276° 4	1493.477	300	66957.84	−.003	12510 1	–	79467° 2
1469.260	300	68061.47	.003	13948 3	–	82009° 4	1493.896	30	66939.06	−.003	19487 3	–	86426° 2
1469.437	10	68053.27	.008	51425 3	–	119479° 3	1494.197	30	66925.57	.000	32387 2	–	99313° 1
1470.372	80	68010.00	.005	32387 2	–	100397° 3	1494.339	1	66919.22	−.003	19973 6	–	86892° 5
1470.615	10	67998.76	.000	32398 4	–	100397° 3	1494.895	10	66894.33	.000	32418 1	–	99313° 1
1470.810	100	67989.74	−.001	46601 4	–	114591° 3	1494.919	5	66893.25	−.006	58893 3	–	125786° 3
			.004	31323 2	–	99313° 1	1495.211	5	66880.19	−.002	75816° 4	–	142696 4
1470.903	80	67985.44	−.001	19487 3	–	87473° 2	1495.542	200	66865.39	−.007	16282 4	–	83147° 5
1471.364	2	67964.14	−.006	54853 5	–	122817° 5	1496.362	200	66828.74	−.003	12679 4	–	79508° 3
1471.688	60	67949.18	.001	27006 3	–	94955° 4	1496.552	200	66820.26	−.004	13275 5	–	80095° 4
1471.993	80	67935.10	.002	19896 1	–	87831° 1	1497.144	200	66793.84	−.004	32519 1	–	99313° 1
1472.420	1	67915.40	.000	91098 4	–	159013° 5	1497.419	50	66781.57	.000	52697 4	–	119479° 3
1472.668	5	67903.96	−.001	19487 3	–	87391° 3	1499.133	20	66705.22	.002	49088 2	–	115794° 2
1472.826	10	67896.68	−.001	19576 2	–	87473° 2	1499.629	10	66683.16	−.001	14357 2	–	81040° 3
1473.030	10	67887.28	.001	20611 4	–	88499° 3	1499.945	500	66669.11	−.004	15871 3	–	82540° 2
1473.483	500	67866.40	.004	23183 2	–	91050° 3	1503.034	300	66532.09	−.002	13811 6	–	80343° 5
1474.592	200	67815.36	.004	47978 2	–	115794° 2	1503.672	200	66503.86	−.003	12510 1	–	79013° 2
1474.592	200	67815.36	−.005	19576 2	–	87391° 3	1503.758	1	66500.06	−.001	33452 3	–	99952° 2
					–					.002	72187 3	–	138688° 4
1474.706	100	67810.12	.004	32587 3	–	100397° 3						–	
1474.861	5	67803.00	.001	58730 4	–	126533° 4	1505.172	100	66437.59	−.001	12510 1	–	78947° 1
1474.950	30	67798.90	.000	23183 2	–	90982° 2	1505.262	500	66433.61	−.005	16714 5	–	83147° 5
1474.990	30	67797.07	.003	32387 2	–	100184° 1	1505.432	20	66426.11	−.007	13928 2	–	80354° 3
1475.284	1	67783.55	−.002	46299 3	–	114083° 2	1505.834	10	66408.38	−.002	19487 3	–	85896° 4
1475.668	5	67765.92	.001	32418 1	–	100184° 1	1505.882	40	66406.26	−.001	13948 3	–	80354° 3
1476.809	500	67713.56	−.005	15871 3	–	83584° 3	1506.173	30	66393.43	.005	36164 4	–	102557° 3
1477.093	60	67700.54	.002	32519 1	–	100219° 2	1508.735	60	66280.69	.001	20611 4	–	86892° 5
1477.629	100	67675.98	−.001	11271 0	–	78947° 1	1509.441	40	66249.69	.000	22890 0	–	89139° 1
1477.659	100	67674.61	.004	12679 4	–	80354° 3	1510.080	100	66221.65	−.002	13275 5	–	79497° 4
					–		1510.749	1	66192.33	.000	89482 3	–	155674° 4
511.144	40	66175.03	−.002	32387 2	–	98562° 3	1555.455	50	64289.87	−.007	23183 2	–	87473° 2
1511.308	50	66167.84	−.003	12510 1	–	78677° 1	1555.609	1	64283.50	−.001	83147° 5	–	147431 4
1511.415	20	66163.16	.007	32398 4	–	98562° 3	1556.398	2	64250.91	.001	32587 3	–	96838° 3
1511.539	50	66157.73	−.003	33155 0	–	99313° 1	1556.905	10	64229.99	−.007	13928 2	–	78158° 3
1511.774	40	66147.45	−.003	13948 3	–	80095° 4	1557.039	20	64224.46	−.001	15871 3	–	80095° 4
1511.968	500	66138.96	−.006	15871 3	–	82009° 4	1557.216	10	64217.16	−.002	32519 1	–	96736° 1
1512.709	30	66106.56	−.003	19576 2	–	85683° 1	1557.381	50	64210.36	−.005	13948 3	–	78158° 3
1512.962	500	66095.51	−.003	27006 3	–	93102° 4	1557.570	10	64202.57	−.003	32387 2	–	96589° 2
1515.215	500	65997.23	−.007	14357 2	–	80354° 3	1560.143	50	64096.68	−.001	19487 3	–	83584° 3
1515.723	40	65975.11	−.002	32587 3	–	98562° 3	1561.023	40	64060.55	−.002	16282 4	–	80343° 5
					–					−.002	79508° 3	–	143568 4
1516.160	60	65956.10	.000	23183 2	–	89139° 1						–	
1517.210	2	65910.45	.009	51425 3	–	117336° 2	1562.302	300	64008.11	−.006	19576 2	–	83584° 3
1518.330	10	65861.83	.001	51425 3	–	117287° 4	1562.442	50	64002.37	.004	32587 3	–	96589° 2
1518.514	1	65853.85	.001	51482 2	–	117336° 2	1564.151	40	63932.44	.005	20611 4	–	84544° 4
1518.783	200	65842.19	−.002	19487 3	–	85329° 2	1564.341	1	63924.68	.009	72356 2	–	136281° 3
1520.058	10	65786.96	.003	19896 1	–	85683° 1	1565.115	5	63893.06	.002	32843 2	–	96736° 1
1521.098	2	65741.98	.005	82009° 4	–	147752 5	1565.278	300	63886.41	.002	32398 4	–	96285° 5
1521.323	80	65732.26	.000	19576 2	–	85308° 1	1566.252	5	63846.68	.000	83584° 3	–	147431 4
1521.438	200	65727.29	−.002	16282 4	–	82009° 4	1566.471	10	63837.76	.000	13275 5	–	77113° 5
1523.613	2	65633.46	.000	64331 3	–	129964° 3	1567.080	10	63812.95	−.001	16282 4	–	80095° 4
					–		1567.376	200	63800.90	−.001	14357 2	–	78158° 3
1524.663	2	65588.26	.003	79508° 3	–	145096 4						–	
1524.862	50	65579.70	−.002	13928 2	–	79508° 3	1567.920	1h	63778.76	−.007	80343° 5	–	144121 5
1525.317	5	65560.14	−.002	13948 3	–	79508° 3	1571.410	5	63637.11	.000	15871 3	–	79508° 3
1525.581	50	65548.79	.001	13948 3	–	79497° 4	1571.613	200	63628.89	−.002	16714 5	–	80343° 5
1525.802	30	65539.30	−.002	13928 2	–	79467° 2	1571.687	30	63625.90	.000	15871 3	–	79497° 4
1526.258	100	65519.72	−.001	13948 3	–	79467° 2	1572.796	20	63581.03	.000	33155 0	–	96736° 1
1526.362	5	65515.25	.000	31323 2	–	96838° 3	1575.574	5	63468.93	.002	32387 2	–	95856° 3
1527.216	30	65478.62	.002	12679 4	–	78158° 3	1575.741	200	63462.20	−.002	12510 1	–	75972° 1
1528.745	100	65413.13	.005	31323 2	–	96736° 1	1575.859	300	63457.45	.003	32398 4	–	95856° 3
			−.005	19896 1	–	85308° 1	1577.750	20	63381.39	−.004	16714 5	–	80095° 4
					–		1578.442	100	63353.61	.000	31323 2	–	94676° 3
1528.857	80	65408.34	.001	23183 2	–	88592° 2						–	
1531.034	100	65315.33	.004	23183 2	–	88499° 3	1579.726	40	63302.11	−.001	13811 6	–	77113° 5
1531.158	100	65310.04	.003	32398 4	–	97709° 5	1580.553	60	63268.99	.002	32587 3	–	95856° 3
1531.762	10	65284.29	.001	20611 4	–	85896° 4	1581.070	10	63248.30	.003	27006 3	–	90255° 4
1532.176	100	65266.65	.003	31323 2	–	96589° 2	1581.641	2	63225.47	.004	16282 4	–	79508° 3
1534.463	100	65169.37	.003	15871 3	–	81040° 3				.003	80343° 5	–	143568 4
1534.902	50	65150.73	.001	14357 2	–	79508° 3	1581.916	30	63214.48	−.002	16282 4	–	79497° 4
1535.855	50	65110.31	.001	14357 2	–	79467° 2				−.004	80354° 3	–	143568 4
			−.004	33452 3	–	98562° 3	1582.924	1	63174.22	.000	19973 6	–	83147° 5
1536.445	5	65085.31	−.001	13928 2	–	79013° 2	1584.722	1	63102.55	−.003	80095° 4	–	143198 4
					–		1585.678	5	63064.50	.002	31323 2	–	94387° 2
1536.911	100	65065.57	.003	13948 3	–	79013° 2						–	
1537.124	200	65056.56	.002	19487 3	–	84544° 4	1586.483	30	63032.50	−.001	32519 1	–	95551° 2
1538.013	100	65018.95	.002	13928 2	–	78947° 1	1587.993	20	62972.57	.002	20611 4	–	83584° 3
1539.857	2	64941.09	.000	22890 0	–	87831° 1	1588.201	100	62964.32	.003	32587 3	–	95551° 2
1541.351	5	64878.14	.004	13811 6	–	78689° 6	1589.617	40	62908.23	−.003	13928 2	–	76836° 2
1542.346	1	64836.29	.001	83147° 5	–	147984 6	1590.113	300	62888.61	−.002	13948 3	–	76836° 2
1543.548	50	64785.80	.002	32398 4	–	97184° 4	1592.493	1	62794.62	−.004	31323 2	–	94117° 3
1544.209	10	64758.07	−.002	16282 4	–	81040° 3	1592.797	1	62782.64	.002	16714 5	–	79497° 4
1544.418	80	64749.31	−.002	13928 2	–	78677° 1	1593.938	2	62737.69	−.001	56741 2	–	119479° 3
1545.579	300	64700.67	−.003	11271 0	–	75972° 1	1596.321	100	62644.04	.002	19896 1	–	82540° 2
					–		1596.710	200	62628.78	.002	32387 2	–	95016° 1
1546.636	30	64656.45	−.001	14357 2	–	79013° 2						–	
1547.600	50	64616.18	.002	32519 1	–	97135° 0	1598.542	100	62557.00	−.001	32398 4	–	94955° 4
1548.052	200	64597.31	.002	32587 3	–	97184° 4	1598.951	40	62541.00	.000	13275 5	–	75816° 4
1549.577	30	64533.74	−.009	31323 2	–	95856° 3	1599.335	2h	62525.98	.000	79467° 2	–	141993 3
1550.784	30	64483.51	−.005	15871 3	–	80354° 3	1599.443	200	62521.76	.006	19487 3	–	82009° 4
1551.563	300	64451.13	−.006	32387 2	–	96838° 3	1600.071	40	62497.22	.000	27006 3	–	89503° 4
1551.843	60	64439.50	−.001	32398 4	–	96838° 3				−.007	32519 1	–	95016° 1
1551.984	1	64433.65	−.002	12679 4	–	77113° 5	1600.527	200	62479.42	−.004	14357 2	–	76836° 2
1554.565	200	64326.67	−.002	12510 1	–	76836° 2				−.007	80343° 5	–	142822 6
1554.716	40	64320.42	.000	19896 1	–	84216° 0	1602.079	1	62418.89	−.002	22890 0	–	85308° 1
			−.001	14357 2	–	78677° 1	1602.606	300	62398.36	.000	36164 4	–	98562° 3
1605.637	20bl	62280.57	.000	94098° 4	–	156378 4	1666.096	20	60020.55	−.003	19487 3	–	79508° 3
1605.707	300	62277.86	−.001	32398 4	–	94676° 3	1668.182	1	59945.49	−.005	15871 3	–	75816° 4
1608.822	1	62157.28	.002	81040° 3	–	143198 4	1669.310	5	59904.99	−.002	13948 3	–	73853° 2
1609.105	80	62146.34	−.002	23183 2	–	85329° 2	1669.690	5	59891.35	−.002	19576 2	–	79467° 2
1610.579	10	62089.47	−.003	32587 3	–	94676° 3	1670.689	20	59855.54	.002	32398 4	–	92254° 5
1611.765	300	62043.78	−.003	13928 2	–	75972° 1	1671.907	1	59811.93	.009	83584° 3	–	143396 3
1612.739	3h	62006.31	−.006	91098 4	–	153104° 3	1672.160	20	59802.89	.000	83147° 5	–	142950 5
1612.898	30	62000.20	.001	32387 2	–	94387° 2	1672.268	10	59799.02	−.001	83147° 5	–	142946 5
1613.546	30	61975.30	−.004	16714 5	–	78689° 6	1673.847	10	59742.61	.000	20611 4	–	80354° 3
1615.227	5	61910.80	.007	94676° 3	–	156587 3	1674.158	10	59731.51	−.006	20611 4	–	80343° 5
1615.372	10	61905.24	−.001	32387 2	–	94292° 1	1676.178	40	59659.53	−.001	31323 2	–	90982° 2
1615.528	30	61899.26	.000	31323 2	–	93222° 1	1677.473	5	59613.47	.001	83584° 3	–	143198 4
1616.138	20	61875.90	−.005	16282 4	–	78158° 3	1678.408	40	59580.26	−.002	32519 1	–	92099° 2
1616.335	80	61868.36	.000	32519 1	–	94387° 2	1678.645	1	59571.85	.002	19896 1	–	79467° 2
			−.003	13948 3	–	75816° 4	1679.474	40	59542.45	.000	88441° 6	–	147984 6
1616.526	40	61861.05	−.003	33155 0	–	95016° 1	1679.714	2	59533.94	−.004	16282 4	–	75816° 4
1618.115	80	61800.30	.001	32587 3	–	94387° 2	1680.329	1	59512.15	.001	32587 3	–	92099° 2
1620.760	10	61699.45	−.002	32398 4	–	94098° 4	1682.443	5	59437.37	−.002	19576 2	–	79013° 2
1621.900	2	61656.08	−.005	81040° 3	–	142696 4	1683.416	1	59403.02	.000	97184° 4	–	156587 3
1622.982	20	61614.97	−.005	14357 2	–	75972° 1	1684.739	200	59356.37	.002	23183 2	–	82540° 2
1624.825	10	61545.08	−.001	36164 4	–	97709° 5	1686.317	1	59300.83	−.005	72481 1	–	131782° 1
1625.216	30	61530.28	−.002	32587 3	–	94117° 3	1686.705	1	59287.19	−.003	32387 2	–	91674° 2
1625.729	5	61510.86	.001	32587 3	–	94098° 4	1687.393	5	59263.01	−.003	31323 2	–	90586° 2
1627.722	3	61435.55	−.001	31323 2	–	92758° 3	1689.067	2	59204.28	.003	88499° 3	–	147703 4
1628.049	1	61423.21	−.006	94955° 4	–	156378 4	1689.362	5	59193.94	.003	97184° 4	–	156378 4
1630.742	10	61321.77	.006	32387 2	–	93709° 1	1690.911	20	59139.71	−.001	75972° 1	–	135112 2
1632.518	20	61255.06	.000	32387 2	–	93642° 2	1691.790	1	59108.99	.000	84544° 4	–	143653 5
1634.303	5	61188.16	−.001	82009° 4	–	143198 4	1691.980	2	59102.35	−.006	16714 5	–	75816° 4
1635.660	5	61137.40	−.004	33155 0	–	94292° 1	1692.159	10	59096.10	−.001	43461 3	–	102557° 3
1637.864	1	61055.13	.001	32587 3	–	93642° 2	1692.413	10	59087.23	−.001	32587 3	–	91674° 2
1638.787	1	61020.74	.000	36164 4	–	97184° 4	1693.419	5	59052.13	.000	56741 2	–	115794° 2
1640.044	5	60973.97	−.002	83147° 5	–	144121 5	1693.589	20	59046.20	−.004	76836° 2	–	135882 3
1640.267	30	60965.68	−.002	15871 3	–	76836° 2	1694.499	5	59014.49	−.001	74724° 3	–	133739 4
1640.595	5h	60953.49	−.008	81040° 3	–	141993 3	1695.242	30	58988.62	−.001	32398 4	–	91387° 4
1642.932	50	60866.79	−.005	19487 3	–	80354° 3	1695.404	10	58982.99	.000	75816° 4	–	134799 5
1643.200	2	60856.86	−.006	82540° 2	–	143396 3	1695.529	10	58978.64	.002	31323 2	–	90301° 1
1643.282	5	60853.82	−.002	103485 4	–	164339° 3	1695.881	2	58966.40	−.008	44655 4	–	103621° 5
1643.793	1	60834.91	.000	32387 2	–	93222° 1	1696.873	1	58931.92	.003	88499° 3	–	147431 4
1644.839	2	60796.22	−.006	13928 2	–	74724° 3	1698.766	10bl	58866.25	−.001	77113° 5	–	135979 6
1645.334	10	60777.93	−.003	19576 2	–	80354° 3	1699.274	10	58848.66	−.003	75816° 4	–	134665 3
1645.371	100	60776.56	−.003	13948 3	–	74724° 3	1699.559	20	58838.79	−.003	74724° 3	–	133563 2
			−.003	31323 2	–	92099° 2	1699.952	50	58825.19	−.001	47978 2	–	106803° 3
1647.366	30	60702.96	.002	32519 1	–	93222° 1	1700.280	5	58813.84	−.009	73853° 2	–	132666 3
			.002	82009° 4	–	142712 5	1701.195	20	58782.20	−.008	19896 1	–	78677° 1
			.004	32398 4	–	93102° 4	1702.294	10	58744.25	−.002	77113° 5	–	135857 4
1647.809	20	60686.64	.001	82009° 4	–	142696 4	1703.685	5	58696.29	−.008	92884° 5	–	151580 5
1647.942	10	60681.74	−.003	83147° 5	–	143829 6	1704.428	40	58670.70	−.005	19487 3	–	78158° 3
1648.147	5	60674.20	.003	36164 4	–	96838° 3	1704.659	5	58662.75	.003	32387 2	–	91050° 3
1649.968	5	60607.23	.003	53407 0	–	114014° 1	1704.991	5	58651.33	.002	32398 4	–	91050° 3
1650.307	30	60594.78	.002	56741 2	–	117336° 2	1705.861	2	58621.42	.001	44655 4	–	103276° 4
1652.489	20	60514.77	−.004	32587 3	–	93102° 4	1706.249	2	58608.09	−.002	32398 4	–	91006° 5
1652.738	2	60505.65	.002	83147° 5	–	143653 5	1706.354	1	58604.48	−.003	76836° 2	–	135441 2
1654.354	1h	60446.55	.000	85896° 4	–	146342 5	1707.014	5	58581.82	−.002	19576 2	–	78158° 3
1654.834	2	60429.02	−.006	20611 4	–	81040° 3	1707.730	30	58557.26	.002	58730 4	–	117287° 4
1654.867	2	60427.81	−.007	55366 1	–	115794° 2	1710.486	20	58462.91	.000	32587 3	–	91050° 3
1655.656	30	60399.02	−.003	16714 5	–	77113° 5	1711.090	5	58442.27	−.008	16282 4	–	74724° 3
1656.430	5h	60370.79	.010	32387 2	–	92758° 3	1711.854	20	58416.19	−.002	78158° 3	–	136574 4
1656.736	10	60359.64	.002	32398 4	–	92758° 3	1712.144	5	58406.30	−.001	77557 2	–	135963° 2
1661.337	20	60192.48	.006	91387° 4	–	151580 5				−.005	84544° 4	–	142950 5
1664.819	2	60066.59	−.006	75816° 4	–	135882 3	1712.467	1	58395.28	−.002	32587 3	–	90982° 2
1713.056	5	58375.20	.000	73853° 2	–	132228 2	1750.539	1	57125.26	−.006	35129 5	–	92254° 5
1715.996	1	58275.19	.001	76836° 2	–	135112 2	1752.720	40	57054.17	.008	85896° 4	–	142950 5
1716.031	10	58274.00	.003	93306° 6	–	151580 5	1753.782	10	57019.63	.006	46601 4	–	103621° 5
1716.783	5	58248.48	−.007	89503° 4	–	147752 5	1755.323	1	56969.57	.007	88067 2	–	145036° 1
1717.841	10	58212.60	.001	75972° 1	–	134185 1	1755.823	20	56953.34	.009	78158° 3	–	135112 2
1718.258	40	58198.47	.002	32387 2	–	90586° 2	1756.216	2	56940.60	.007	19896 1	–	76836° 2
1719.151	2	58168.24	.005	84544° 4	–	142712 5	1756.303	500	56937.78	.006	36164 4	–	93102° 4
1721.246	2h	58097.44	−.001	72356 2	–	130453° 3	1757.557	10bl	56897.15	−.001	77113° 5	–	134010 6
1722.352	30	58060.14	−.001	73853° 2	–	131913 2	1757.643	1	56894.37	.003	79497° 4	–	136391 5
1722.719	50	58047.77	−.005	73853° 2	–	131900 1	1758.468	100	56867.68	.006	74724° 3	–	131592 3
1724.104	20	58001.14	−.002	32587 3	–	90588° 3	1759.457	5	56835.71	.006	43561 3	–	100397° 3
1724.672	5	57982.03	−.002	15871 3	–	73853° 2	1760.050	10	56816.56	.006	85896° 4	–	142712 5
1725.522	20	57953.47	.002	36164 4	–	94117° 3	1760.480	1	56802.69	.004	33452 3	–	90255° 4
1726.099	20	57934.10	.004	36164 4	–	94098° 4	1760.553	60	56800.33	.002	85896° 4	–	142696 4
1726.692	10	57914.20	.005	32387 2	–	90301° 1	1761.305	10	56776.08	.001	73853° 2	–	130629 1
1726.760	1	57911.92	−.007	88067 2	–	145978° 2	1761.774	30	56760.96	−.003	86892° 5	–	143653 5
1728.395	1	57857.14	−.005	23183 2	–	81040° 3	1762.041	30	56752.36	.000	32387 2	–	89139° 1
1728.622	10	57849.54	−.005	56741 2	–	114591° 3	1762.074	10bl	56751.30	.000	91006° 5	–	147758 6
1729.604	20	57816.70	−.008	98562° 3	–	156378 4	1762.845	10Hh	56726.48	.003	76836° 2	–	133563 2
			.000	31323 2	–	89139° 1	1763.042	100	56720.14	.000	36164 4	–	92884° 5
1729.636	20	57815.63	−.004	88441° 6	–	146257 7	1763.629	10	56701.26	.000	32398 4	–	89100° 4
1729.829	2	57809.18	.001	91006° 5	–	148816 5	1764.638	60	56668.84	.005	77113° 5	–	133782 5
1730.269	2	57794.48	−.006	73853° 2	–	131647 2	1764.935	5	56659.31	.000	46962 5	–	103621° 5
1731.078	2	57767.47	.000	58730 4	–	116497° 3	1765.135	10	56652.89	−.002	89689° 5	–	146342 5
1731.193	20	57763.63	.000	86892° 5	–	144656 4	1766.024	5	56624.37	−.001	94955° 4	–	151580 5
1732.656	5	57714.86	.001	49088 2	–	106803° 3	1767.469	10	56578.07	−.005	27006 3	–	83584° 3
1733.092	2	57700.34	.001	89482 3	–	147182° 2	1769.240	30	56521.44	.000	75816° 4	–	132337 3
1733.134	20h	57698.94	.003	78158° 3	–	135857 4	1769.522	300	56512.43	.000	73853° 2	–	130365 3
1733.923	1	57672.68	−.003	85896° 4	–	143568 4	1769.697	10bl	56506.84	−.007	78158° 3	–	134665 3
1734070	1	57667.79	−.004	32587 3	–	90255° 4	1770.029	10	56496.24	−.003	89482 3	–	145978° 2
1735.206	40	57630.04	−.002	75816° 4	–	133446 4	1770.123	5	56493.24	.006	78947° 1	–	135441 2
1736.379	30	57591.11	−.002	75972° 1	–	133563 2	1770.301	5	56487.56	.000	31323 2	–	87810° 3
1737.720	5	57546.67	−.004	20611 4	–	78158° 3	1770.869	2	56469.45	.000	89482 3	–	145951° 4
1737.837	40	57542.79	.001	73853° 2	–	131396 1	1772.578	20	56415.00	−.001	79467° 2	–	135882 3
1738.022	30	57536.67	−.004	75972° 1	–	133508 0	1773.036	2	56400.43	.000	89503° 4	–	145904 5
1738.339	30	57526.17	−.005	73853° 2	–	131379 2	1773.647	30	56381.00	−.002	91050° 3	–	147431 4
1739.112	30	57500.61	.000	85896° 4	–	143396 3	1774.389	100	56357.42	−.001	75816° 4	–	132173 4
1741.293	2	57428.58	.000	91387° 4	–	148816 5	1774.648	1	56349.20	−.002	79508° 3	–	135857 4
1742.240	1	57397.37	−.010	43461 3	–	100858° 4	1774.699	1	56347.58	.000	74724° 3	–	131072 2
1743.458	2	57357.27	−.004	32418 1	–	89775° 0	1774.863	5	56342.37	−.002	88441° 6	–	144783 6
1743.732	40	57348.26	−.003	76836° 2	–	134185 1	1775.146	50	56333.39	−.006	77113° 5	–	133446 4
1745.144	10	57301.86	−.001	85896° 4	–	143198 4	1775.288	2	56328.88	−.008	19487 3	–	75816° 4
1745.471	20	57291.12	−.003	32398 4	–	89689° 5	1775.692	5	56316.07	.001	91387° 4	–	147703 4
1745.500	2Hbl	57290.17	−.006	78689° 6	–	135979 6	1775.720	5Hbl	56315.18	−.006	76836° 2	–	133151 3
1745.615	1	57286.40	.003	42665 2	–	99952° 2	1776.308	100	56296.54	.000	32843 2	–	89139° 1
1745.728	50	57282.69	.003	78158° 3	–	135441 2	1777.749	1	56250.91	.005	32418 1	–	88669° 0
1745.795	1h	57280.49	.003	88067 2	–	145347° 2	1778.370	1	56231.26	.003	80343° 5	–	136574 4
1746.149	50	57268.88	.003	31323 2	–	88592° 2	1778.725	40	56220.04	.000	80354° 3	–	136574 4
1746.291	20	57264.22	.007	87391° 3	–	144656 4	1778.900	5h	56214.51	−.005	89689° 5	–	145904 5
1746.362	10	57261.89	.002	49541 4	–	106803° 3	1779.215	60	56204.56	.002	32387 2	–	88592° 2
1746.418	40	57260.06	.003	19576 2	–	76836° 2	1779.567	200	56193.44	.000	74724° 3	–	130918 4
1746.474	50	57258.22	−.001	77113° 5	–	134371 5	1779.672	30	56190.12	.000	100397° 3	–	156587 3
1746.528	50	57256.45	.000	32519 1	–	89775° 0	1780.088	50	56176.99	.000	87391° 3	–	143568 4
1747.364	5bl	57229.06	−.001	86892° 5	–	144121 5	1780.488	5	56164.37	−.002	78947° 1	–	135112 2
1747.674	20	57218.90	.006	73853° 2	–	131072 2	1780.721	1	56157.02	.001	88499° 3	–	144656 4
1748.387	10h	57195.57	.010	72187 3	–	129383° 4	1780.932	80	56150.37	.000	32519 1	–	88669° 0
1748.977	50	57176.28	−.003	90255° 4	–	147431 4				−.006	31323 2	–	87473° 2
			−.005	31323 2	–	88499° 3	1781.361	1	56136.85	.007	78158° 3	–	134295 3
1750.095	60	57139.75	.000	19973 6	–	77113° 5	1782.156	20	56111.81	−.002	32387 2	–	88499° 3
1750.273	20	57133.94	−.005	33452 3	–	90586° 2	1782.843	1	56090.18	.010	36164 4	–	92254° 5
1782.921	5	56087.73	−.001	90255° 4	–	146342 5	1809.789	60	55255.06	.003	92728° 6	–	147984 6
1783.277	20	56076.53	−.006	19896 1	–	75972° 1	1810.350	200	55237.93	.000	91098 4	–	146336° 4
1783.521	1	56068.86	−.006	31323 2	–	87391° 3	1810.465	50	55234.42	−.003	92728° 6	–	147963 7
1783.865	40	56058.05	−.001	86892° 5	–	142950 5	1810.831	30	55223.26	.001	32587 3	–	87810° 3
1783.990	60	56054.12	.000	86892° 5	–	142946 5				.007	36164 4	–	91387° 4
1785.563	40	56004.74	−.001	32587 3	–	88592° 2	1811.944	20	55189.34	−.003	58893 3	–	114083° 2
1786.568	5h	55973.24	.001	79467° 2	–	135441 2	1812.361	10	55176.64	.002	92254° 5	–	147431 4
1787.222	10	55952.75	−.007	49088 2	–	105041° 1	1812.546	10h	55171.01	.000	79013° 2	–	134185 1
1787.598	10	55940.98	−.002	75972° 1	–	131913 2	1812.707	20	55166.11	−.005	80095° 4	–	135261 4
1787.866	2	55932.60	.009	79508° 3	–	135441 2	1813.299	5	55148.10	.000	72187 3	–	127336° 2
					–					−.001	19576 2	–	74724° 3
1787.958	200	55929.72	−.001	86892° 5	–	142822 6						–	
1788.224	200	55921.40	.000	77113° 5	–	133034 4	1813.572	20	55139.80	.001	33452 3	–	88592° 2
1788.529	10	55911.86	−.001	32587 3	–	88499° 3	1813.666	50	55136.94	.003	88067 2	–	143204° 2
1789.748	40	55873.78	.000	91098 4	–	146972° 3	1814.828	40	55101.64	.000	75816° 4	–	130918 4
1790.027	30	55865.07	−.001	89482 3	–	145347° 2	1814.882	100	55100.00	−.002	75972° 1	–	131072 2
1790.168	20	55860.67	.004	58730 4	–	114591° 3				−.003	92884° 5	–	147984 6
1790.895	200	55838.00	.001	77113° 5	–	132951 5	1815.078	200	55094.05	.000	89689° 5	–	144783 6
1791.991	20	55803.85	.004	86892° 5	–	142696 4	1815.117	200h	55092.86	−.003	78689° 6	–	133782 5
1792.398	300	55791.18	.005	75816° 4	–	131607 5	1815.309	500	55087.04	−.001	88441° 6	–	143528 7
1792.865	5	55776.64	.005	48734 1	–	104511° 2	1815.658	50	55076.45	.001	76836° 2	–	131913 2
					–		1815.884	40	55069.59	.000	88499° 3	–	143568 4
1793.251	2	55764.64	−.006	79497° 4	–	135261 4						–	
1793.975	3h	55742.13	.004	88067 2	–	143809° 3	1816.070	2	55063.95	.001	76836° 2	–	131900 1
			.008	44655 4	–	100397° 3	1817.412	30	55023.29	−.005	92728° 6	–	147752 5
1794.398	40	55728.99	.004	100858° 4	–	156587 3				.005	59059 2	–	114083° 2
1794.884	10h	55713.90	.006	93102° 4	–	148816 5	1818.547	2	54988.95	−.001	102557° 3	–	157546 3
1795.405	40	55697.74	.008	77557 2	–	133255° 3	1818.582	20	54987.89	.001	32843 2	–	87831° 1
1795.910	100	55682.07	−.003	78689° 6	–	134371 5	1819.010	50	54974.95	−.008	23183 2	–	78158° 3
1795.974	80	55680.09	−.003	88441° 6	–	144121 5	1819.296	40	54966.31	.001	89689° 5	–	144656 4
1796.135	40	55675.10	.000	75972° 1	–	131647 2	1819.661	20	54955.29	−.002	91387° 4	–	146342 5
1796.912	5	55651.02	.002	79013° 2	–	134665 3				−.005	59059 2	–	114014° 1
					–		1819.703	30	54954.02	−.002	32519 1	–	87473° 2
1796.975	2	55649.07	.005	90255° 4	–	145904 5						–	
1797.017	2	55647.77	.005	33452 3	–	89100° 4	1821.010	5	54914.58	.000	75972° 1	–	130886 1
1797.136	30	55644.09	.001	79467° 2	–	135112 2	1821.257	30	54907.13	−.002	80354° 3	–	135261 4
1797.393	40bl	55636.13	.006	80343° 5	–	135979 6	1821.571	40bl	54897.66	−.003	88499° 3	–	143396 3
1797.435	100	55634.83	.002	88067 2	–	143701° 3	1821.955	80	54886.09	−.005	87810° 3	–	142696 4
1798.782	100	55593.17	.000	89482 3	–	145075° 4				.006	36164 4	–	91050° 3
1799.626	5	55567.10	.000	86426° 2	–	141993 3				−.006	32587 3	–	87473° 2
1799.944	200	55557.28	−.003	91098 1	–	146655° 5	1822.284	100	54876.18	.003	78158° 3	–	133034 4
1800.884	20	55528.28	−.003	103485 4	–	159013° 5	1822.354	200	54874.08	−.002	92884° 5	–	147758 6
			.004	80354° 3	–	135882 3	1822.560	10	54867.87	.001	92884° 5	–	147752 5
					–		1823.039	50	54853.46	.001	91098 4	–	145951° 4
1801.148	50bl	55520.14	.000	100858° 4	–	156378 4						–	
1801.200	50bl	55518.54	.008	88067 2	–	143585° 2	1823.395	50	54842.75	.004	36164 4	–	91006° 5
1801.342	40	55514.16	.003	80343° 5	–	135857 4	1823.510	20	54839.29	−.005	64331 3	–	119170° 2
1801.882	50	55497.53	.002	92254° 5	–	147752 5	1823.791	1	54830.84	.004	78677° 1	–	133508 0
1802.962	10	55464.28	.008	34225 4	–	89689° 5	1823.893	50	54827.77	−.007	79467° 2	–	134295 3
1803.456	1	55449.09	.000	92254° 5	–	147703 4	1824.172	50	54819.39	.001	92884° 5	–	147703 4
1803.664	100	55442.69	.003	76836° 2	–	132279 3	1824.461	30	54810.70	−.003	76836° 2	–	131647 2
1803.778	1	55439.19	.007	72356 2	–	127795° 3	1824.656	5	54804.85	−.004	88592° 2	–	143396 3
1804.289	20	55423.49	.005	75972° 1	–	131396 1	1826.233	1	54757.52	.001	80354° 3	–	135112 2
1804.649	3	55412.43	.003	32418 1	–	87831° 1	1826.293	50	54755.72	.002	76836° 2	–	131592 3
					–		1828.024	20	54703.87	.000	80095° 4	–	134799 5
1805.335	10	55391.38	.006	76836° 2	–	132228 2						–	
1805.451	60	55387.82	−.002	88441° 6	–	143829 6	1828.195	1	54698.76	.002	88499° 3	–	143198 4
1805.787	3	55377.51	.007	51425 3	–	106803° 3	1828.870	10	54678.57	.004	50362 1	–	105041° 1
1807.150	10	55335.75	.003	91006° 5	–	146342 5	1828.962	80	54675.82	−.001	33155 0	–	87831° 1
1807.337	80bl	55330.02	−.002	89482 3	–	144812° 3	1829.076	10	54672.41	−.004	96907° 6	–	151580 5
1807.485	20	55325.49	.001	47978 2	–	103303° 2				.007	92758° 3	–	147431 4
1807.952	500	55311.20	−.003	77113° 5	–	132424 6	1829.587	300	54657.14	.002	93306° 6	–	147963 7
1808.244	10bl	55302.27	.004	79497° 4	–	134799 5				−.007	75972° 1	–	130629 1
1808.672	2	55289.18	.001	93306° 6	–	148595 7	1829.887	500	54648.18	.000	78689° 6	–	133337 7
1808.714	10	55287.90	.006	78158° 3	–	133446 4	1830.495	30	54630.03	.000	32843 2	–	87473° 2
					–		1830.907	80	54617.73	.002	89503° 4	–	144121 5
1830.987	40	54615.35	.010	78947° 1	–	133563 2	1856.661	1h	53860.12	.004	94955° 4	–	148816 5
1832.212	10h	54578.83	−.006	49088 2	–	103667° 3	1856.988	100	53850.64	−.006	89100° 4	–	142950 5
1832.524	1	54569.54	.004	48734 1	–	103303° 2	1857.124	50	53846.70	−.004	89100° 4	–	142946 5
			−.003	80095° 4	–	134665 3	1858.255	10	53813.92	.002	52697 4	–	106511° 4
1832.686	2h	54564.72	−.003	82009° 4	–	136574 4	1858.871	30	53796.09	.000	46601 4	–	100397° 3
1832.807	2h	54561.11	.001	78947° 1	–	133508 0	1859.529	80	53777.05	.000	91006° 5	–	144783 6
1832.873	1	54559.15	.001	76836° 2	–	131396 1	1860.298	40	53754.82	.002	78158° 3	–	131913 2
			−.002	46299 3	–	100858° 4	1860.985	20	53734.98	−.003	78689° 6	–	132424 6
1833.281	5	54547.01	.001	92884° 5	–	147431 4				.003	49541 4	–	103276° 4
1834.171	5	54520.54	.002	87473° 2	–	141993 3	1861.395	10	53723.14	.000	43461 3	–	97184° 4
1834.301	10	54516.68	.003	91387° 4	–	145904 5	1861.457	1h	53721.35	.005	89482 3	–	143204° 2
1834.585	80	54508.24	.001	78158° 3	–	132666 3	1861.879	2	53709.18	.002	91387° 4	–	145096 4
			.001	90588° 3	–	145096 4	1862.403	20	53694.07	.004	89503° 4	–	143198 4
1835.040	30bl	54494.72	−.005	77113° 5	–	131607 5	1862.657	50	53686.75	−.002	80095° 4	–	133782 5
1835.918	10	54468.66	−.001	89100° 4	–	143568 4	1863.335	300	53667.21	.000	80343° 5	–	134010 6
1836.475	10	54452.14	−.001	93306° 6	–	147758 6	1863.771	30	53654.66	.002	79497° 4	–	133151 3
1836.683	60	54445.97	.001	93306° 6	–	147752 5	1863.836	5	53652.78	−.002	79013° 2	–	132666 3
1837.413	40	54424.34	.003	36164 4	–	90588° 3				−.004	46299 3	–	99952° 2
1838.189	5	54401.37	−.005	90255° 4	–	144656 4	1863.951	10	53649.47	−.004	91006° 5	–	144656 4
1838.890	20	54380.63	.002	88441° 6	–	142822 6	1864.870	30	53623.04	−.001	43561 3	–	97184° 4
1839.638	30	54358.52	−.003	33452 3	–	87810° 3	1865.474	40D	53605.67	.010	91050° 3	–	144656 4
1840.539	10	54331.91	−.006	42404 1	–	96736° 1	1866.184	100	53585.28	.000	34225 4	–	87810° 3
1840.715	10	54326.71	.000	89482 3	–	143809° 3	1866.247	2	53583.47	.002	32843 2	–	86426° 2
1841.260	10	54310.63	−.002	80354° 3	–	134665 3	1867.068	50	53559.91	.004	94424° 7	–	147984 6
1842.119	200	54285.30	−.004	79497° 4	–	133782 5	1867.399	10	53550.42	.001	78677° 1	–	132228 2
1842.431	2h	54276.11	−.008	80095° 4	–	134371 5	1867.465	5	53548.52	.001	92728° 6	–	146277 6
1842.641	5h	54269.93	−.002	103276° 4	–	157546 3	1867.792	5bl	53539.15	.003	94424° 7	–	147963 7
1842.916	2	54261.83	−.001	78689° 6	–	132951 5	1867.846	40h	53537.60	.000	79497° 4	–	133034 4
1843.074	10	54257.18	−.003	46601 4	–	100858° 4	1868.176	100	53528.14	.001	92728° 6	–	146257 7
1843.567	10	54242.67	−.002	103303° 2	–	157546 3	1868.257	300	53525.82	.002	36164 4	–	89689° 5
1843.811	40	54235.49	.000	76836° 2	–	131072 2	1869.345	50	53494.67	.001	88499° 3	–	141993 3
1845.109	40	54197.34	.002	88499° 3	–	142696 4	1869.547	10	53488.89	.005	78158° 3	–	131647 2
1845.523	1	54185.18	.000	42404 1	–	96589° 2	1870.612	30	53458.44	.004	92884° 5	–	146342 5
1845.952	5	54172.59	−.001	42665 2	–	96838° 3	1870.765	40	53454.06	.005	79497° 4	–	132951 5
1846.739	30	54149.50	.005	89503° 4	–	143653 5	1871.022	1h	53446.72	.003	89503° 4	–	142950 5
1847.082	80	54139.44	.001	89689° 5	–	143829 6	1871.157	30	53442.87	.001	89503° 4	–	142946 5
1848.240	80	54105.52	.005	52697 4	–	106803° 3	1871.291	80	53439.04	.002	80343° 5	–	133782 5
1848.497	10	54098.00	.003	46299 3	–	100397° 3	1871.465	20	53434.07	.004	78158° 3	–	131592 3
			−.005	89100° 4	–	143198 4	1872.595	20	53401.83	.000	88592° 2	–	141993 3
1848.587	5	54095.37	.003	79467° 2	–	133563 2	1872.713	20	53398.46	−.001	90255° 4	–	143653 5
1848.742	80	54090.83	.006	36164 4	–	90255° 4	1872.893	50	53393.33	.000	92884° 5	–	146277 6
1848.834	10	54088.14	.003	92254° 5	–	146342 5	1874.380	80	53350.97	−.003	80095° 4	–	133446 4
1849.532	10	54067.73	.002	90588° 3	–	144656 4	1874.773	20	53339.79	.001	36164 4	–	89503° 4
1850.139	30	54049.99	.005	76836° 2	–	130886 1	1874.953	5	53334.67	−.002	42521 2	–	95856° 3
1850.878	100	54028.41	−.004	80343° 5	–	134371 5	1875.328	20	53324.00	−.001	79013° 2	–	132337 3
1851.063	50	54023.01	.000	92254° 5	–	146277 6	1875.692	50	53313.65	.000	94117° 3	–	147431 4
1851.130	40	54021.05	.000	33452 3	–	87473° 2				.003	90255° 4	–	143568 4
1851.324	20	54015.39	.003	78158° 3	–	132173 4	1875.791	20h	53310.84	.008	103276° 4	–	156587 3
1851.617	200	54006.84	.001	31323 2	–	85329° 2	1876.856	2	53280.59	.002	78947° 1	–	132228 2
1852.339	50	53985.79	.001	31323 2	–	85308° 1	1877.001	1	53276.47	.004	43561 3	–	96838° 3
1852.857	100	53970.70	.000	35129 5	–	89100° 4	1877.273	30	53268.75	.000	91387° 4	–	144656 4
1853.322	5	53957.16	.001	19896 1	–	73853° 2	1877.383	20	53265.63	−.001	79013° 2	–	132279 3
1853.589	60	53949.39	.000	79497° 4	–	133446 4	1877.757	100bl	53255.02	−.008	81040° 3	–	134295 3
1853.880	5	53940.92	.003	80354° 3	–	134295 3	1877.872	100	53251.76	−.004	82009° 4	–	135261 4
1853.932	5	53939.41	.005	33452 3	–	87391° 3	1878.152	100	53243.82	−.001	83147° 5	–	136391 5
1854.589	20	53920.30	.003	46299 3	–	100219° 2	1878.265	80	53240.62	.004	93102° 4	–	146342 5
1855.155	30	53903.85	.004	32418 1	–	86322° 0	1878.895	50	53222.77	.003	78677° 1	–	131900 1
1855.399	100	53896.76	−.007	46962 5	–	100858° 4	1878.967	40	53220.73	.002	78158° 3	–	131379 2
1856.016	20	53878.84	.002	89689° 5	–	143568 4	1879.189	50	53214.44	−.001	79013° 2	–	132228 2
1856.445	1h	53866.39	.007	90255° 4	–	144121 5	1879.380	50	53209.03	.002	89503° 4	–	142712 5
					–					−.004	80354° 3	–	133563 2
1879.954	5Hbl	53192.79	−.001	89503° 4	–	142696 4	1897.829	30	52691.78	−.003	90255° 4	–	142946 5
1880.564	2	53175.53	−.007	92728° 6	–	145904 5	1898.246	60h	52680.21	.000	80354° 3	–	133034 4
1880.774	50h	53169.59	.000	79497° 4	–	132666 3	1898.369	30	52676.79	.000	79497° 4	–	132173 4
1880.885	50	53166.46	−.002	34225 4	–	87391° 3	1898.775	200	52665.53	.001	79508° 3	–	132173 4
1880.979	1	53163.80	−.002	32519 1	–	85683° 1	1899.145	60	52655.27	−.004	82009° 4	–	134665 3
1881.173	100	53158.32	.002	79508° 3	–	132666 3	1899.454	100	52646.70	−.004	91006° 5	–	143653 5
1881.753	5	53141.93	−.007	90255° 4	–	143396 3	1900.794	40	52609.59	.000	90588° 3	–	143198 4
1881.829	100	53139.79	.000	50481 6	–	103621° 5	1900.849	40	52608.07	.001	80343° 5	–	132951 5
1882.090	30	53132.42	.000	89689° 5	–	142822 6	1902.153	100D	52572.00	−.003	82540° 2	–	135112 2
1882.718	20	53114.69	.002	91006° 5	–	144121 5	1902.182	20bl	52571.20	−.004	80095° 4	–	132666 3
1883.120	30	53103.36	−.002	80343° 5	–	133446 4	1902.521	1	52561.83	.002	91006° 5	–	143568 4
1883.164	20	53102.12	−.001	103276° 4	–	156378 4	1903.648	5	52530.72	.000	96285° 5	–	148816 5
1884.792	40	53056.25	−.001	80095° 4	–	133151 3	1903.694	10	52529.45	.000	92254° 5	–	144783 6
1884.880	200	53053.77	.001	44655 4	–	97709° 5				.001	44655 4	–	97184° 4
1885.531	5	53035.45	.001	104511° 2	–	157546 3	1903.940	40	52522.66	.002	81040° 3	–	133563 2
1885.786	30	53028.28	−.001	51482 2	–	104511° 2	1904.090	10	52518.52	−.001	91050° 3	–	143568 4
			−.006	43561 3	–	96589° 2	1904.174	2	52516.20	.003	77557 2	–	130073° 2
1885.974	80	53023.00	.001	89689° 5	–	142712 5	1904.958	30	52494.59	−.004	42521 2	–	95016° 1
1886.078	60	53020.07	.001	92884° 5	–	145904 5	1905.119	30	52490.15	−.004	89503° 4	–	141993 3
1886.223	100	53016.00	.000	49541 4	–	102557° 3	1905.656	20	52475.36	.000	94955° 4	–	147431 4
1886.593	40	53005.60	−.001	64331 3	–	117336° 2	1906.009	60	52465.64	.000	32843 2	–	85308° 1
1886.684	20	53003.04	−.003	72356 2	–	125359° 2	1906.290	60	52457.91	−.001	90255° 4	–	142712 5
1887.493	5	52980.32	.000	90588° 3	–	143568 4	1906.635	30D	52448.42	−.007	78947° 1	–	131396 1
1887.809	100	52971.46	−.001	93306° 6	–	146277 6	1906.745	30	52445.39	−.001	79467° 2	–	131913 2
1888.293	100	52957.88	.001	50318 5	–	103276° 4	1906.883	30	52441.60	−.002	90255° 4	–	142696 4
1888.465	50	52953.06	−.001	78947° 1	–	131900 1	1908.602	40h	52394.37	.000	78677° 1	–	131072 2
1888.535	50	52951.09	−.002	93306° 6	–	146257 7	1909.052	5h	52382.02	.000	79013° 2	–	131396 1
1888.825	60	52942.96	.001	90255° 4	–	143198 4	1909.663	10	52365.26	−.001	79013° 2	–	131379 2
1890.329	5h	52900.84	.008	82540° 2	–	135441 2	1910.199	10	52350.56	−.001	42665 2	–	95016° 1
1890.380	50	52899.41	−.003	79013° 2	–	131913 2	1910.346	5	52346.53	−.001	91050° 3	–	143396 3
1890.709	30	52890.21	.002	32418 1	–	85308° 1	1910.654	50	52338.10	.001	92758° 3	–	145096 4
1890.831	20	52886.80	.001	79013° 2	–	131900 1	1910.757	80	52335.27	−.004	36164 4	–	88499° 3
1891.438	20	52869.82	.007	79467° 2	–	132337 3	1911.366	50	52318.60	−.001	78568° 0	–	130886 1
1891.941	200	52855.77	−.003	80095° 4	–	132951 5	1912.105	100	52298.38	.000	83584° 3	–	135882 3
1892.421	40	52842.36	−.005	92254° 5	–	145096 4	1912.239	1	52294.71	−.001	43561 3	–	95856° 3
1892.477	40h	52840.80	.002	79497° 4	–	132337 3	1913.034	30	52272.98	−.002	83584° 3	–	135857 4
1892.800	200	52831.78	−.002	83147° 5	–	135979 6	1913.290	50	52265.99	.000	91387° 4	–	143653 5
1892.877	40	52829.63	.000	79508° 3	–	132337 3	1914.074	5h	52244.58	−.004	94098° 4	–	146342 5
1892.950	10h	52827.59	.001	78568° 0	–	131396 1	1914.155	50	52242.37	−.001	80095° 4	–	132337 3
1893.131	80	52822.54	−.002	91006° 5	–	143829 6	1914.188	50	52241.47	.001	47978 2	–	100219° 2
					–					.001	51425 3	–	103667° 3
1893.519	30bl	52811.72	−.002	79467° 2	–	132279 3						–	
1893.553	60	52810.77	.001	90586° 2	–	143396 3	1914.604	10	52230.12	.003	52811 1	–	105041° 1
			−.005	32519 1	–	85329° 2	1915.380	10	52208.96	.002	78677° 1	–	130886 1
1893.645	2h	52808.20	.005	90588° 3	–	143396 3	1915.483	40	52206.15	.001	47978 2	–	100184° 1
1894.036	60	52797.30	.001	80354° 3	–	133151 3	1916.112	10	52189.01	.004	103485 4	–	155674° 4
1894.313	200	52789.58	.000	82009° 4	–	134799 5	1916.321	5	52183.32	−.010	44655 4	–	96838° 3
			.000	32519 1	–	85308° 1	1916.460	5	52179.53	−.001	79467° 2	–	131647 2
1894.561	10	52782.67	−.006	79497° 4	–	132279 3	1916.927	5	52166.82	−.002	64331 3	–	116497° 3
1894.977	2	52771.08	.006	79508° 3	–	132279 3	1917.413	40	52153.60	−.002	33155 0	–	85308° 1
1895.357	100	52760.50	−.001	79467° 2	–	132228 2	1917.628	20bl	52147.75	−.001	91050° 3	–	143198 4
					–		1917.702	300	52145.74	−.004	32398 4	–	84544° 4
1895.468	50	52757.41	.000	103621° 5	–	156378 4						–	
1895.999	10	52742.64	−.001	32587 3	–	85329° 2	1917.946	5	52139.11	.000	79508° 3	–	131647 2
1896.303	30	52734.18	−.001	91387° 4	–	144121 5	1918.467	40	52124.95	−.001	82540° 2	–	134665 3
1896.810	40	52720.09	−.001	79508° 3	–	132228 2	1918.993	20	52110.66	.003	79497° 4	–	131607 5
1896.887	20	52717.95	.004	78677° 1	–	131396 1	1919.087	10	52108.11	.001	90588° 3	–	142696 4
1897.182	100bl	52709.75	−.002	83147° 5	–	135857 4	1920.077	60	52081.24	−.001	80343° 5	–	132424 6
1897.488	100	52701.25	.000	78677° 1	–	131379 2	1920.922	20	52058.33	−.001	79013° 2	–	131072 2
1897.546	50bl	52699.64	.002	78947° 1	–	131647 2	1921.968	200	52030.00	.001	84544° 4	–	136574 4
1897.590	500	52698.42	.002	81040° 3	–	133739 4	1922.737	2h	52009.19	.004	91387° 4	–	143396 3
1897.686	20	52695.75	−.006	90255° 4	–	142950 5	1923.447	40	51989.99	.002	43561 3	–	95551° 2
					–		1924.047	1	51973.78	.000	47978 2	–	99952° 2
1924.663	60	51957.14	.000	32587 3	–	84544° 4	1953.672	50	51185.66	−.001	32398 4	–	83584° 3
1924.882	20	51951.23	.005	78677° 1	–	130629 1	1954.603	5	51161.28	.003	79467° 2	–	130629 1
1925.159	20	51943.76	.000	91006° 5	–	142950 5	1955.341	1h	51141.97	−.002	82009° 4	–	133151 3
1925.305	40h	51939.82	.001	91006° 5	–	142946 5	1955.733	10	51131.72	.002	51425 3	–	102557° 3
1925.854	10	51925.01	.003	80354° 3	–	132279 3	1956.156	10	51120.66	.001	42521 2	–	93642° 2
1926.478	80	51908.19	−.001	96907° 6	–	148816 5	1956.377	5	51114.89	.003	43561 3	–	94676° 3
1926.792	5h	51899.73	.002	92884° 5	–	144783 6	1957.103	5	51095.93	.000	49088 2	–	100184° 1
1927.609	50	51877.74	.001	33452 3	–	85329° 2	1957.240	10	51092.35	−.002	33452 3	–	84544° 4
1927.866	40	51870.82	.002	79508° 3	–	131379 2	1957.695	40	51080.48	.002	83584° 3	–	134665 3
1928.051	40	51865.84	.001	42521 2	–	94387° 2	1957.857	20	51076.25	−.003	96907° 6	–	147984 6
1928.400	40	51856.46	.008	83584° 3	–	135441 2	1958.656	10h	51055.41	−.001	96907° 6	–	147963 7
1928.751	100	51847.02	.001	84544° 4	–	136391 5	1959.453	10	51034.65	.000	106511° 4	–	157546 3
			.005	95856° 3	–	147703 4	1959.834	10	51024.73	.003	82009° 4	–	133034 4
1929.708	1	51821.31	−.008	51482 2	–	103303° 2				−.001	80354° 3	–	131379 2
1929.778	1h	51819.43	−.001	80354° 3	–	132173 4	1959.890	20	51023.27	.000	82540° 2	–	133563 2
1929.928	1h	51815.40	.001	91006° 5	–	142822 6	1960.028	5	51019.67	−.002	93102° 4	–	144121 5
1930.104	1h	51810.68	−.005	91387° 4	–	143198 4	1960.893	200	50997.17	−.001	32587 3	–	83584° 3
1930.278	40	51806.00	.000	94098° 4	–	145904 5	1961.587	5h	50979.13	.001	94117° 3	–	145096 4
1930.585	2	51797.77	−.002	32418 1	–	84216° 0	1961.671	5	50976.94	−.008	42665 2	–	93642° 2
1931.591	5	51770.79	.001	42521 2	–	94292° 1	1962.779	1	50948.17	.010	94955° 4	–	145904 5
1931.872	2	51763.26	−.004	35129 5	–	86892° 5	1962.956	5	50943.57	.001	91050° 3	–	141993 3
1932.165	80	51755.41	−.002	82540° 2	–	134295 3				−.002	92254° 5	–	143198 4
1932.783	40	51738.86	−.001	90255° 4	–	141993 3	1963.039	5	50941.42	−.001	82009° 4	–	132951 5
1934.283	20h	51698.74	−.001	96285° 5	–	147984 6	1963.630	2	50926.09	.000	43461 3	–	94387° 2
1934.343	50	51697.14	−.003	32519 1	–	84216° 0	1963.692	2	50924.48	.003	92728° 6	–	143653 5
1934.709	40	51687.36	.001	96907° 6	–	148595 7	1966.339	10	50855.93	.003	49541 4	–	100397° 3
1935.095	200	51677.04	−.001	83584° 3	–	135261 4	1966.796	2h	50844.11	.003	96907° 6	–	147752 5
1935.789	300	51658.52	.003	54853 5	–	106511° 4	1967.504	40	50825.81	.005	43561 3	–	94387° 2
1936.709	1	51633.98	−.005	53407 0	–	105041° 1	1967.744	10	50819.61	.000	72187 3	–	123007° 3
1936.853	20	51630.14	−.002	44655 4	–	96285° 5	1968.106	2	50810.27	−.003	92758° 3	–	143568 4
1936.973	1	51626.94	−.002	42665 2	–	94292° 1	1968.511	50	50799.81	−.005	92728° 6	–	143528 7
1937.005	5h	51626.09	−.003	81040° 3	–	132666 3	1970.484	10	50748.95	−.003	32398 4	–	83147° 5
1937.407	10	51615.38	−.002	79013° 2	–	130629 1	1970.558	10	50747.04	−.003	46962 5	–	97709° 5
1937.817	1	51604.46	−.003	79467° 2	–	131072 2	1970.714	5	50743.03	−.002	106803° 3	–	157546 3
1938.924	5	51574.99	−.004	92254° 5	–	143829 6	1970.782	10	50741.27	−.001	32843 2	–	83584° 3
1939.342	5	51563.88	.003	79508° 3	–	131072 2	1971.272	2	50728.66	−.003	36164 4	–	86892° 5
1939.531	20	51558.85	−.002	80354° 3	–	131913 2	1974.934	10	50634.60	−.002	83147° 5	–	133782 5
1939.706	10	51554.20	.002	93102° 4	–	144656 4	1976.017	1	50606.85	−.004	81040° 3	–	131647 2
1940.710	10	51527.53	.000	83584° 3	–	135112 2	1976.910	100	50583.99	−.003	47978 2	–	98562° 3
1941.286	30	51512.24	.000	80095° 4	–	131607 5	1976.940	60	50583.22	−.001	46601 4	–	97184° 4
1943.007	5	51466.61	.004	96285° 5	–	147752 5	1977.709	1	50563.55	.004	80354° 3	–	130918 4
1943.147	20	51462.91	−.002	64331 3	–	115794° 2	1978.010	5h	50555.86	−.002	43561 3	–	94117° 3
1944.142	40bl	51436.57	.001	82009° 4	–	133446 4	1978.636	20	50539.86	−.001	50318 5	–	100858° 4
1944.807	10	51418.98	.001	79467° 2	–	130886 1	1978.680	40	50538.74	.001	46299 3	–	96838° 3
1945.157	20	51409.73	.004	79508° 3	–	130918 4				−.002	94117° 3	–	144656 4
1945.746	5	51394.17	−.003	43561 3	–	94955° 4	1979.458	1	50518.87	−.002	97184° 4	–	147703 4
1945.801	40	51392.71	−.001	92728° 6	–	144121 5	1980.476	1	50492.91	.000	52811 1	–	103303° 2
1947.359	5	51351.60	.001	79013° 2	–	130365 3	1983.343	40	50419.92	.003	94676° 3	–	145096 4
1947.860	30	51338.39	.000	84544° 4	–	135882 3	1987.289	10	50319.80	.003	54191 2	–	104511° 2
1948.006	10	51334.54	.000	47978 2	–	99313° 1	1987.310	50	50319.27	.003	91674° 2	–	141993 3
					–					−.005	34225 4	–	84544° 4
1948.352	1h	51325.42	.001	91387° 4	–	142712 5						–	
1948.671	5	51317.02	−.001	49541 4	–	100858° 4	1988.056	80	50300.39	.007	44655 4	–	94955° 4
1948.824	10	51312.99	−.002	84544° 4	–	135857 4	1988.282	8	50294.67	.004	93102° 4	–	143396 3
1948.974	5	51309.04	.002	91387° 4	–	142696 4	1989.061	5	50274.97	.002	97709° 5	–	147984 6
			−.004	49088 2	–	100397° 3	1989.651	2	50260.07	.001	64331 3	–	114591° 3
1950.662	1	51264.64	.000	80343° 5	–	131607 5	1990.190	10	50246.45	.000	97184° 4	–	147431 4
1952.221	50	51223.70	.000	83147° 5	–	134371 5	1990.579	8	50236.64	.006	42521 2	–	92758° 3
1952.549	50	51215.10	.000	43461 3	–	94676° 3				.005	46601 4	–	96838° 3
1953.082	50	51201.12	.002	44655 4	–	95856° 3	1991.133	10	50222.66	.000	46962 5	–	97184° 4
1953.239	50	51197.01	.003	32387 2	–	83584° 3				−.004	93306° 6	–	143528 7
					–		1993.917	200	50152.53	.006	32387 2	–	82540° 2
1994.724	50	50132.24	.002	33452 3	–	83584° 3	2034.155	5	49144.65	.000	55366 1	–	104511° 2
			.000	85308° 1	–	135441 2	2034.300	5	49141.15	.003	98562° 3	–	147703 4
1995.151	20	50121.52	−.002	32418 1	–	82540° 2	2035.809	2	49104.73	−.006	94424° 7	–	143528 7
1995.560	1	50111.24	−.001	85329° 2	–	135441 2	2036.512	3	49087.78	.005	33452 3	–	82540° 2
1996.288	10	50092.97	−.005	42665 2	–	92758° 3	2036.548	5	49086.92	−.002	86892° 5	–	135979 6
1996.965	15	50075.99	−.005	106511° 4	–	156587 3	2039.299	20	49020.71	.000	49541 4	–	98562° 3
1997.534	20bl	50061.72	.001	56741 2	–	106803° 3	2039.558	1	49014.48	−.004	86426° 2	–	135441 2
1997.704	15	50057.46	−.004	96285° 5	–	146342 5	2039.800	50	49008.67	.004	42665 2	–	91674° 2
1998.277	1	50043.11	−.004	97709° 5	–	147752 5	2040.308	3	48996.47	−.005	96907° 6	–	145904 5
1999.145	50	50021.38	.003	44655 4	–	94676° 3	2041.335	5	48971.82	−.002	51425 3	–	100397° 3
2000.901	1	49961.30	−.002	85896° 4	–	135857 4	2041.620	3h	48964.99	−.006	86892° 5	–	135857 4
2001.242	30	49952.78	−.001	32587 3	–	82540° 2	2043.398	15	48922.39	.000	34225 4	–	83147° 5
2001.521	5	49945.82	.000	46962 5	–	96907° 6	2043.711	1	48914.89	−.004	51482 2	–	100397° 3
2001.826	1	49938.21	−.003	92884° 5	–	142822 6	2044.195	5	48903.31	−.001	85896° 4	–	134799 5
2003.878	3h	49887.08	−.006	83147° 5	–	133034 4	2046.184	5	48855.78	.003	82540° 2	–	131396 1
2004.688	5	49866.93	−.002	106511° 4	–	156378 4	2048.050	2	48811.28	.003	96285° 5	–	145096 4
2004.980	15	49859.67	.002	52697 4	–	102557° 3	2048.769	2	48794.15	−.004	51425 3	–	100219° 2
2005.068	3	49857.48	−.004	50362 1	–	100219° 2	2049.837	1	48768.73	.005	85896° 4	–	134665 3
2005.420	1	49848.73	−.004	93102° 4	–	142950 5	2050.290	10	48757.96	.001	47978 2	–	96736° 1
2006.222	2	49828.81	−.002	92884° 5	–	142712 5	2052.942	1	48694.98	.004	83584° 3	–	132279 3
2006.293	3	49827.04	−.003	84544° 4	–	134371 5	2054.702	20	48653.27	−.002	32387 2	–	81040° 3
2007.259	5h	49803.07	.002	85308° 1	–	135112 2	2055.096	5	48643.95	−.003	83584° 3	–	132228 2
2009.070	5	49758.18	−.004	85683° 1	–	135441 2	2055.355	100	48637.82	.005	43461 3	–	92099° 2
2009.219	1	49754.49	−.009	93642° 2	–	143396 3	2055.531	20	48633.66	.000	97709° 5	–	146342 5
2010.226	3	49729.57	−.003	88067 2	–	137796° 3	2056.331	1	48614.74	−.002	94098° 4	–	142712 5
2011.552	50	49696.79	.003	32843 2	–	82540° 2	2056.470	3	48611.45	−.002	47978 2	–	96589° 2
2011.633	100	49694.79	.002	42404 1	–	92099° 2	2056.647	15	48607.27	.002	84544° 4	–	133151 3
2011.894	1	49688.35	−.004	82540° 2	–	132228 2	2057.885	20	48578.03	−.006	42404 1	–	90982° 2
2012.078	5	49683.80	.000	46601 4	–	96285° 5	2058.289	3	48568.50	−.003	97709° 5	–	146277 6
2014.713	10	49618.83	.001	103485 4	–	153104° 3	2058.745	20	48557.74	−.003	33452 3	–	82009° 4
			.003	96285° 5	–	145904 5						–	
					–		2059.597	50	48537.66	.005	43561 3	–	92099° 2
2015.035	10	49610.90	.001	93102° 4	–	142712 5	2060.078	5	48526.33	.001	51425 3	–	99952° 2
			.004	32398 4	–	82009° 4	2061.254	5	48498.64	−.003	96285° 5	–	144783 6
2015.902	10	49589.57	.004	50362 1	–	99952° 2	2062.499	1	48469.37	.001	51482 2	–	99952° 2
2016.381	20	49577.79	−.003	42521 2	–	92099° 2	2062.862	5	48460.84	−.003	42521 2	–	90982° 2
2016.807	5	49567.32	−.001	83584° 3	–	133151 3	2063.179	10	48453.40	−.004	32587 3	–	81040° 3
2017.231	1	49556.91	−.002	46299 3	–	95856° 3	2064.458	15	48423.38	.000	54853 5	–	103276° 4
2017.905	3	49540.35	−.004	43561 3	–	93102° 4	2065.492	1	48399.15	.006	85896° 4	–	134295 3
2018.885	3	49516.31	−.004	93306° 6	–	142822 6	2066.697	3	48370.93	−.002	96285° 5	–	144656 4
2019.591	1	49499.00	−.003	86892° 5	–	136391 5	2066.787	1	48368.82	.005	86892° 5	–	135261 4
2020.621	8	49473.78	−.005	49088 2	–	98562° 3						–	
					–		2067.534	1	48351.35	.001	93642° 2	–	141993 3
2021.091	20	49462.27	.001	44655 4	–	94117° 3	2067.727	1	48346.84	−.001	82540° 2	–	130886 1
2021.882	1	49442.92	.002	44655 4	–	94098° 4	2068.493	8	48328.94	−.007	83584° 3	–	131913 2
2022.259	200	49433.71	.003	42665 2	–	92099° 2	2069.009	15	48316.89	−.003	42665 2	–	90982° 2
2022.716	10	49422.54	−.001	32587 3	–	82009° 4	2072.785	10	48228.88	.001	44655 4	–	92884° 5
2023.025	100	49414.99	.003	35129 5	–	84544° 4	2073.479	100	48212.74	.008	43461 3	–	91674° 2
2023.423	1	49405.27	.002	94424° 7	–	143829 6	2074.135	5	48197.49	−.004	32843 2	–	81040° 3
2024.888	1	49369.54	.002	96907° 6	–	146277 6	2074.238	10	48195.10	.004	97709° 5	–	145904 5
2025.060	1	49365.34	.001	85896° 4	–	135261 4	2074.829	3	48181.37	−.004	42404 1	–	90586° 2
2025.253	5	49360.64	.001	82540° 2	–	131900 1	2075.429	15	48167.44	−.002	49541 4	–	97709° 5
2025.316	5	49359.10	.002	34225 4	–	83584° 3						–	
					–		2075.717	1	48160.76	−.002	52697 4	–	100858° 4
2026.788	10	49323.26	.001	46962 5	–	96285° 5	2077.362	2	48122.63	−.003	89482 3	–	137605° 4
2027.806	1	49298.50	.001	94098° 4	–	143396 3	2077.785	3	48112.83	−.002	43561 3	–	91674° 2
2027.866	5	49297.04	−.002	43461 3	–	92758° 3	2078.200	50	48103.23	.004	44655 4	–	92758° 3
2028.984	20	49269.88	−.002	42404 1	–	91674° 2	2078.806	5	48089.21	−.002	82540° 2	–	130629 1
2029.603	20	49254.86	.000	46601 4	–	95856° 3	2079.413	3	48075.17	−.001	46601 4	–	94676° 3
2030.204	15	49240.28	.000	95856° 3	–	145096 4				.007	88499° 3	–	136574 4
2031.997	10	49196.84	.000	43561 3	–	92758° 3	2079.500	10	48073.16	.000	58730 4	–	106803° 3
2033.276	1	49165.90	−.004	94955° 4	–	144121 5	2079.783	20	48066.62	.000	42521 2	–	90588° 3
2033.600	2	49158.06	−.004	97184° 4	–	146342 5	2079.889	10	48064.17	.000	42521 2	–	90586° 2
2033.825	30	49152.63	.004	42521 2	–	91674° 2						–	
2081.879	300	48018.23	.003	35129 5	–	83147° 5	2123.390	10	47079.62	−.005	51482 2	–	98562° 3
2082.578	20	48002.12	.000	48734 1	–	96736° 1	2123.476	20	47077.71	.000	72481 1	–	119559° 0
2082.946	80	47993.64	.005	46962 5	–	94955° 4	2123.606	5	47074.83	.002	97709° 5	–	144783 6
2084.092	1	47967.25	−.009	32387 2	–	80354° 3	2124.126	5	47063.31	.001	84544° 4	–	131607 5
2084.460	1	47958.78	.001	103621° 5	–	151580 5	2124.763	1	47049.20	.002	32418 1	–	79467° 2
2084.605	1	47955.45	.006	32398 4	–	80354° 3	2125.280	1	47037.76	.004	47978 2	–	95016° 1
2084.838	1	47950.09	−.008	88441° 6	–	136391 5	2125.777	3	47026.76	−.003	43561 3	–	90588° 3
2085.088	10	47944.34	−.002	32398 4	–	80343° 5	2127.727	10	46983.67	.003	36164 4	–	83147° 5
2085.384	5	47937.54	−.001	55366 1	–	103303° 2	2129.385	8	46947.09	.004	97709° 5	–	144656 4
2085.886	1	47926.00	−.005	43461 3	–	91387° 4	2130.362	3	46925.56	−.002	56741 2	–	103667° 3
2086.028	20	47922.74	−.002	42665 2	–	90588° 3	2130.569	200	46921.00	−.002	32587 3	–	79508° 3
2086.139	10	47920.19	.002	42665 2	–	90586° 2	2131.077	5	46909.82	−.004	32587 3	–	79497° 4
2086.593	5	47909.76	.002	58893 3	–	106803° 3	2131.420	50	46902.27	−.001	33452 3	–	80354° 3
2087.144	3	47897.12	.000	42404 1	–	90301° 1	2131.592	1	46898.48	−.004	85329° 2	–	132228 2
2087.975	10	47878.06	−.004	47978 2	–	95856° 3	2132.078	5h	46887.80	−.004	89503° 4	–	136391 5
2088.061	5	47876.09	−.002	96907° 6	–	144783 6	2132.406	10	46880.58	−.001	32587 3	–	79467° 2
2088.960	50	47855.48	.003	48734 1	–	96589° 2	2133.071	200	46865.97	−.001	50318 5	–	97184° 4
2090.060	5	47830.30	−.007	51482 2	–	99313° 1	2133.810	50	46849.74	.000	72356 2	–	119206° 1
2090.263	10	47825.66	−.008	82540° *2*	–	130365 3	2135.279	15	46817.51	−.002	48734 1	–	95551° 2
			.003	43561 3	–	91387° 4	2135.965	100	46802.48	−.002	46299 3	–	93102° 4
			.006	85683° 1	–	133508 0						–	
					–		2136.378	50	46793.43	.000	43461 3	–	90255° 4
2090.597	5	47818.02	−.001	46299 3	–	94117° 3	2137.114	1	46777.32	−.003	53407 0	–	100184° 1
			−.004	96838° 3	–	144656 4	2137.310	80	46773.03	−.001	50362 1	–	97135° 0
2091.442	10	47798.70	−.001	46299 3	–	94098° 4	2137.550	30	46767.78	−.003	49088 2	–	95856° 3
2091.621	1	47794.61	.002	83584° 3	–	131379 2	2137.792	1	46762.49	−.006	88499° 3	–	135261 4
2091.671	3	47793.47	−.001	84544° 4	–	132337 3	2138.650	100	46743.73	−.001	49541 4	–	96285° 5
2092.064	100	47784.49	.001	34225 4	–	82009° 4	2139.176	10	46732.24	−.001	44655 4	–	91387° 4
2092.201	5	47781.36	.005	58730 4	–	106511° 4	2139.536	50	46724.37	.000	72481 1	–	119206° 1
2092.731	3	47769.26	−.002	56741 2	–	104511° 2	2140.734	15	46698.23	.001	47978 2	–	94676° 3
2092.826	1	47767.09	.001	32587 3	–	80354° 3	2140.963	150	46693.23	.002	43561 3	–	90255° 4
2093.589	10	47749.69	−.004	49088 2	–	96838° 3						–	
					–		2142.249	300	46665.21	.003	96285° 5	–	142950 5
2093.835	5	47744.08	−.002	59059 2	–	106803° 3				−.007	32843 2	–	79508° 3
2095.915	15	47696.70	.001	32398 4	–	80095° 4	2142.301	100	46664.07	−.002	77557 2	–	124221° 1
2098.282	50	47642.90	.008	49541 4	–	97184° 4	2143.253	200	46643.35	.001	33452 3	–	80095° 4
2098.582	5	47636.09	−.002	42665 2	–	90301° 1	2144.026	50	46626.53	.000	32387 2	–	79013° 2
2099.375	10	47618.10	.001	58893 3	–	106511° 4	2144.113	100	46624.64	.000	32843 2	–	79467° 2
2100.685	100	47588.41	.002	33452 3	–	81040° 3	2144.652	1	46612.93	−.004	88499° 3	–	135112 2
2102.647	5	47544.01	.000	96285° 5	–	143829 6	2145.464	200	46595.29	.000	32418 1	–	79013° 2
2103.667	50	47520.96	.001	43461 3	–	90982° 2	2145.749	50	46589.10	.000	50318 5	–	96907° 6
2103.889	50	47515.95	.002	46601 4	–	94117° 3	2146.992	30	46562.13	−.002	56741 2	–	103303° 2
2104.098	1	47511.23	−.001	32843 2	–	80354° 3						–	
					–		2147.079	3	46560.24	.003	32387 2	–	78947° 1
2104.234	20	47508.16	.003	32587 3	–	80095° 4	2148.518	10	46529.06	.001	32418 1	–	78947° 1
2104.542	100	47501.21	−.002	49088 2	–	96589° 2	2149.560	1	46506.51	.004	91098 4	–	137605° 4
2104.746	20	47496.60	.003	46601 4	–	94098° 4	2149.844	30	46500.37	.003	46601 4	–	93102° 4
2105.110	30h	47488.39	.006	43561 3	–	91050° 3	2150.108	10	46494.66	−.001	32519 1	–	79013° 2
2108.110	20	47420.82	.001	43561 3	–	90982° 2	2151.062	20	46474.04	.000	42665 2	–	89139° 1
2109.470	50	47390.25	.000	50318 5	–	97709° 5	2151.308	1	46468.72	.000	97184° 4	–	143653 5
2109.590	1	47387.56	.004	97709° 5	–	145096 4	2151.568	1	46463.11	−.002	49088 2	–	95551° 2
2110.321	10	47371.14	−.002	42404 1	–	89775° 0	2151.752	1	46459.14	−.005	46299 3	–	92758° 3
2111.580	100	47342.90	.002	46299 3	–	93642° 2	2153.277	50	46426.24	.003	50481 6	–	96907° 6
2113.644	200	47296.68	.000	49541 4	–	96838° 3						–	
					–		2154.063	10	46409.30	−.003	47978 2	–	94387° 2
2113.872	8	47291.58	.001	72187 3	–	119479° 3	2154.728	30	46394.98	.002	44655 4	–	91050° 3
2113.896	8bl	47291.04	−.010	88592° 2	–	135882 3	2155.475	1	46378.90	.007	89503° 4	–	135882 3
2116.743	200	47227.44	.001	50481 6	–	97709° 5	2155.706	5	46373.93	−.003	50362 1	–	96736° 1
2120.616	20	47141.20	.000	52811 1	–	99952° 2	2156.364	1	46359.78	−.004	96838° 3	–	143198 4
2120.837	30	47136.28	−.006	46962 5	–	94098° 4	2156.743	200	46351.64	−.001	44655 4	–	91006° 5
2121.260	3	47126.89	−.002	43461 3	–	90588° 3	2157.098	100	46344.01	−.001	46962 5	–	93306° 6
2121.372	8	47124.40	−.001	43461 3	–	90586° 2	2158.463	80	46314.70	.003	49541 4	–	95856° 3
2121.431	10	47123.09	−.001	72356 2	–	119479° 3	2159.635	5	46289.57	.001	89689° 5	–	135979 6
2121.528	20	47120.93	−.003	32387 2	–	79508° 3	2159.960	10bl	46282.61	−.001	46601 4	–	92884° 5
2122.548	1	47098.29	−.004	32398 4	–	79497° 4						–	
2159.986	100bl	46282.05	−.003	48734 1	–	95016° 1	2194.302	20	45558.34	−.001	48734 1	–	94292° 1
2160.781	10	46265.03	.000	42404 1	–	88669° 0	2195.263	300	45538.40	.000	43561 3	–	89100° 4
2160.861	80	46263.31	−.001	72187 3	–	118451° 2	2196.034	5	45522.41	.006	33155 0	–	78677° 1
2161.051	300	46259.25	.000	32418 1	–	78677° 1	2199.457	50	45451.57	−.003	59059 2	–	104511° 2
2162.545	20	46227.29	.001	50362 1	–	96589° 2	2200.669	200	45426.54	−.003	42404 1	–	87831° 1
2164.413	50	46187.40	−.002	42404 1	–	88592° 2	2201.352	40	45412.45	−.001	51425 3	–	96838° 3
2165.197	400	46170.68	.001	32843 2	–	79013° 2	2202.697	3	45384.72	−.003	91006° 5	–	136391 5
2165.346	10	46167.50	.002	89689° 5	–	135857 4	2203.171	50	45374.96	.000	46299 3	–	91674° 2
2165.637	1	46161.30	.004	89100° 4	–	135261 4	2204.813	1	45341.17	.000	87810° 3	–	133151 3
2165.760	20	46158.68	−.004	32519 1	–	78677° 1	2206.079	300	45315.15	−.001	32843 2	–	78158° 3
2166.184	200	46149.64	.002	32418 1	–	78568° 0	2206.360	5	45309.38	−.001	42521 2	–	87831° 1
2166.643	80	46139.87	.002	46962 5	–	93102° 4	2206.870	200	45298.91	.003	49088 2	–	94387° 2
2166.679	20	46139.10	.000	47978 2	–	94117° 3	2207.027	1	45295.69	−.002	89503° 4	–	134799 5
2167.146	300	46129.16	−.002	34225 4	–	80354° 3	2207.362	400	45288.82	.000	42521 2	–	87810° 3
2167.676	300	46117.88	−.004	34225 4	–	80343° 5	2207.646	100	45282.99	−.002	34225 4	–	79508° 3
2168.306	200	46104.48	.000	32843 2	–	78947° 1	2208.194	300	45271.75	−.002	34225 4	–	79497° 4
2168.764	50	46094.75	.000	72356 2	–	118451° 2	2209.082	200	45253.56	.001	51482 2	–	96736° 1
2169.130	1	46086.97	.004	85308° 1	–	131396 1	2209.745	10	45239.98	−.001	88499° 3	–	133739 4
2169.919	50	46070.21	.002	85308° 1	–	131379 2	2211.019	500	45213.92	−.009	35129 5	–	80343° 5
			.001	42521 2	–	88592° 2	2212.227	30	45189.23	−.001	50362 1	–	95551° 2
2170.584	500	46056.10	.000	33452 3	–	79508° 3	2213.396	8	45165.37	.003	42665 2	–	87831° 1
2170.918	8	46049.02	.000	32519 1	–	78568° 0	2213.461	100	45164.04	−.003	51425 3	–	96589° 2
2171.116	200	46044.82	.002	33452 3	–	79497° 4	2213.602	1	45161.16	.002	89503° 4	–	134665 3
2171.237	10	46042.25	−.001	43461 3	–	89503° 4	2213.872	1	45155.66	−.005	96838° 3	–	141993 3
2171.404	1	46038.71	.005	96907° 6	–	142946 5	2214.401	500	45144.87	.001	42665 2	–	87810° 3
2171.874	1	46028.75	−.001	54191 2	–	100219° 2	2214.883	3	45135.05	.000	49541 4	–	94676° 3
2172.487	400	46015.76	−.003	33452 3	–	79467° 2	2215.533	200	45121.81	−.001	54191 2	–	99313° 1
2172.981	15	46005.30	.001	54853 5	–	100858° 4	2216.612	200	45099.84	.001	72187 3	–	117287° 4
2173.545	30	45993.37	.002	54191 2	–	100184° 1	2217.197	200	45087.95	−.001	46299 3	–	91387° 4
2174.100	100	45981.63	.001	59059 2	–	105041° 1	2218.146	400	45068.66	−.004	42404 1	–	87473° 2
2174.301	50	45977.38	.000	42521 2	–	88499° 3	2218.369	5	45064.13	.003	88499° 3	–	133563 2
2174.680	10	45969.36	.001	72481 1	–	118451° 2	2219.677	10	45037.57	.001	43461 3	–	88499° 3
2174.811	80	45966.60	−.002	50318 5	–	96285° 5	2219.823	100	45034.61	.001	44655 4	–	89689° 5
2175.970	60	45942.11	−.001	43561 3	–	89503° 4	2220.105	20	45028.89	−.002	49088 2	–	94117° 3
2176.399	2	45933.06	.005	44655 4	–	90588° 3	2220.978	200	45011.20	−.004	52697 4	–	97709° 5
2176.663	3	45927.49	.005	49088 2	–	95016° 1	2221.334	5	45003.98	.004	91387° 4	–	136391 5
2176.723	3	45926.22	.003	42665 2	–	88592° 2				−.006	97709° 5	–	142712 5
2176.923	60	45922.00	.003	46962 5	–	92884° 5	2222.757	50	44975.17	−.002	48734 1	–	93709° 1
2177.437	1	45911.17	−.001	86426° 2	–	132337 3	2222.885	10	44972.58	.001	91006° 5	–	135979 6
2177.700	30	45905.62	.000	53407 0	–	99313° 1	2222.949	1	44971.29	.002	88592° 2	–	133563 2
2179.383	400	45870.18	.002	34225 4	–	80095° 4	2223.201	300	44966.19	−.002	35129 5	–	80095° 4
2179.654	100	45864.47	−.003	52697 4	–	98562° 3	2223.928	20	44951.49	−.001	42521 2	–	87473° 2
2179.951	3	45858.22	.001	96838° 3	–	142696 4	2224.649	200	44936.93	.000	58730 4	–	103667° 3
2181.073	10	45834.64	.001	32843 2	–	78677° 1	2226.927	400	44890.96	−.002	58730 4	–	103621° 5
2181.129	100	45833.46	−.001	42665 2	–	88499° 3	2227.643	1	44876.54	.006	36164 4	–	81040° 3
2182.547	300	45803.69	.003	50481 6	–	96285° 5	2227.966	1	44870.03	−.004	42521 2	–	87391° 3
2182.722	30	45800.01	−.002	46299 3	–	92099° 2	2228.717	30	44854.91	.000	72481 1	–	117336° 2
2184.303	500	45766.87	−.001	46962 5	–	92728° 6				−.001	48854 0	–	93709° 1
2184.693	10	45758.70	.008	51425 3	–	97184° 4	2228.789	10	44853.46	.002	55366 1	–	100219° 2
2186.018	10	45730.96	.001	47978 2	–	93709° 1	2228.932	15	44850.59	−.002	91006° 5	–	135857 4
2187.337	1	45703.39	.002	88592° 2	–	134295 3	2229.036	15	44848.50	.003	44655 4	–	89503° 4
2188.537	1	45678.33	.009	87473° 2	–	133151 3	2229.741	1	44834.32	.005	98562° 3	–	143396 3
2189.749	100	45653.05	−.006	46601 4	–	92254° 5	2230.545	200	44818.16	.001	55366 1	–	100184° 1
2190.446	500	45638.53	.001	43461 3	–	89100° 4	2232.152	100	44785.89	.001	46601 4	–	91387° 4
2190.956	5	45627.91	−.003	90255° 4	–	135882 3	2232.767	80	44773.56	.001	58893 3	–	103667° 3
2191.468	200	45617.25	.002	58893 3	–	104511° 2	2233.910	50	44750.65	.003	46299 3	–	91050° 3
2192.316	3	45599.60	.008	44655 4	–	90255° 4	2235.139	20	44726.05	−.002	42665 2	–	87391° 3
2192.875	20	45587.98	.001	49088 2	–	94676° 3	2235.723	3	44714.37	.001	32398 4	–	77113° 5
2193.691	3	45571.03	.001	32587 3	–	78158° 3	2236.132	100	44706.19	.000	33452 3	–	78158° 3
2193.855	2	45567.62	.001	91006° 5	–	136574 4	2237.787	1	44673.13	−.002	90588° 3	–	135261 4
2238.758	30	44653.76	−.001	50362 1	–	95016° 1	2276.942	30	43904.99	−.001	42521 2	–	86426° 2
2240.419	8	44620.65	.004	49088 2	–	93709° 1	2278.079	1	43883.08	.005	90301° 1	–	134185 1
2241.065	50	44607.79	.000	59059 2	–	103667° 3	2278.544	2	43874.12	−.003	91387° 4	–	135261 4
2242.171	100	44585.79	.000	55366 1	–	99952° 2	2280.117	10	43843.86	.004	44655 4	–	88499° 3
2243.483	1	44559.72	.002	88592° 2	–	133151 3	2280.835	150	43830.06	−.002	43561 3	–	87391° 3
2243.643	100	44556.54	.002	49541 4	–	94098° 4	2280.987	50	43827.14	.003	58730 4	–	102557° 3
2243.784	5	44553.74	.002	49088 2	–	93642° 2	2282.782	2	43792.68	−.004	91006° 5	–	134799 5
2244.163	15	44546.22	.002	58730 4	–	103276° 4	2283.478	5	43779.33	.005	50318 5	–	94098° 4
2244.250	10	44544.49	−.002	90255° 4	–	134799 5	2284.433	100	43761.03	.000	42665 2	–	86426° 2
2245.306	1	44523.54	.003	90588° 3	–	135112 2	2286.316	1	43724.99	−.001	92254° 5	–	135979 6
2247.100	100	44488.00	.002	48734 1	–	93222° 1	2287.128	2	43709.47	.001	90586° 2	–	134295 3
2247.163	80	44486.75	.003	52697 4	–	97184° 4	2287.264	1	43706.87	.009	90588° 3	–	134295 3
2247.743	1	44475.28	.004	103276° 4	–	147752 5	2287.830	1	43696.06	.001	47978 2	–	91674° 2
2249.090	200	44448.64	.004	46601 4	–	91050° 3	2289.200	200	43669.91	−.002	49088 2	–	92758° 3
2249.283	15	44444.83	.003	44655 4	–	89100° 4	2289.516	1	43663.88	−.002	58893 3	–	102557° 3
2250.005	200	44430.57	−.001	51425 3	–	95856° 3	2290.065	200	43653.42	.003	46601 4	–	90255° 4
2250.268	100	44425.38	.001	46962 5	–	91387° 4	2292.101	2	43614.65	.006	91050° 3	–	134665 3
2250.636	60	44418.11	.001	32418 1	–	76836° 2	2292.559	3	43605.93	.008	72187 3	–	115794° 2
2251.041	10	44410.12	.002	58893 3	–	103303° 2	2293.532	50	43587.44	−.001	52697 4	–	96285° 5
			−.006	90255° 4	–	134665 3	2293.672	20	43584.78	.007	32387 2	–	75972° 1
2251.288	100	44405.25	.003	46601 4	–	91006° 5	2294.974	600	43560.05	.000	35129 5	–	78689° 6
2252.420	200	44382.94	−.001	58893 3	–	103276° 4	2295.310	50	43553.67	.000	32418 1	–	75972° 1
2252.894	50	44373.60	−.001	51482 2	–	95856° 3	2296.375	20	43533.48	−.002	51482 2	–	95016° 1
2253.195	300	44367.67	−.002	35129 5	–	79497° 4	2296.558	200	43530.01	−.003	51425 3	–	94955° 4
			.006	48854 0	–	93222° 1	2298.243	200	43498.10	−.002	59059 2	–	102557° 3
2253.445	1	44362.75	−.003	103621° 5	–	147984 6	2299.300	50	43478.10	−.001	56741 2	–	100219° 2
2254.138	10	44349.11	−.004	43461 3	–	87810° 3	2300.629	3	43452.99	.001	32519 1	–	75972° 1
2255.389	30	44324.51	.001	52811 1	–	97135° 0	2301.171	20	43442.75	.000	56741 2	–	100184° 1
2257.202	200	44288.92	−.002	46299 3	–	90588° 3	2301.446	10	43437.56	−.001	72356 2	–	115794° 2
2257.332	100	44286.37	.003	46299 3	–	90586° 2				−.003	91674° 2	–	135112 2
2258.952	1	44254.61	.002	91006° 5	–	135261 4	2301.766	1	43431.53	−.001	98562° 3	–	141993 3
2259.218	20	44249.40	.002	32587 3	–	76836° 2	2302.502	8	43417.64	−.002	32398 4	–	75816° 4
2259.251	20	44248.75	.007	43561 3	–	87810° 3	2304.255	200	43384.62	−.002	33452 3	–	76836° 2
2259.477	30	44244.33	.002	59059 2	–	103303° 2	2305.278	50	43365.37	−.008	48734 1	–	92099° 2
2259.935	1	44235.36	−.002	89503° 4	–	133739 4	2306.257	50	43346.96	−.004	50362 1	–	93709° 1
2261.162	2	44211.36	−.005	91050° 3	–	135261 4	2306.493	200	43342.52	−.001	49541 4	–	92884° 5
2262.818	1	44179.01	.008	36164 4	–	80343° 5	2307.210	10	43329.06	−.001	53407 0	–	96736° 1
2264.070	1	44154.58	−.005	103276° 4	–	147431 4	2308.109	60	43312.18	.000	72481 1	–	115794° 2
2264.735	100	44141.62	−.007	72356 2	–	116497° 3	2309.823	100	43280.04	−.006	50362 1	–	93642° 2
2264.798	50	44140.39	−.001	52697 4	–	96838° 3	2309.905	100	43278.51	−.006	42404 1	–	85683° 1
2265.150	30	44133.53	.005	49088 2	–	93222° 1	2310.800	1	43261.75	−.006	87810° 3	–	131072 2
2265.543	20	44125.87	.001	51425 3	–	95551° 2	2311.381	20	43250.87	−.003	51425 3	–	94676° 3
2265.786	1	44121.14	−.003	47978 2	–	92099° 2	2311.682	1	43245.24	−.002	91050° 3	–	134295 3
2268.070	1	44076.72	−.006	90588° 3	–	134665 3	2312.539	10	43229.22	−.004	32587 3	–	75816° 4
2268.468	100	44068.98	−.003	51482 2	–	95551° 2	2313.194	1	43216.98	−.002	49541 4	–	92758° 3
2269.361	2	44051.64	.001	89100° 4	–	133151 3	2313.545	50	43210.42	−.003	56741 2	–	99952° 2
2269.715	300	44044.77	.001	46962 5	–	91006° 5	2313.718	1	43207.19	−.007	90301° 1	–	133508 0
2270.728	50	44025.13	−.001	50362 1	–	94387° 2	2313.868	50	43204.39	−.007	46299 3	–	89503° 4
2270.883	50	44022.12	−.001	42404 1	–	86426° 2	2314.428	100	43193.94	−.004	51482 2	–	94676° 3
2272.362	50	43993.47	.003	32843 2	–	76836° 2	2316.175	30	43161.36	−.005	42521 2	–	85683° 1
2272.703	20	43986.87	.000	46601 4	–	90588° 3	2316.327	5	43158.53	−.002	52697 4	–	95856° 3
2274.334	15bl	43955.33	.007	46299 3	–	90255° 4	2316.498	10	43155.34	.002	44655 4	–	87810° 3
2274.792	15	43946.48	.003	55366 1	–	99313° 1	2316.745	3	43150.74	−.002	90588° 3	–	133739 4
2275.002	400	43942.42	.001	50481 6	–	94424° 7	2317.912	100	43129.02	.003	32843 2	–	75972° 1
2275.487	200	43933.06	−.001	34225 4	–	78158° 3	2320.100	50	43088.35	−.001	46601 4	–	89689° 5
2275.640	200	43930.11	.003	43461 3	–	87391° 3	2320.989	8	43071.85	−.001	47978 2	–	91050° 3
			−.002	50362 1	–	94292° 1	2323.928	100	43017.38	−.002	42665 2	–	85683° 1
2275.883	10	43925.42	−.002	52811 1	–	96736° 1	2325.552	100	42987.34	−.004	50318 5	–	93306° 6
2276.270	60	43917.95	.000	42404 1	–	86322° 0	2326.088	1	42977.44	−.002	90586° 2	–	133563 2
2276.603	10	43911.53	.001	43561 3	–	87473° 2	2326.226	5	42974.89	.003	90588° 3	–	133563 2
2326.308	1	42973.37	−.005	92884° 5	–	135857 4	2377.137	300	42054.57	.000	54853 5	–	96907° 6
2326.753	50	42965.15	.002	43461 3	–	86426° 2	2381.145	200	41983.79	.001	35129 5	–	77113° 5
2326.936	20	42961.78	.001	51425 3	–	94387° 2	2381.431	10	41978.75	.001	52697 4	–	94676° 3
2328.104	40	42940.22	.000	48734 1	–	91674° 2	2382.221	15	41964.83	.001	58893 3	–	100858° 4
2328.911	20	42925.35	−.004	42404 1	–	85329° 2	2382.408	50	41961.54	.002	49088 2	–	91050° 3
2330.016	10	42904.99	−.009	51482 2	–	94387° 2	2383.877	150	41935.68	.001	50318 5	–	92254° 5
2330.055	30	42904.27	−.003	42404 1	–	85308° 1	2384.650	100	41922.09	.001	77557 2	–	119479° 3
2330.165	10	42902.25	.001	46601 4	–	89503° 4	2386.051	100	41897.47	.009	88067 2	–	129964° 3
2330.945	100	42887.89	.000	34225 4	–	77113° 5				.009	46601 4	–	88499° 3
2332.188	20	42865.04	.001	43561 3	–	86426° 2	2386.185	20	41895.12	.000	72187 3	–	114083° 2
2332.476	20	42859.74	.002	50362 1	–	93222° 1	2386.260	50	41893.80	.003	49088 2	–	90982° 2
2332.687	50	42855.87	−.007	54853 5	–	97709° 5	2386.969	500	41881.36	.004	32843 2	–	74724° 3
2334.398	20	42824.46	.001	5048! 6	–	93306° 6	2387.709	1	41868.38	−.001	43461 3	–	85329° 2
2334.814	3	42816.83	.007	33155 0	–	75972° 1	2388.660	30	41851.72	−.003	48734 1	–	90586° 2
2335.194	5	42809.86	−.004	51482 2	–	94292° 1	2388.997	200	41845.81	.001	49541 4	–	91387° 4
2335.286	3	42808.18	−.001	42521 2	–	85329° 2	2390.444	100	41820.48	.000	56741 2	–	98562° 3
2336.439	50	42787.05	.003	42521 2	–	85308° 1	2390.948	50	41811.67	.002	42404 1	–	84216° 0
			.004	88592° 2	–	131379 2	2393.176	50	41772.75	.009	50481 6	–	92254° 5
2336.646	20	42783.26	−.004	50318 5	–	93102° 4	2393.442	20	41768.10	.007	43561 3	–	85329° 2
2338.969	40	42740.77	−.002	52811 1	–	95551° 2	2393.604	3	41765.28	.002	94117° 3	–	135882 3
2339.205	3	42736.46	.002	44655 4	–	87391° 3	2395.227	20	41736.98	−.001	50362 1	–	92099° 2
2339.305	1	42734.64	−.008	89689° 5	–	132424 6	2395.824	50	41726.58	.000	72356 2	–	114083° 2
2339.680	30	42727.79	.001	46962 5	–	89689° 5	2399.242	40	41667.14	.003	58730 4	–	100397° 3
2340.499	20	42712.84	.000	49541 4	–	92254° 5	2399.354	100	41665.19	.001	54191 2	–	95856° 3
2341.660	50	42691.66	.001	51425 3	–	94117° 3	2399.756	30	41658.22	.003	72356 2	–	114014° 1
2342.720	10	42672.35	.000	51425 3	–	94098° 4	2402.041	5	41618.59	.005	94955° 4	–	136574 4
2343.168	80	42664.19	.001	42665 2	–	85329° 2	2402.603	80	41608.86	.003	53407 0	–	95016° 1
2344.102	5	42647.19	−.005	54191 2	–	96838° 3	2403.043	2	41601.24	−.001	72481 1	–	114083° 2
2344.327	50	42643.10	.004	42665 2	–	85308° 1	2403.627	200	41591.13	.000	34225 4	–	75816° 4
2344.788	50	42634.72	−.001	51482 2	–	94117° 3	2404.468	30	41576.58	.003	52811 1	–	94387° 2
2347.481	20	42585.81	.000	49088 2	–	91674° 2	2404.995	50	41567.47	.001	48734 1	–	90301° 1
2348.292	1	42571.10	.001	56741 2	–	99313° 1				−.003	72481 1	–	114014° 1
2348.608	50	42565.38	−.001	50318 5	–	92884° 5	2408.269	1	41510.97	.006	46299 3	–	87810° 3
			.007	92099° 2	–	134665 3	2408.410	20	41508.54	.005	49541 4	–	91050° 3
2349.732	3h	42545.02	−.002	92254° 5	–	134799 5	2408.684	100	41503.82	.002	58893 3	–	100397° 3
2349.911	100	42541.78	−.002	46962 5	–	89503° 4	2409.072	2h	41497.13	.008	49088 2	–	90586° 2
2355.845	1	42434.63	−.003	43461 3	–	85896° 4	2409.971	50	41481.65	−.004	52811 1	–	94292° 1
2357.204	20	42410.17	−.002	50318 5	–	92728° 6	2410.101	300	41479.42	.007	46962 5	–	88441° 6
2357.582	50	42403.37	−.002	72187 3	–	114591° 3	2410.342	1	41475.27	.004	103621° 5	–	145096 4
2357.634	15	42402.43	.007	50481 6	–	92884° 5	2410.676	5	41469.52	.001	93642° 2	–	135112 2
2357.844	40	42398.65	−.001	54191 2	–	96589° 2	2410.925	100	41465.24	−.001	49541 4	–	91006° 5
2359.760	300	42364.23	.002	33452 3	–	75816° 4	2412.718	300	41434.43	.004	32418 1	–	73853° 2
2360.463	1	42351.62	.003	91387° 4	–	133739 4	2412.856	100	41432.06	−.001	54853 5	–	96285° 5
2361.260	10	42337.32	−.003	32387 2	–	74724° 3	2413.581	1	41419.62	.000	52697 4	–	94117° 3
2361.419	3	42334.47	−.002	43561 3	–	85896° 4	2414.709	100	41400.27	.002	52697 4	–	94098° 4
2361.590	5	42331.41	.001	54853 5	–	97184° 4	2418.342	1	41338.08	−.001	59059 2	–	100397° 3
2362.042	1	42323.31	.003	47978 2	–	90301° 1	2418.587	50	41333.89	−.004	32519 1	–	73853° 2
2363.105	1	42304.27	−.006	106511° 4	–	148816 5	2418.657	20	41332.70	.001	51425 3	–	92758° 3
2363.766	30	42292.44	.002	46299 3	–	88592° 2	2419.872	80	41311.94	.001	50362 1	–	91674° 2
2365.696	2	42257.94	−.003	52697 4	–	94955° 4	2422.190	300	41272.41	.004	33452 3	–	74724° 3
2366.290	200	42247.33	.000	50481 6	–	92728° 6	2422.578	1	41265.80	.000	32587 3	–	73853° 2
2366.850	10	42237.34	.000	44655 4	–	86892° 5	2425.068	30	41223.43	−.001	55366 1	–	96589° 2
2367.884	1	42218.90	−.004	93222° 1	–	135441 2	2425.678	50	41213.07	.002	49088 2	–	90301° 1
2368.011	2	42216.63	−.001	51425 3	–	93642° 2	2425.919	5	41208.97	.006	46601 4	–	87810° 3
2368.650	15	42205.24	.001	52811 1	–	95016° 1	2428.725	50	41161.37	−.002	47978 2	–	89139° 1
2368.963	70	42199.67	−.002	46299 3	–	88499° 3	2428.783	100	41160.39	−.001	59059 2	–	100219° 2
2370.026	100	42180.74	.001	64331 3	–	106511° 4	2430.875	1	41124.97	.005	59059 2	–	100184° 1
2371.205	30	42159.77	−.007	51482 2	–	93642° 2	2433.363	2	41082.92	−.002	43461 3	–	84544° 4
2372.468	15	42137.33	.001	32587 3	–	74724° 3	2434.208	50	41068.66	.001	50318 5	–	91387° 4
2372.981	150	42128.22	−.001	58730 4	–	100858° 4	2434.560	30	41062.72	−.002	42521 2	–	83584° 3
2434.816	1	41058.41	.002	58893 3	–	99952° 2	2519.782	1	39674.03	−.008	103276° 4	–	142950 5
2435.114	1	41053.38	−.002	92728° 6	–	133782 5	2521.319	3	39649.85	.000	55366 1	–	95016° 1
2435.507	5	41046.76	.002	49541 4	–	90588° 3	2522.943	20	39624.33	.004	51425 3	–	91050° 3
2435.821	100	41041.47	.000	48734 1	–	89775° 0	2524.711	200	39596.58	.002	46299 3	–	85896° 4
2437.695	300	41009.92	−.001	32843 2	–	73853° 2	2525.804	1	39579.45	−.006	92758° 3	–	132337 3
2439.311	80	40982.75	.000	43561 3	–	84544° 4	2526.573	50	39567.40	.002	51482 2	–	91050° 3
2442.639	1	40926.92	.008	94955° 4	–	135882 3	2527.139	10	39558.54	−.003	49541 4	–	89100° 4
2443.125	30	40918.78	−.001	42665 2	–	83584° 3	2527.261	100	39556.63	.004	51425 3	–	90982° 2
2444.149	1h	40901.64	−.002	94955° 4	–	135857 4				−.005	52697 4	–	92254° 5
2444.341	30	40898.42	−.002	52811 1	–	93709° 1	2529.718	2	39518.21	.000	54191 2	–	93709° 1
2444.616	1	40893.82	−.001	77557 2	–	118451° 2	2530.668	30	39503.38	−.003	49088 2	–	88592° 2
2444.688	50	40892.62	.002	59059 2	–	99952° 2	2530.706	20	39502.78	−.001	59059 2	–	98562° 3
2445.126	15	40885.29	−.002	53407 0	–	94292° 1	2530.901	30	39499.74	−.001	51482 2	–	90982° 2
2448.351	60	40831.44	.001	52811 1	–	93642° 2	2531.214	3	39494.86	−.002	47978 2	–	87473° 2
2455.454	150	40713.34	.002	49541 4	–	90255° 4	2534.013	20	39451.24	.003	54191 2	–	93642° 2
2456.977	15	40688.10	−.002	50318 5	–	91006° 5	2536.633	10	39410.49	−.001	49088 2	–	88499° 3
2457.039	50	40687.07	−.002	35129 5	–	75816° 4	2539.174	30	39371.05	.002	50318 5	–	89689° 5
2457.854	70	40673.58	.004	51425 3	–	92099° 2	2541.418	8	39336.29	−.004	64331 3	–	103667° 3
2461.094	2	40620.04	−.002	50362 1	–	90982° 2	2544.112	100	39294.64	−.002	46601 4	–	85896° 4
2461.295	20	40616.72	−.003	51482 2	–	92099° 2	2545.772	2h	39269.02	.000	88067 2	–	127336° 2
2461.485	10	40613.59	.000	47978 2	–	88592° 2	2547.336	150	39244.91	.000	54853 5	–	94098° 4
2466.852	20	40525.24	.002	50481 6	–	91006° 5	2549.715	50	39208.30	−.001	50481 6	–	89689° 5
2468.431	80	40499.31	.001	34225 4	–	74724° 3	2551.227	15	39185.06	−.002	50318 5	–	89503° 4
2469.273	150	40485.51	−.001	54191 2	–	94676° 3	2552.694	20	39162.54	.001	51425 3	–	90588° 3
2473.813	10	40411.21	.006	52811 1	–	93222° 1	2552.857	10	39160.04	.003	51425 3	–	90586° 2
2474.163	50	40405.49	−.001	48734 1	–	89139° 1	2555.827	8	39114.54	.001	56741 2	–	95856° 3
2474.250	200	40404.07	−.002	52697 4	–	93102° 4	2556.412	15	39105.59	.000	51482 2	–	90588° 3
2474.448	60	40400.84	.006	33452 3	–	73853° 2	2556.578	30	39103.05	.005	51482 2	–	90586° 2
2474.913	1	40393.25	−.002	92758° 3	–	133151 3	2556.985	5	39096.83	.002	48734 1	–	87831° 1
2481.192	500	40291.04	.000	46601 4	–	86892° 5	2558.184	100	39078.50	.001	43461 3	–	82540° 2
2483.806	1	40248.64	.000	51425 3	–	91674° 2	2560.406	1	39044.59	−.003	96838° 3	–	135882 3
2485.361	20	40223.46	−.003	50362 1	–	90586° 2	2561.299	10	39030.98	.009	54191 2	–	93222° 1
2487.031	50	40196.45	.000	54191 2	–	94387° 2	2561.340	1	39030.36	.003	46299 3	–	85329° 2
2487.666	300	40186.19	.002	52697 4	–	92884° 5	2561.935	100	39021.29	−.004	55366 1	–	94387° 2
2489.880	1	40150.46	.003	92884° 5	–	133034 4	2564.755	200	38978.39	−.001	43561 3	–	82540° 2
2490.018	300	40148.23	.000	49541 4	–	89689° 5	2565.122	100	38972.81	−.001	64331 3	–	103303° 2
2491.588	200	40122.94	−.002	43461 3	–	83584° 3				−.005	92099° 2	–	131072 2
2492.634	3	40106.10	.005	96285° 5	–	136391 5	2566.120	80	38957.66	−.008	49541 4	–	88499° 3
2492.853	10	40102.58	−.005	54853 5	–	94955° 4	2566.919	3	38945.53	.002	64331 3	–	103276° 4
2492.927	5	40101.39	.002	54191 2	–	94292° 1	2567.251	10	38940.50	.002	77557 2	–	116497° 3
2493.237	50	40096.40	.003	56741 2	–	96838° 3	2567.676	20	38934.05	.003	46962 5	–	85896° 4
2495.464	15	40060.62	.001	52697 4	–	92758° 3	2567.992	100	38929.26	−.001	44655 4	–	83584° 3
2496.057	200	40051.10	−.002	49088 2	–	89139° 1	2568.200	3	38926.11	.005	55366 1	–	94292° 1
2497.827	300	40022.73	.002	43561 3	–	83584° 3	2572.170	3	38866.03	.007	91098 4	–	129964° 3
2498.104	200	40018.29	.001	42521 2	–	82540° 2	2572.341	8	38863.45	.002	52811 1	–	91674° 2
2499.585	30	39994.58	−.002	56741 2	–	96736° 1	2573.144	1	38851.32	−.001	96589° 2	–	135441 2
2501.606	100	39962.27	−.006	49541 4	–	89503° 4	2575.289	1	38818.96	−.001	51482 2	–	90301° 1
2501.650	50	39961.57	.002	51425 3	–	91387° 4	2575.899	1	38809.77	.009	56741 2	–	95551° 2
2503.050	2	39939.22	.002	50362 1	–	90301° 1	2578.062	50	38777.21	.001	50362 1	–	89139° 1
2503.237	50	39936.23	−.001	50318 5	–	90255° 4	2580.610	1	38738.93	.003	48734 1	–	87473° 2
2503.287	50	39935.44	−.002	48734 1	–	88669° 0	2581.751	2	38721.81	.008	49088 2	–	87810° 3
2503.595	200	39930.52	−.001	46962 5	–	86892° 5	2583.911	15	38689.44	.006	52697 4	–	91387° 4
2503.855	50	39926.38	−.003	54191 2	–	94117° 3	2584.385	3	38682.34	.009	97709° 5	–	136391 5
2504.298	3h	39919.32	.004	92254° 5	–	132173 4	2592.094	30	38567.31	.004	54191 2	–	92758° 3
2505.231	1	39904.45	−.007	90982° 2	–	130886 1	2593.366	200	38548.39	−.008	43461 3	–	82009° 4
2506.189	500	39889.20	.002	44655 4	–	84544° 4	2595.352	300	38518.90	−.003	42521 2	–	81040° 3
2507.121	100	39874.37	.000	42665 2	–	82540° 2				.003	93709° 1	–	132228 2
2508.165	100	39857.77	−.002	48734 1	–	88592° 2	2597.129	300	38492.54	.002	95016° 1	–	133508 0
2509.789	8	39831.99	−.005	58730 4	–	98562° 3				−.005	44655 4	–	83147° 5
2513.113	80	39779.30	.001	77557 2	–	117336° 2	2599.717	5	38454.23	.005	58730 4	–	97184° 4
2599.818	20	38452.73	.001	54853 5	–	93306° 6	2703.153	50	36982.86	.006	46601 4	–	83584° 3
2600.116	200	38448.33	.001	47978 2	–	86426° 2	2704.271	50	36967.57	−.001	56741 2	–	93709° 1
2604.433	30	38384.60	−.002	49088 2	–	87473° 2	2704.479	15	36964.73	.001	52811 1	–	89775° 0
2605.087	100	38374.96	−.003	42665 2	–	81040° 3	2704.632	5	36962.64	−.001	58893 3	–	95856° 3
2606.631	100	38352.23	.007	52697 4	–	91050° 3	2705.841	100	36946.13	−.002	42521 2	–	79467° 2
2609.580	15	38308.90	.003	52697 4	–	91006° 5	2709.180	1	36900.59	.002	56741 2	–	93642° 2
2609.699	1	38307.15	.000	50362 1	–	88669° 0	2709.750	80	36892.83	.003	43461 3	–	80354° 3
2610.804	80	38290.94	.001	58893 3	–	97184° 4	2712.237	10	36859.00	.004	54191 2	–	91050° 3
2611.181	5	38285.41	−.006	91098 4	–	129383° 4	2713.440	1	36842.66	−.008	42665 2	–	79508° 3
2611.825	15	38275.97	.005	55366 1	–	93642° 2	2716.151	200	36805.89	−.001	52697 4	–	89503° 4
2611.935	20	38274.36	.005	56741 2	–	95016° 1	2716.428	20	36802.14	.001	42665 2	–	79467° 2
26Ì2.188	8	38270.65	.008	93642° 2	–	131913 2	2717.127	30	36792.67	.004	43561 3	–	80354° 3
2613.687	50	38248.70	−.003	54853 5	–	93102° 4	2717.227	20	36791.32	.003	54191 2	–	90982° 2
2613.943	10	38244.96	−.002	46299 3	–	84544° 4	2718.088	1	36779.66	−.002	94292° 1	–	131072 2
2614.515	20	38236.59	.000	77557 2	–	115794° 2	2721.532	1	36733.12	−.004	55366 1	–	92099° 2
2614.998	80	38229.53	−.003	50362 1	–	88592° 2	2727.112	20	36657.97	.001	58893 3	–	95551° 2
2615.207	20	3S226 47	.001	64331 3	–	102557° 3	2728.896	100	36634.00	−.001	43461 3	–	80095° 4
2616.760	1	38203.79	.003	93709° 1	–	131913 2	2730.739	5	36609.28	−.001	42404 1	–	79013° 2
2618.972	20	38171.52	.001	52811 1	–	90982° 2	2731.761	1	36595.59	.005	48734 1	–	85329° 2
2622.319	30	38122.81	.001	50318 5	–	88441° 6	2731.853	1	36594.35	.002	49088 2	–	85683° 1
					–					−.005	94292° 1	–	130886 1
2623.350	10	38107.82	.003	58730 4	–	96838° 3						–	
2625.404	30	38078.01	−.005	51425 3	–	89503° 4	2733.391	300	36573.76	.002	50318 5	–	86892° 5
2625.483	5	38076.87	−.006	97184° 4	–	135261 4	2734.272	1	36561.98	−.003	96589° 2	–	133151 3
2626.908	1	38056.21	.007	94117° 3	–	132173 4	2735.456	20	36546.16	.001	46601 4	–	83147° 5
2628.662	100	38030.82	.001	54853 5	–	92884° 5	2735.684	50	36543.11	−.005	42404 1	–	78947° 1
2633.564	300	37960.04	−.001	50481 6	–	88441° 6	2736.383	50	36533.78	.005	43561 3	–	80095° 4
2634.751	20	37942.94	.000	46601 4	–	84544° 4	2739.505	15	36492.14	.000	42521 2	–	79013° 2
2637.155	20	37908.35	−.003	54191 2	–	92099° 2				.004	59059 2	–	95551° 2
2638.395	100	37890.53	−.003	52697 4	–	90588° 3	2742.344	1	36454.37	.000	48854 0	–	85308° 1
2639.435	50	37875.61	.000	54853 5	–	92728° 6	2744.485	50	36425.93	.000	42521 2	–	78947° 1
					–		2745.618	50	36410.90	.007	50481 6	–	86892° 5
2646.205	50	37778.71	.002	59059 2	–	96838° 3						–	
2652.004	2	37696.11	−.003	58893 3	–	96589° 2	2746.643	2	36397.31	−.007	54191 2	–	90588° 3
2652.524	50	37688.72	.000	42665 2	–	80354° 3	2746.838	10	36394.73	.002	54191 2	–	90586° 2
2654.762	10	37656.95	−.001	51482 2	–	89139° 1	2746.860	5	36394.44	−.002	106803° 3	–	143198 4
2655.545	20	37645.84	−.003	56741 2	–	94387° 2	2749.877	100	36354.51	.001	49541 4	–	85896° 4
2660.029	20	37582.39	.002	46962 5	–	84544° 4	2750.350	50	36348.26	.005	51482 2	–	87831° 1
2660.261	50	37579.11	−.003	43461 3	–	81040° 3	2750.350	50	36348.26	−.004	42665 2	–	79013° 2
2661.824	30	37557.05	.003	52697 4	–	90255° 4	2753.392	15	36308.10	−.002	55366 1	–	91674° 2
2661.975	20	37554.92	−.001	58730 4	–	96285° 5	2755.378	50	36281.93	.004	42665 2	–	78947° 1
2662.156	1	37552.36	.008	97709° 5	–	135261 4	2758.470	50	36241.27	−.001	49088 2	–	85329° 2
					–		2758.515	20bl	36240.67	−.009	46299 3	–	82540° 2
2662.270	20	37550.75	.001	56741 2	–	94292° 1						–	
2663.759	10	37529.77	.007	94117° 3	–	131647 2	2759.679	20	36225.39	−.001	58730 4	–	94955° 4
2667.374	80	37478.91	.001	43561 3	–	81040° 3	2760.073	10	36220.22	−.001	49088 2	–	85308° 1
2668.923	2	37457.16	.000	96838° 3	–	134295 3	2761.528	10	36201.14	−.010	106511° 4	–	142712 5
2672.918	30	37401.17	.000	54853 5	–	92254° 5	2762.711	200	36185.63	.002	46962 5	–	83147° 5
2674.739	50	37375.71	−.002	56741 2	–	94117° 3	2764.390	20	36163.66	.000	42404 1	–	78568° 0
2676.252	100	37354.58	.002	44655 4	–	82009° 4	2764.968	50	36156.10	.000	42521 2	–	78677° 1
2677.980	1	37330.48	−.001	47978 2	–	85308° 1	2765.165	20	36153.52	.002	54853 5	–	91006° 5
2681.251	10	37284.94	.000	46299 3	–	83584° 3	2768.461	2	36110.48	.008	54191 2	–	90301° 1
2689.819	30	37166.18	−.001	51425 3	–	88592° 2	2773.303	8	36047.44	−.003	51425 3	–	87473° 2
					–		2774.220	5	36035.52	−.004	43461 3	–	79497° 4
2692.734	20	37125.95	.002	58730 4	–	95856° 3						–	
2693.837	20	37110.75	−.003	50362 1	–	87473° 2	2775.666	5	36016.75	−.003	56741 2	–	92758° 3
			−.002	97184° 4	–	134295 3	2776.012	3	36012.26	−.008	42665 2	–	78677° 1
2693.949	15	37109.21	−.001	51482 2	–	88592° 2	2776.467	15	36006.36	−.004	43461 3	–	79467° 2
2696.560	10	37073.28	.002	51425 3	–	88499° 3	2777.691	10	35990.50	−.006	51482 2	–	87473° 2
2697.288	50	37063.27	−.004	42404 1	–	79467° 2	2780.036	100	35960.14	−.005	50362 1	–	86322° 0
2699.432	10	37033.84	.000	77557 2	–	114591° 3	2780.300	1	35956.73	−.001	59059 2	–	95016° 1
2700.709	20	37016.33	.001	51482 2	–	88499° 3	2781.100	50	35946.38	−.010	58730 4	–	94676° 3
2702.492	200	36991.91	.003	52697 4	–	89689° 5	2781.957	50	35935.31	.002	43561 3	–	79497° 4
2702.887	100	36986.50	.000	42521 2	–	79508° 3	2784.005	1	35908.88	.003	51482 2	–	87391° 3
					–		2784.215	100	35906.17	.000	43561 3	–	79467° 2
2785.561	10	35888.82	−.002	64331 3	–	100219° 2	2953.595	50	33847.15	−.003	51482 2	–	85329° 2
2792.374	100	35801.26	−.001	52697 4	–	88499° 3	2953.857	50	33844.15	−.004	56741 2	–	90586° 2
2793.950	1	35781.07	−.005	52811 1	–	88592° 2	2955.436	20	33826.07	.000	51482 2	–	85308° 1
2799.485	10	35710.33	−.001	46299 3	–	82009° 4	2957.206	30	33805.82	−.001	48734 1	–	82540° 2
2800.358	300	35699.19	.001	44655 4	–	80354° 3	2958.059	1	33796.07	−.004	46299 3	–	80095° 4
2801.248	100	35687.85	.004	44655 4	–	80343° 5	2961.844	20	33752.89	.002	46601 4	–	80354° 3
2806.498	5	35621.10	−.001	64331 3	–	99952° 2	2962.833	20	33741.62	−.001	46601 4	–	80343° 5
2806.887	1	35616.16	−.003	55366 1	–	90982° 2	2971.788	50	33639.95	.000	54191 2	–	87831° 1
2809.944	20	35577.41	−.003	50318 5	–	85896° 4	2976.357	20	33588.31	−.001	54853 5	–	88441° 6
2811.924	20	35552.36	−.001	43461 3	–	79013° 2	2982.066	1	33524.01	.003	58730 4	–	92254° 5
2816.554	10	35493.92	−.004	58893 3	–	94387° 2	2983.171	20	33511.59	.000	52811 1	–	86322° 0
2816.646	5	35492.76	−.009	42665 2	–	78158° 3	2983.927	300	33503.10	.003	44655 4	–	78158° 3
2819.868	30	35452.21	.000	43561 3	–	79013° 2	2984.740	20	33493.98	.004	46601 4	–	80095° 4
2820.811	5	35440.36	−.003	44655 4	–	80095° 4	2988.627	200bl	33450.42	.010	42521 2	–	75972° 1
2823.887	10	35401.76	−.004	54853 5	–	90255° 4	2994.838	100	33381.05	.003	46962 5	–	80343° 5
2825.048	20	35387.21	−.008	58730 4	–	94117° 3	2995.362	100	33375.21	−.001	43461 3	–	76836° 2
2826.591	15	35367.89	−.009	58730 4	–	94098° 4	3001.542	100	33306.49	.008	42665 2	–	75972° 1
2829.187	1	35335.44	.009	96838° 3	–	132173 4	3003.749	80	33282.02	.003	54191 2	–	87473° 2
2837.408	15	35233.07	−.001	59059 2	–	94292° 1	3004.382	200	33275.01	.004	43561 3	–	76836° 2
2838.155	15	35223.79	−.003	58893 3	–	94117° 3	3008.851	100	33225.59	.000	55366 1	–	88592° 2
2838.497	100	35219.55	−.002	55366 1	–	90586° 2	3010.377	15	33208.75	.000	46299 3	–	79508° 3
2839.716	3	35204.43	.000	58893 3	–	94098° 4	3010.653	2	33205.70	.002	58893 3	–	92099° 2
2852.410	10	35047.77	.002	46962 5	–	82009° 4	3011.120	5	33200.55	−.001	54191 2	–	87391° 3
2854.659	50	35020.16	−.002	52811 1	–	87831° 1	3011.331	40	33198.23	.001	52697 4	–	85896° 4
2856.066	1	35002.91	−.005	49541 4	–	84544° 4	3014.045	1	33168.34	.001	46299 3	–	79467° 2
2856.227	5	35000.94	−.003	51425 3	–	86426° 2	3018.571	200	33118.61	.001	51425 3	–	84544° 4
			.006	98562° 3	–	133563 2	3023.722	5	33062.19	.002	47978 2	–	81040° 3
2858.969	40	34967.37	.003	50362 1	–	85329° 2	3039.040	20	32895.55	−.003	46601 4	–	79497° 4
2860.687	1	34946.37	−.001	50362 1	–	85308° 1	3041.209	10	32872.09	−.004	52811 1	–	85683° 1
2861.591	8	34935.33	.002	55366 1	–	90301° 1	3049.684	1	32780.74	−.002	58893 3	–	91674° 2
2861.806	15	34932.71	−.004	56741 2	–	91674° 2	3058.638	1	32684.78	.000	72356 2	–	105041° 1
2868.343	100	34853.10	−.004	44655 4	–	79508° 3	3060.380	30	32666.18	−.004	50481 6	–	83147° 5
2869.272	30	34841.81	.000	44655 4	–	79497° 4	3061.228	10	32657.13	−.010	58730 4	–	91387° 4
2869.701	300	34836.61	−.003	54853 5	–	89689° 5	3065.181	1	32615.02	−.007	59059 2	–	91674° 2
2873.607	1	34789.26	.004	96589° 2	–	131379 2	3070.415	8	32559.42	−.001	72481 1	–	105041° 1
2877.580	200	34741.22	−.010	46299 3	–	81040° 3	3074.234	100	32518.98	−.005	52811 1	–	85329° 2
2884.135	20	34662.27	−.003	52811 1	–	87473° 2	3076.230	30	32497.88	.000	52811 1	–	85308° 1
2885.109	10	34650.57	−.005	54853 5	–	89503° 4	3079.367	15	32464.77	−.004	55366 1	–	87831° 1
2889.614	200	34596.55	.009	43561 3	–	78158° 3	3080.010	100	32457.99	.000	44655 4	–	77113° 5
2890.752	20	34582.93	−.001	59059 2	–	93642° 2	3085.723	5	32397.90	.001	56741 2	–	89139° 1
2898.053	50	34495.81	−.001	49088 2	–	83584° 3	3089.823	30	32354.91	−.003	43461 3	–	75816° 4
2900.205	3	34470.22	.007	51425 3	–	85896° 4	3097.338	10	32276.42	.001	58730 4	–	91006° 5
2902.827	20	34439.08	.002	46601 4	–	81040° 3	3097.407	10	32275.70	.001	53407 0	–	85683° 1
2904.114	80	34423.82	−.002	53407 0	–	87831° 1	3099.422	50	32254.71	.003	43561 3	–	75816° 4
2905.337	50	34409.33	.000	55366 1	–	89775° 0	3101.223	1	32235.98	.007	103621° 5	–	135857 4
2906.054	50	34400.84	−.001	54191 2	–	88592° 2	3101.264	10	32235.56	.001	54191 2	–	86426° 2
2908.527	5	34371.59	−.005	58730 4	–	93102° 4	3106.860	100	32177.50	.006	50362 1	–	82540° 2
2913.324	80	34315.00	−.001	42521 2	–	76836° 2	3108.682	15	32158.64	−.001	51425 3	–	83584° 3
2913.886	50	34308.38	.002	56741 2	–	91050° 3	3108.892	3	32156.47	.001	58893 3	–	91050° 3
2918.814	20	34250.46	−.007	99313° 1	–	133563 2	3109.068	10	32154.65	.000	72356 2	–	104511° 2
2919.645	20	34240.71	−.001	56741 2	–	90982° 2	3113.699	1	32106.83	.000	55366 1	–	87473° 2
2922.419	1	34208.21	−.002	58893 3	–	93102° 4	3114.201	50	32101.65	.001	51482 2	–	83584° 3
2923.102	5	34200.22	.001	51482 2	–	85683° 1	3118.345	8	32058.99	−.004	42665 2	–	74724° 3
2923.576	30	34194.67	−.002	52697 4	–	86892° 5	3120.263	3	32039.29	−.001	54853 5	–	86892° 5
2925.595	50	34171.08	−.003	42665 2	–	76836° 2	3138.795	100	31850.13	.005	56741 2	–	88592° 2
2935.571	3	34054.96	−.005	46299 3	–	80354° 3	3154.194	5	31694.64	.000	58893 3	–	90588° 3
2948.630	30	33904.14	−.005	51425 3	–	85329° 2	3154.552	20	31691.04	.003	50318 5	–	82009° 4
2952.054	10	33864.82	−.003	58893 3	–	92758° 3	3167.977	10	31556.75	.009	46601 4	–	78158° 3
2952.765	8	33856.66	.003	62879 0	–	96736° 1	3174.514	1	31491.77	.010	54191 2	–	85683° 1
2953.007	50	33853.89	−.006	50362 1	–	84216° 0	3175.761	15	31479.40	.006	72187 3	–	103667° 3
3183.256	100	31405.29	.006	52811 1	–	84216° 0	3220.417	3	31042.91	−.006	54853 5	–	85896° 4
3187.736	200bl	31361.15	.007	58893 3	–	90255° 4	3229.446	20	30956.12	.007	55366 1	–	86322° 0
			−.008	103303° 2	–	134665 3							
3190.772	30	31331.31	.003	42521 2	–	73853° 2							
3197.456	20	31265.82	−.007	49088 2	–	80354° 3							
3197.733	1	31263.11	−.002	43461 3	–	74724° 3							
3208.009	10	31162.97	−.002	43561 3	–	74724° 3							
3210.507	300	31138.73	.002	54191 2	–	85329° 2							
3212.678	50	31117.69	.001	54191 2	–	85308° 1							
3218.921	5	31057.34	−.007	51482 2	–	82540° 2							

a Symbols following are: h = hazy, bl = blended.

**Table 4:** Least-squares fitted (LSF) and Hartree-Fock with relativistic corrections (HFR) parameter values and their ratios for the 4*d*^4^, 4*d*^3^ 5*s* and 4*d*^2^ 5*s*^2^ configurations of doubly ionized molybdenum (Mo III) in cm^−1^

Config.	Parameter	LSF	HFR	LSF/HFR
4*d*^4^	*E*_av_	19370(12)		
	*F*^2^(*dd*)	45688(35)	58323	0.783
	*F*^4^(*dd*)	30027(76)	37994	0.790
	ζ_4_*_d_*	699(8)	700	0.999
	α	31(1)		
	*β*	−237(21)		
4*d*^3^ 5*s*	*E*_av_	49995(10)	49865	1.003
	*F*^2^(*dd*)	48283(40)	61345	0.787
	*F*^4^(*dd*)	32087(81)	40177	0.799
	*G*^2^(*ds*)	11882(30)	15321	0.776
	ζ_4_*_d_*	756(9)	768	0.984
	α	23(1)		
	*β*	−102(20)		a
4*d*^2^ 5*s*^2^	*E*_av_	95745(30)	99404	0.963
	*F*^2^(*dd*)	51728(220)	64131	0.807
	*F*^4^(*dd*)	32720(fixed)	42200	0.775
	ζ_4_*_d_*	850(fixed)	837	1.015
	α	30(fixed)		
	*β*	−102(20)		a
CI	*R*^2^(*dd,ds*)^c^	−12107(150)	−17754	0.686^b^
	*R*^2^(*dd,ss*)	12684(160)	18454	0.686^b^
	*R*^2^(*dd,ds*)^c^	−11868(150)	−17253	0.686^b^
		Standard deviation of the level fit = 44 cm^−1^		

a The values of *ß* for 4*d*^3^ 5*s* and 4*d*^2^ *5s*^2^ were held equal to each other.

b The values of the *R*^2^ parameters were restricted to have the same LSQ/HFR ratios.

c The first *R*^2^(*dd,ds*) is the interaction parameter between 4*d*^4^ and 4*d*^3^ 5*s*. The second *R*^2^(*dd,ds*) is for the 4*d*^3^ 5*s*-4*d*^2^ 5*s*^2^ interaction.

**Table 5:** Least-square fitted (LSF) and Hartree-Fock with relativistic corrections (HFR) parameter values and their ratios for the 4*d*^3^ 6*s* and *4d^3^* 5*d* configurations of doubly ionized molybdenum (Mo III) in cm^−l^.

Config.	Parameter	LSF	HFR	LSF/HFR
4*d*^3^ 6*s*	*E*_av_	146417(17)	144393	1.016
	*F*^2^(*dd*)	48943(60)	62696	0.781
	*F*^4^(*dd*)	30828(220)	41170	0.749
	*G*^2^(*ds*)	2247(36)	2861	0.785
	ζ_4_*_d_*	804(11)	792	1.015
	α	41(3)		
*td^3^5d*	*E*_av_	147035(41)	144217	1.022
	*F*^2^(*dd*)	49036(77)	62758	0.781
	*F*^4^(*dd*)	30803(120)	41218	0.747
	*F*^2^(4*d*,5*d*)	8598(90)	10145	0.848
	*F*^4^(4*d*,5*d*)	3219(120)	4438	0.725
	*G*^0^(4*d*,5*d*)	2396(70)	3456	0.693
	*G*^2^(4*d*,5*d*)	2053(80)	3466	0.592
	*G*^4^(4*d*,5*d*)	1614(110)	2702	0.597
	ζ_4_*_d_*	804(6)	793	1.014
	ζ_5_*_d_*	102(9)	80	1.275
	α	48(2)		
CI	*R*^2^(4*d*5*d*,4*d*6*s*)	−2164(100)	−2747	0.790^a^
	*R*^2^(4*d*5*d*,6*s*4*d*)	−112(5)	−141	0.790^a^
		Standard deviation of the level fit = 33 cm^−1^		

a The CI parameters were constrained to have the same LSQ/HFR ratios.

**Table 6:** Least-squares fitted (LSF) and Hartree-Fock with relativistic corrections (HFR) parameter values and their ratios for the 4*d*^3^ 5*p* and *4d*^2^ 5*s*5*p* configurations of doubly ionized molybdenum (Mo III) in cm^−1^.

Config.	Parameter	LSF	HFR	LSF/HFR
4*d*^3^5*p*	*E*_av_	93015(37)	91474	1.017
	*F*^2^(*dd*)	48622(120)	61908	0.785
	*F*^2^(*dd*)	31578(260)	40591	0.780
	*F*^2^(*dp*)	16820(210)	21158	0.795
	*G*^1^(*dp*)	7189(80)	8947	0.804
	*G*^1^(*dp*)	4267(210)	7464	0.572
	ζ_4_*_d_*	832(24)	779	1.068
	ζ_5_*_p_*	1529(49)	1217	1.256
	*α*	35(4)		
	*β*	212(62)^a^		
4*d*^2^5*s*5*p*	*E*_av_	138238(50)	136752	1.011
	*F*^2^(*dd*)	51906(340)	64574	0.804
	*F*^2^(*dd*)	36428(550)	42528	0.857
	*F*^2^(*dp*)	19456(230)	22847	0.852
	*G*^2^(*ds*)	11844(350)	15246	0.777
	*G*^1^(*dp*)	8003(190)	9207	0.869
	*G*^3^(*dp*)	5605(500)	7900	0.709
	*G*^1^(*sp*)	24399(180)	42122	0.579
	ζ_4_*_d_*	849(28)	847	1.002
	ζ_5_*_p_*	1841(90)	1470	1.249
	*α*	30(fixed)		
	*β*	−212(62)		a
CI	*R*^2^(*dd*,*ds*)	−10683(500)	−17155	0.623^b^
	*R*^2^(*dp*,*sp*)	−11097(520)	−17820	0.623^b^
	*R*^2^(*dp*,*ps*)	−10779(500)	−17302	0.623^b^
		Standard deviation of the level fit = 183 cm^−1^		

a The values of *β* for the two configurations were constrained to be equal.

b The CI parameters were constrained to have the same LSF/HFR ratios.
